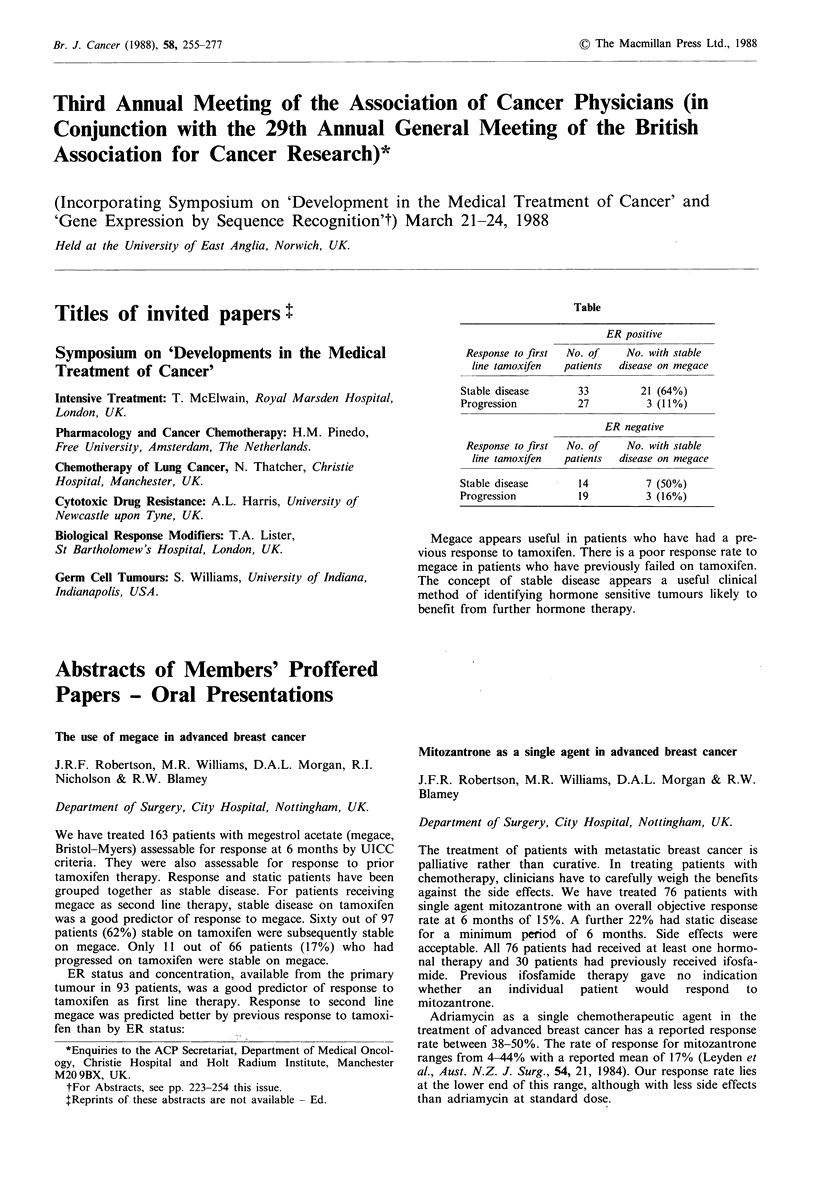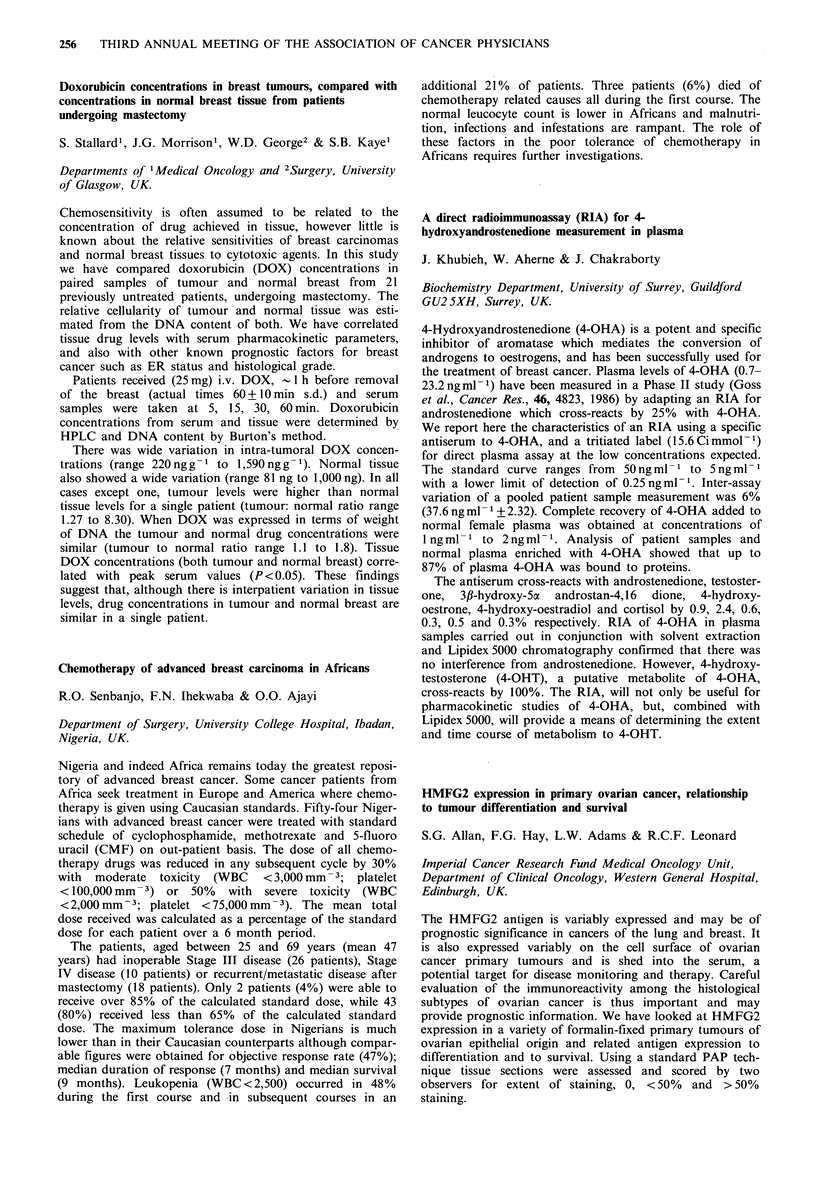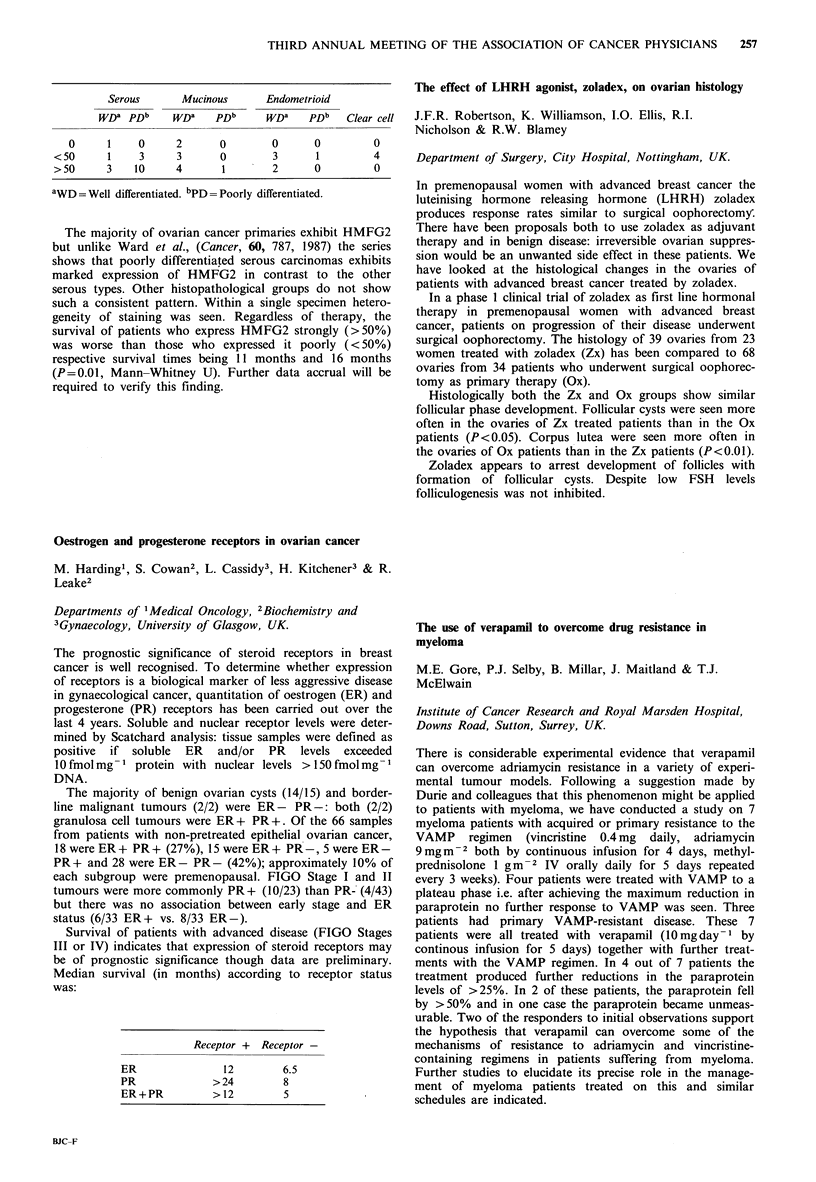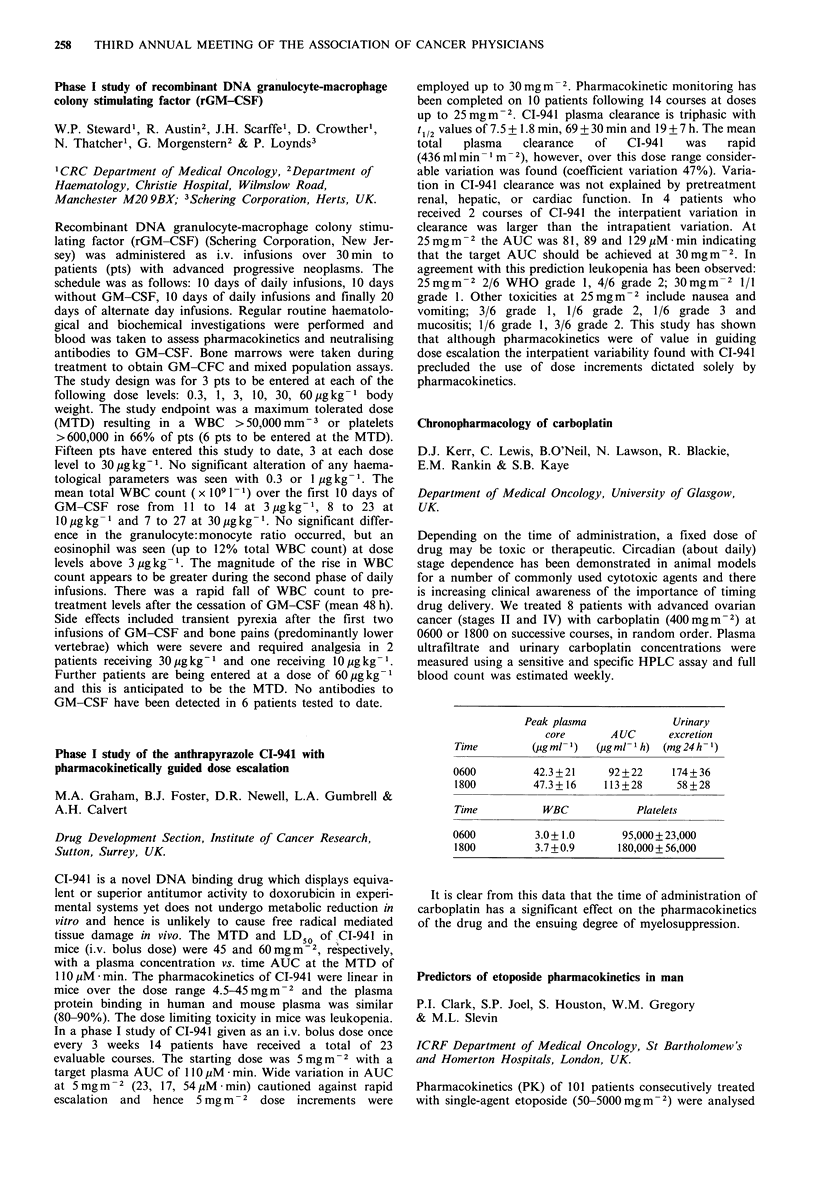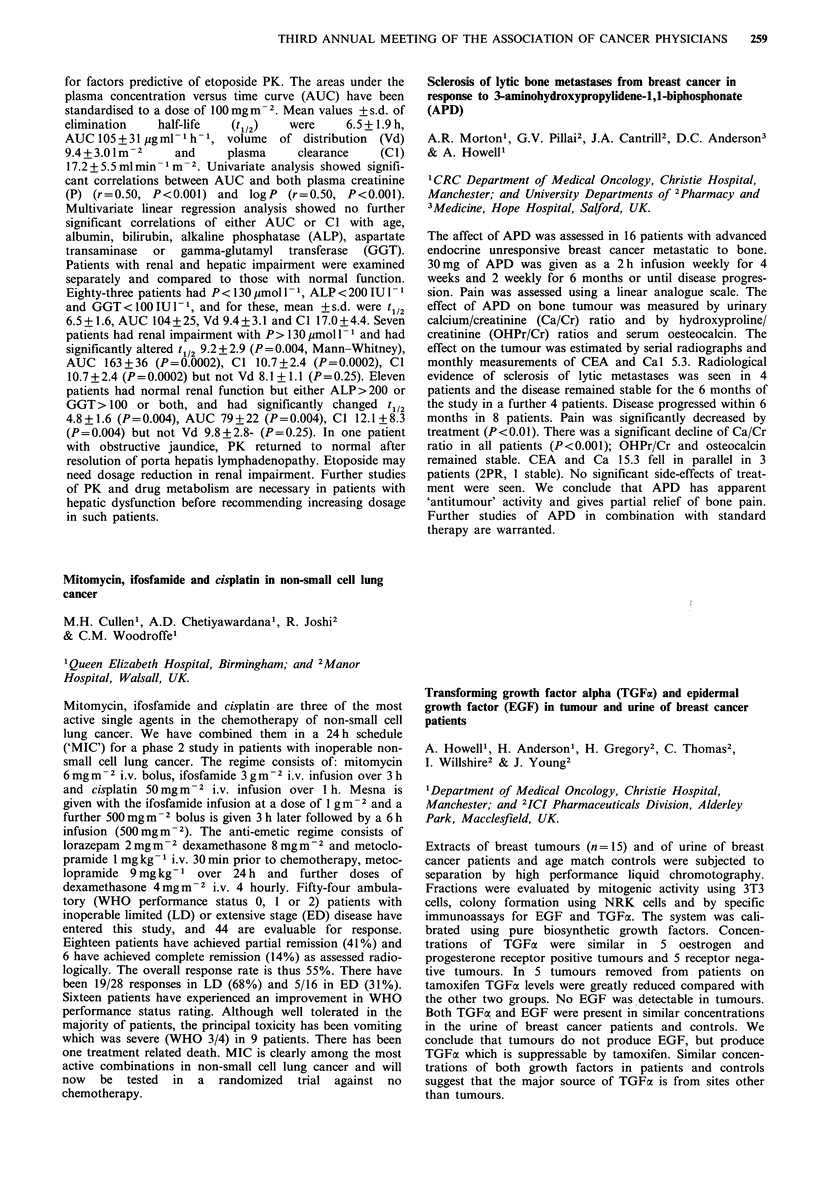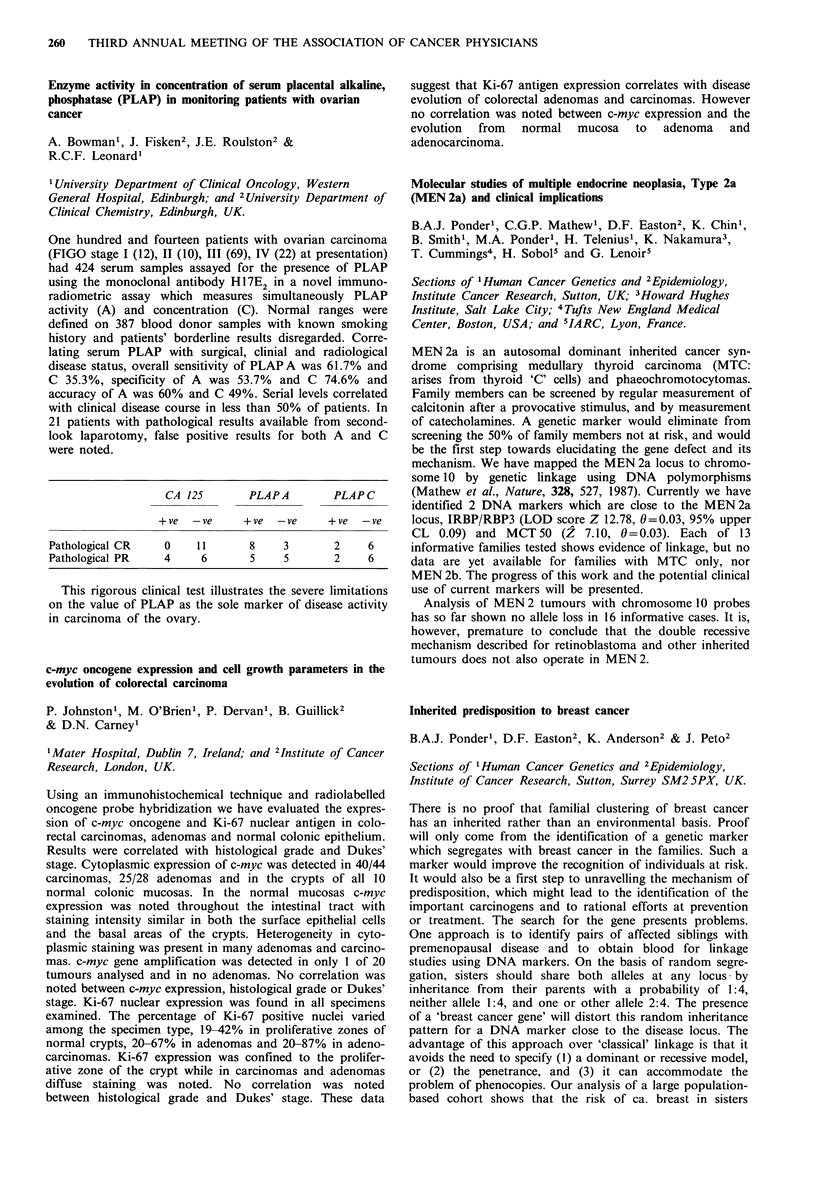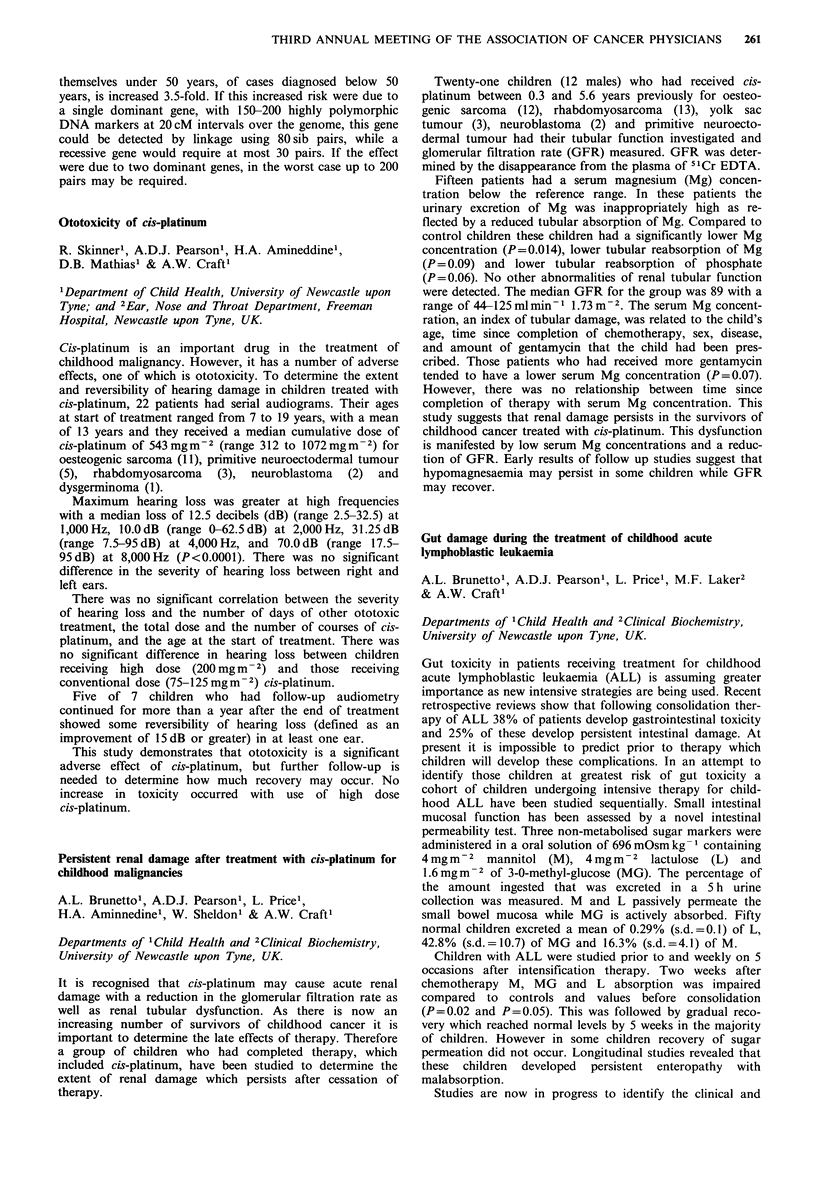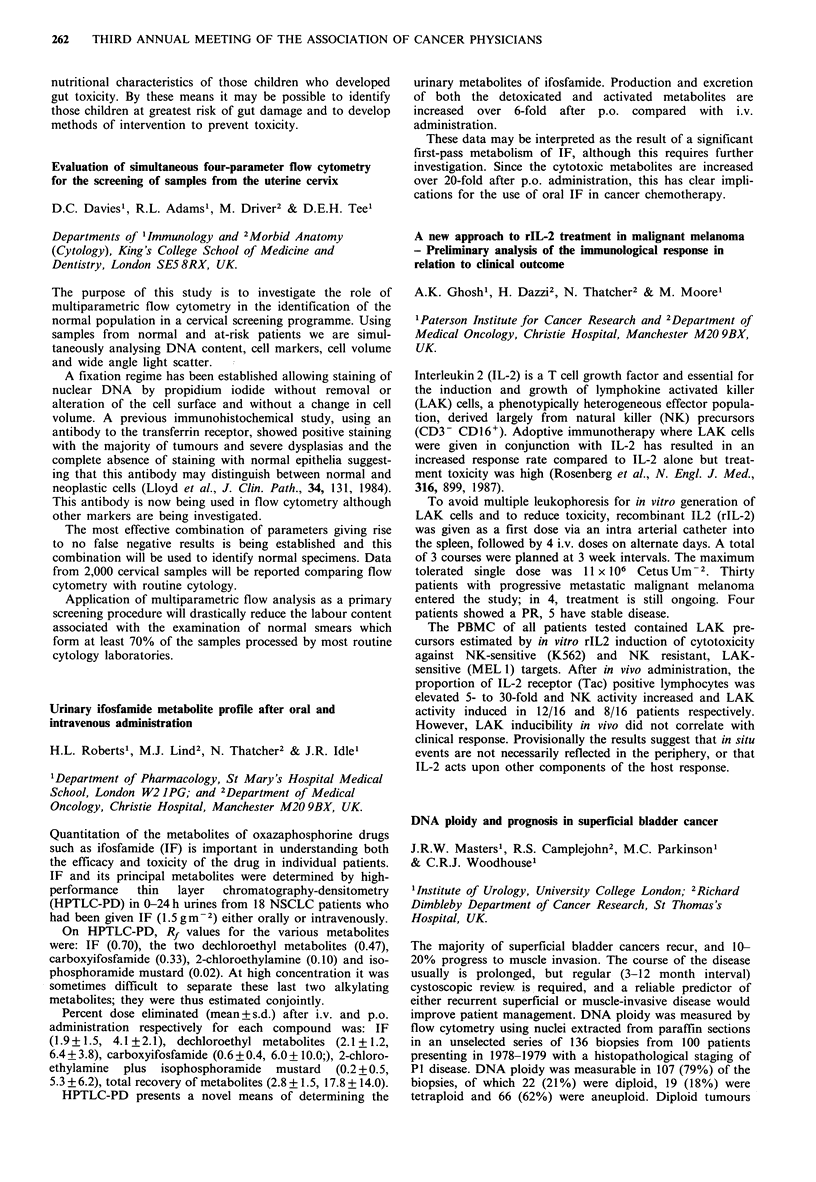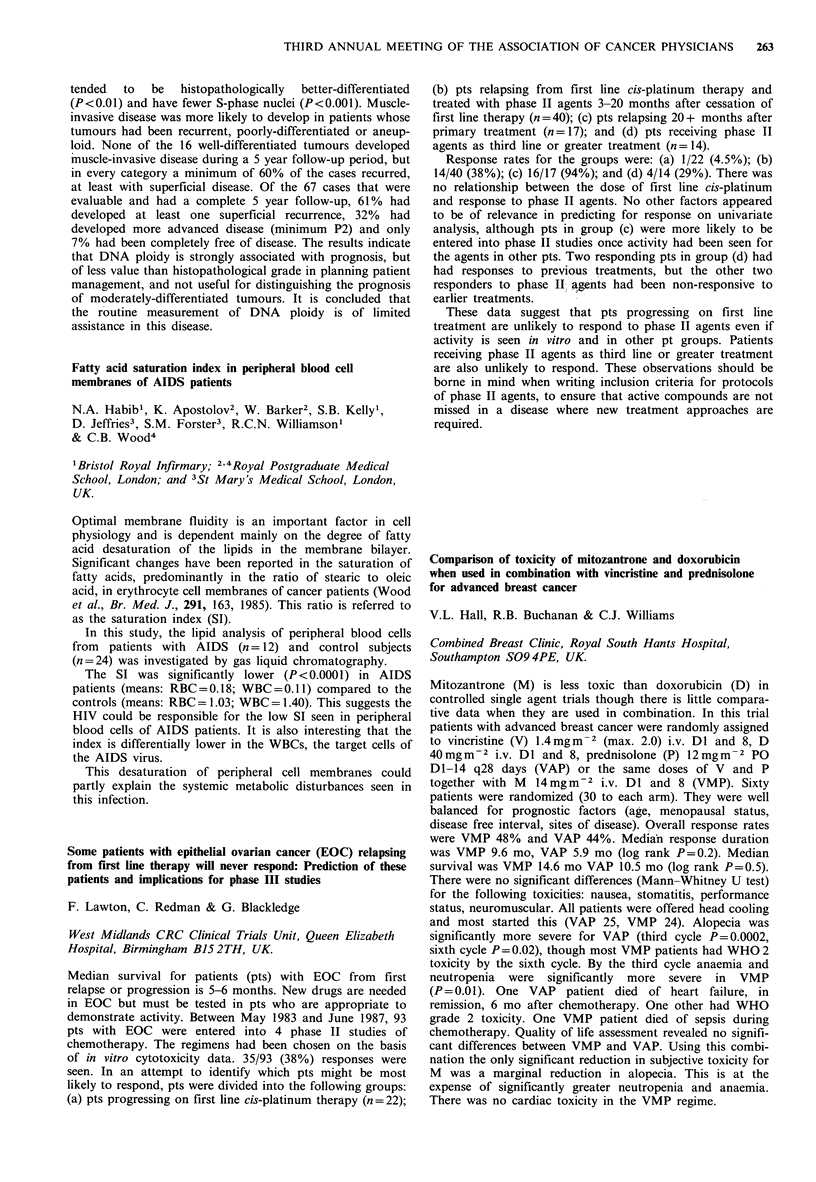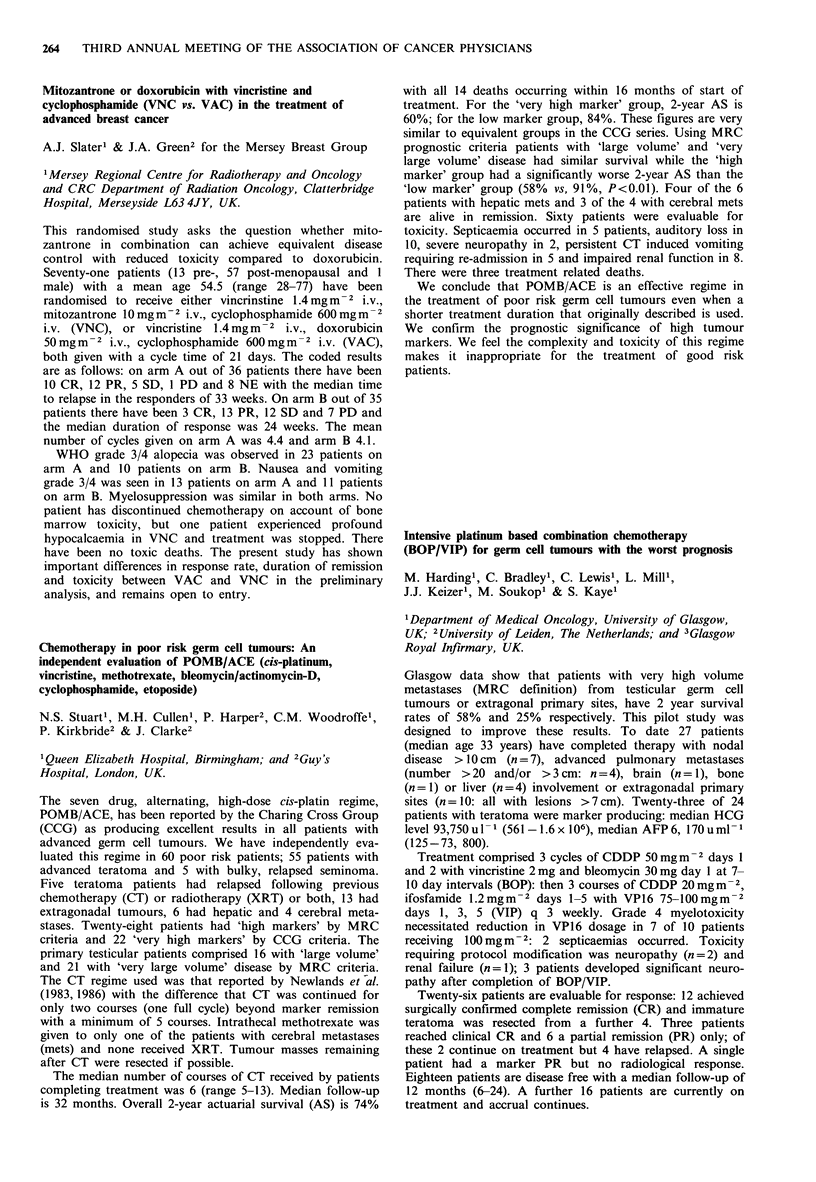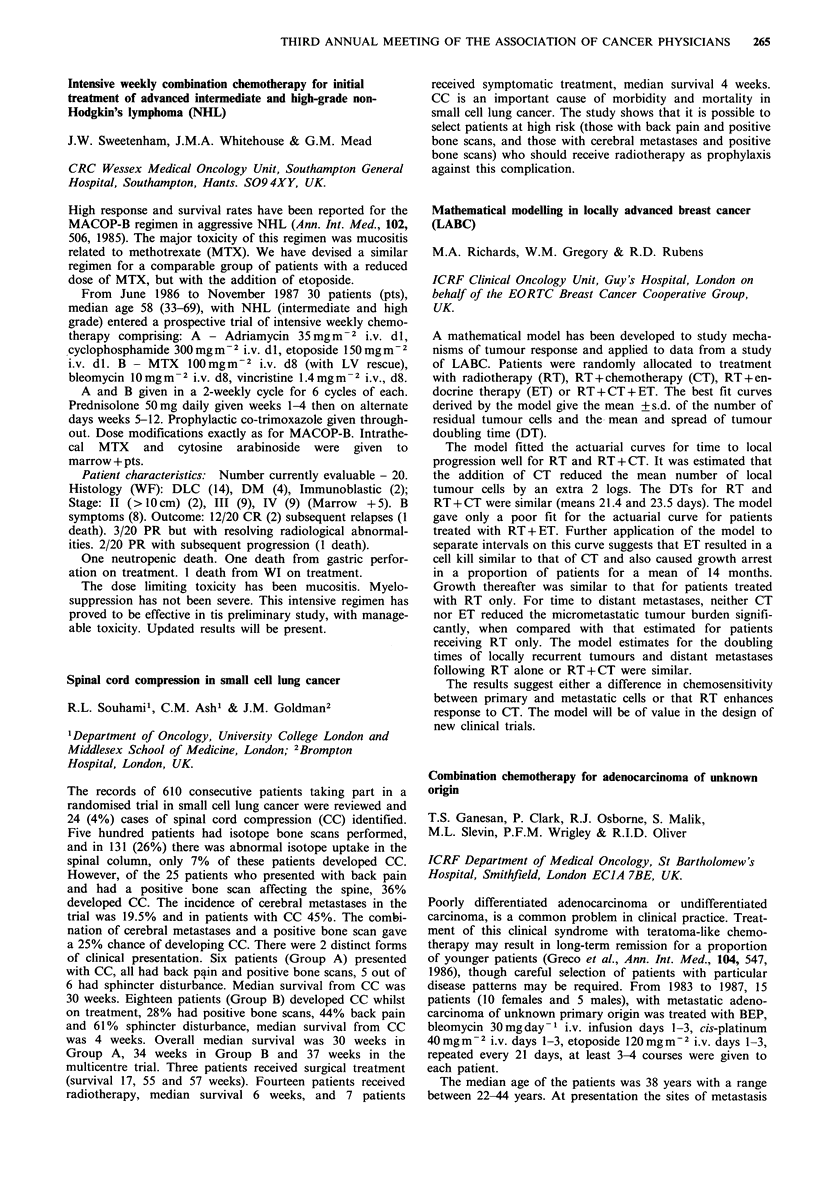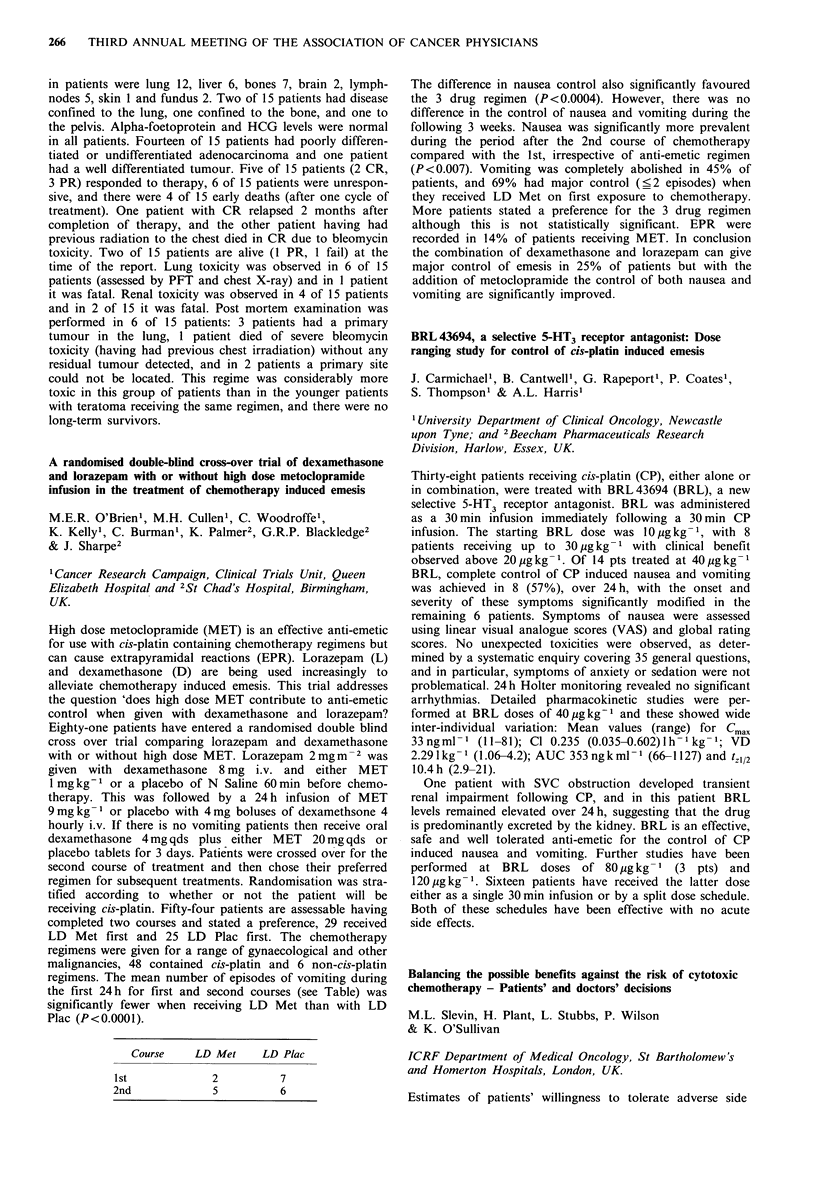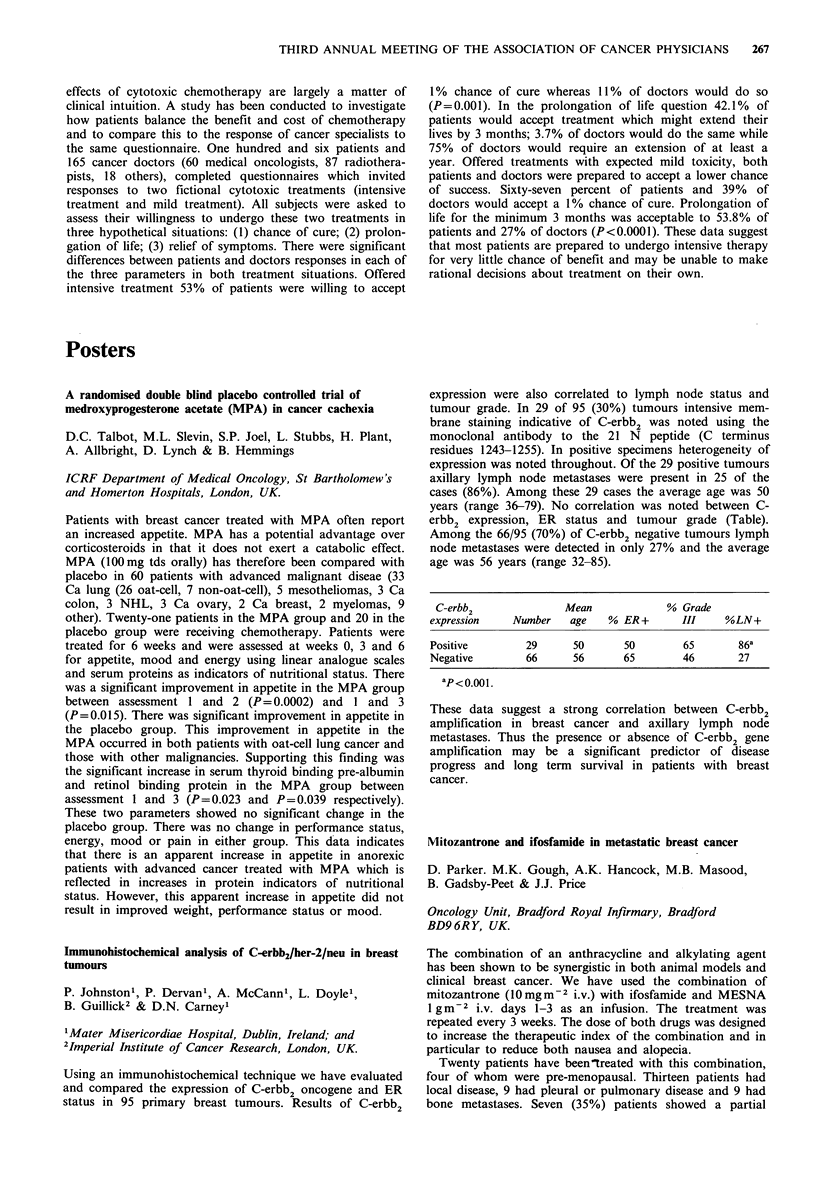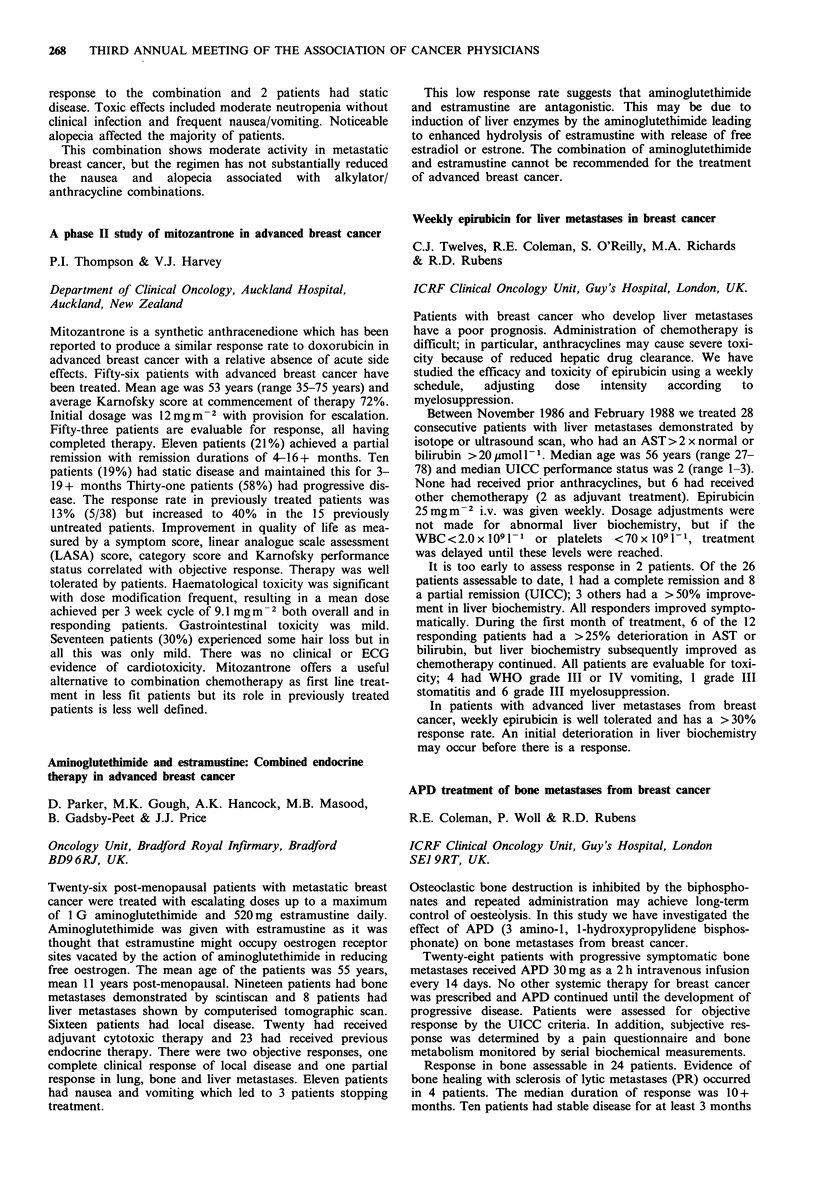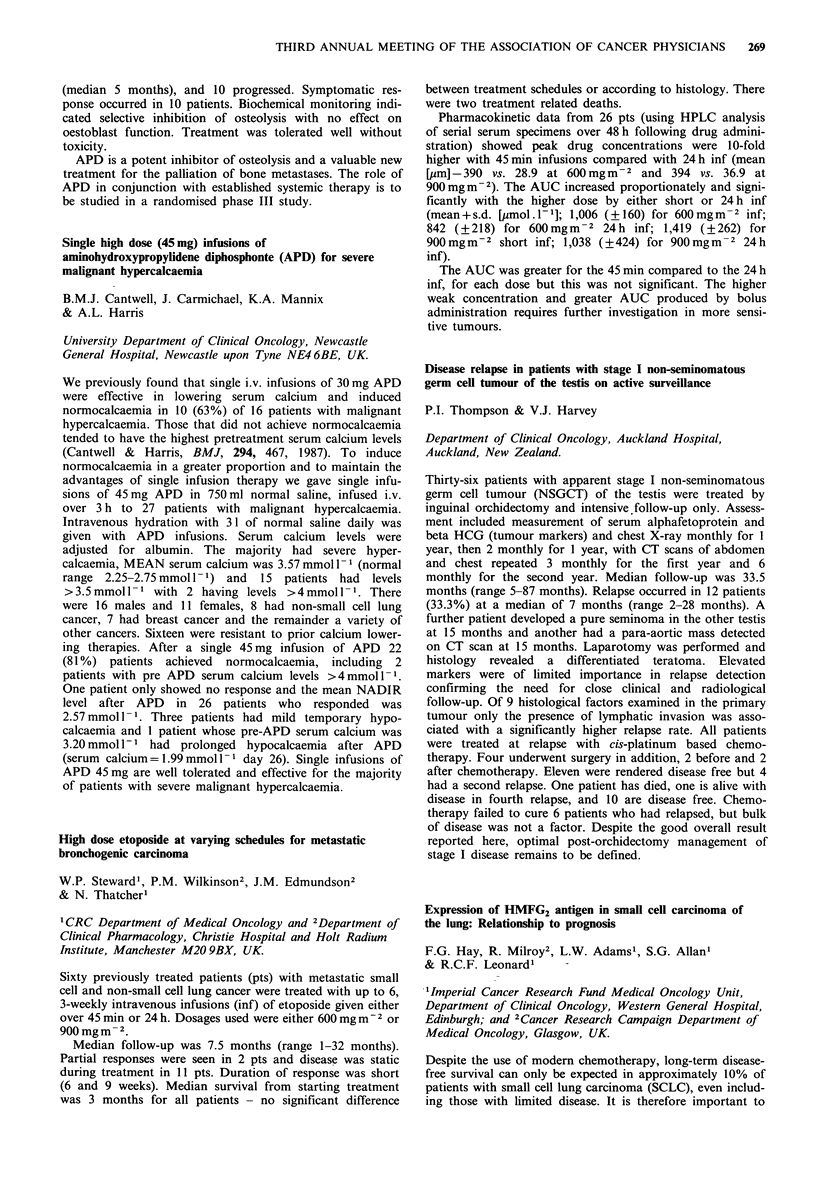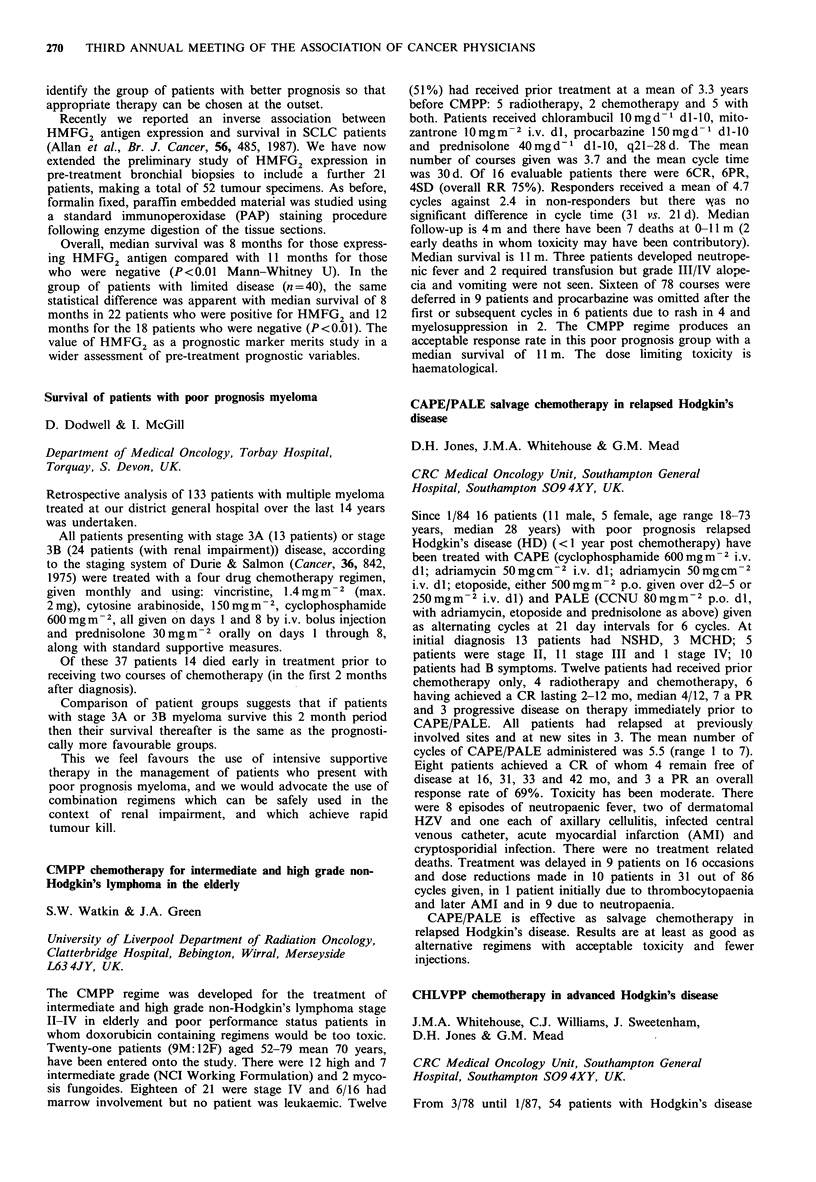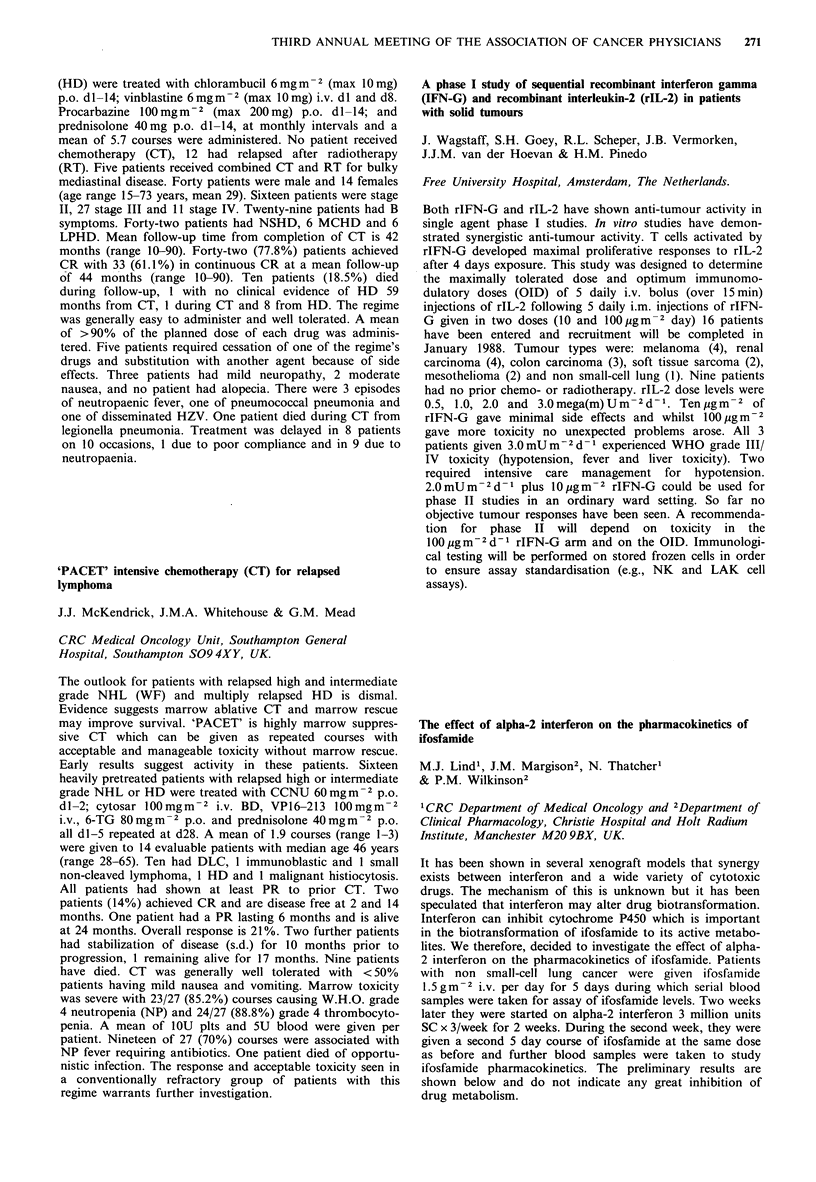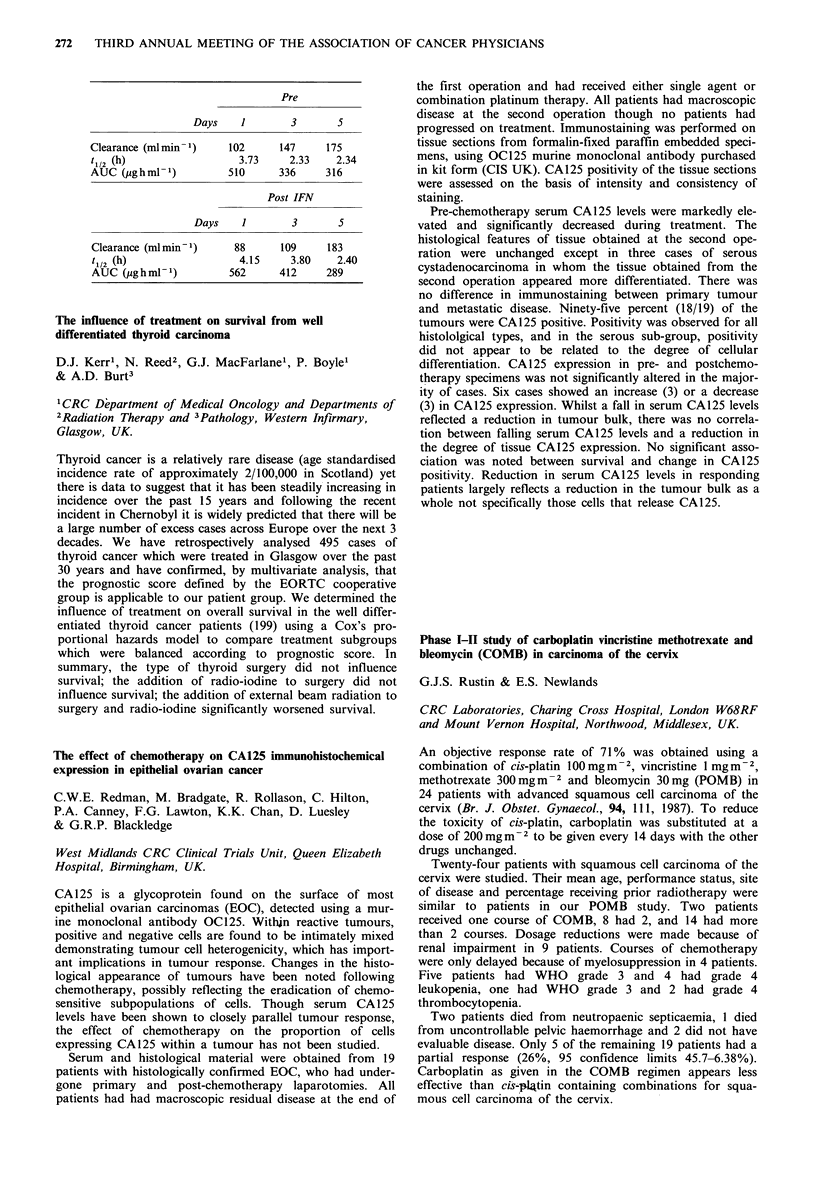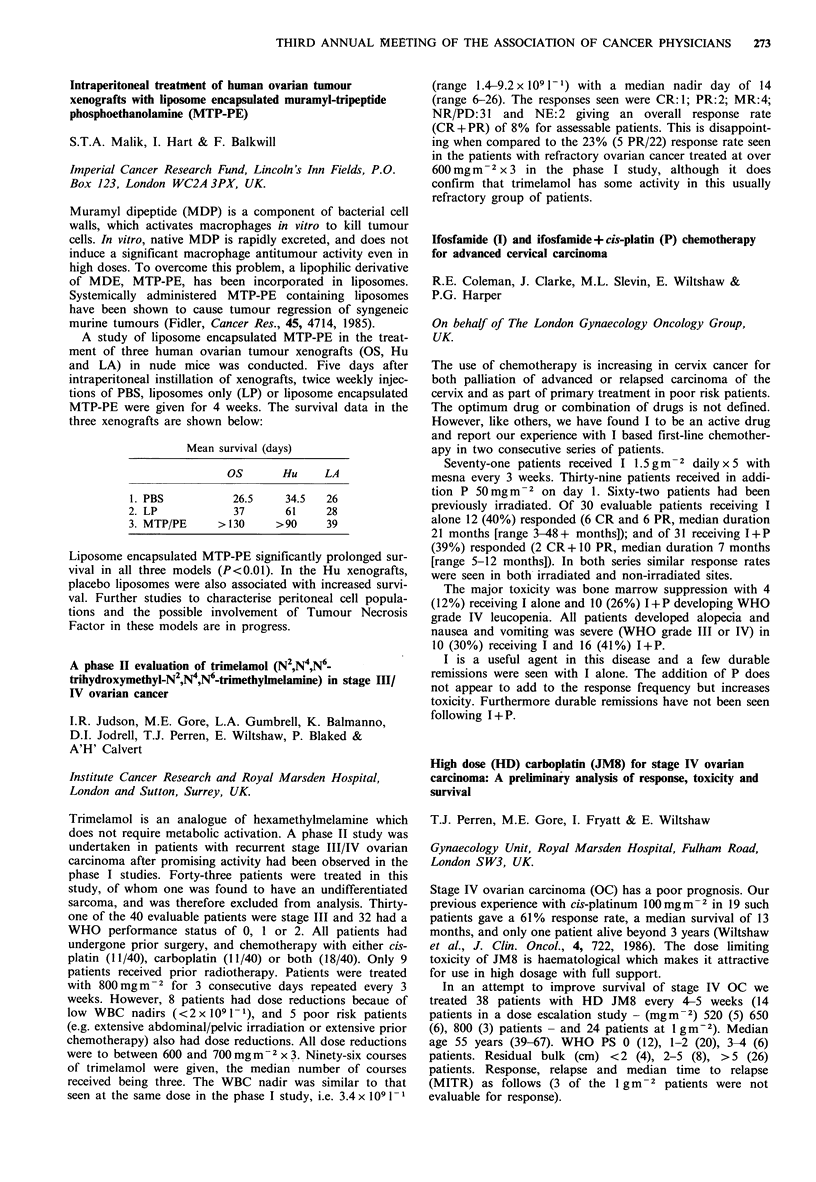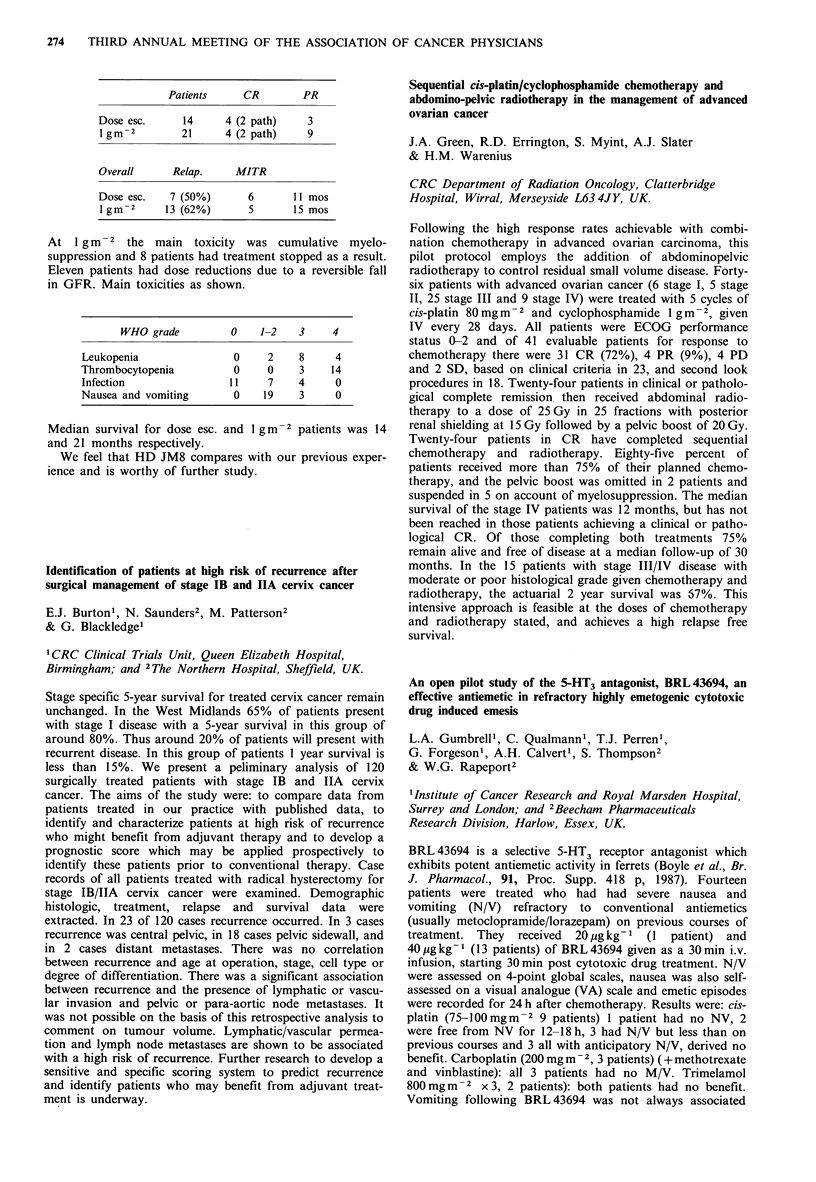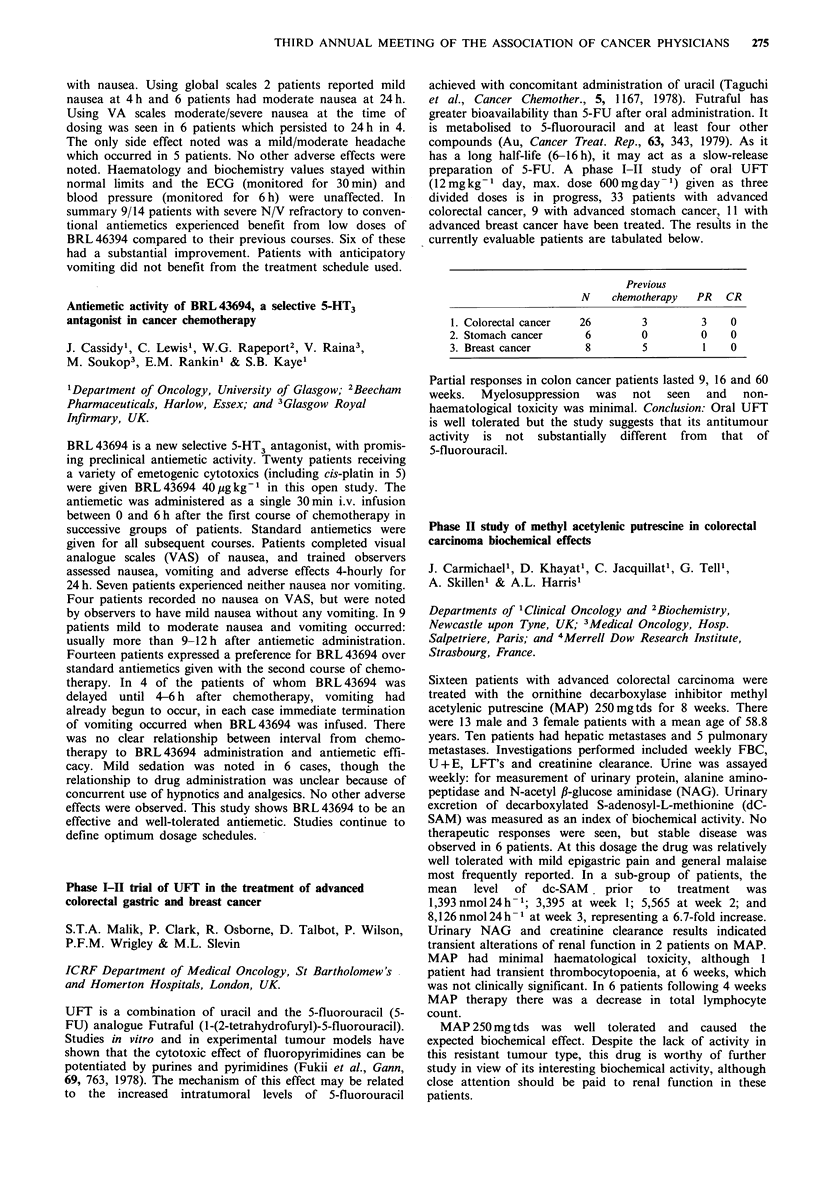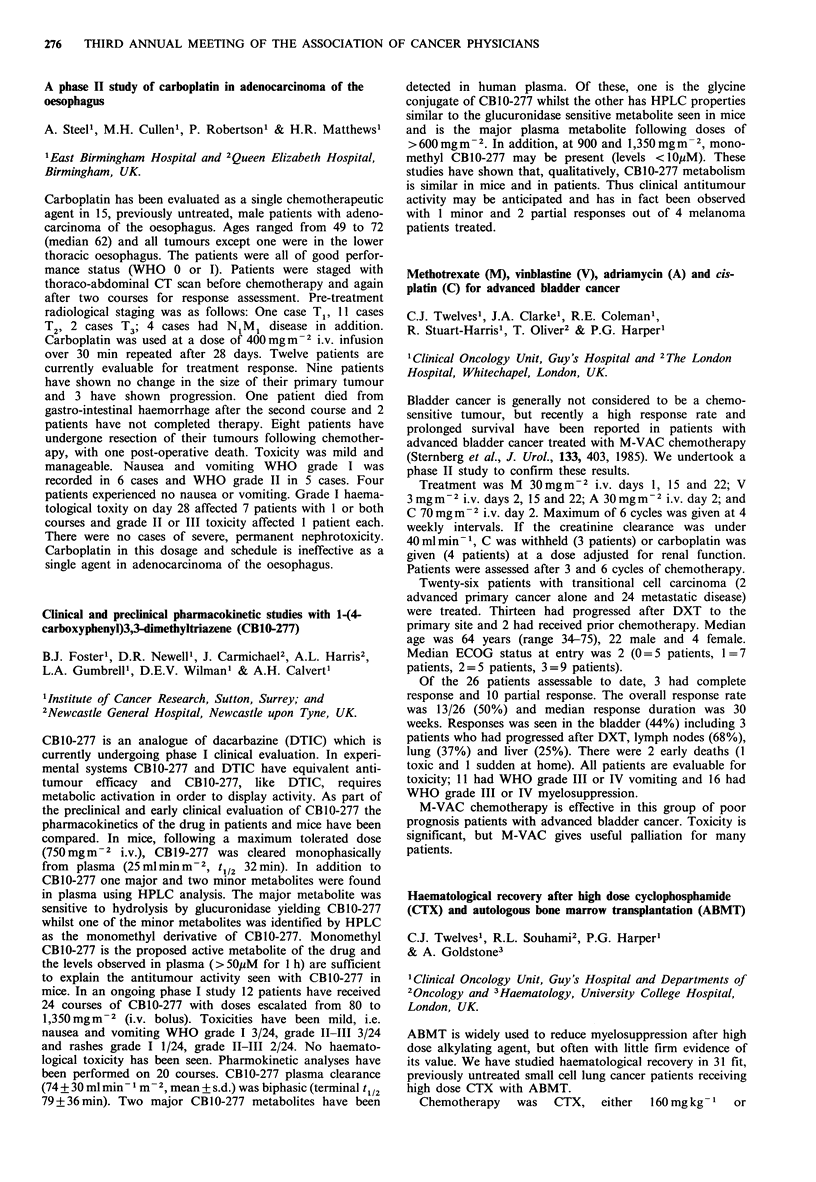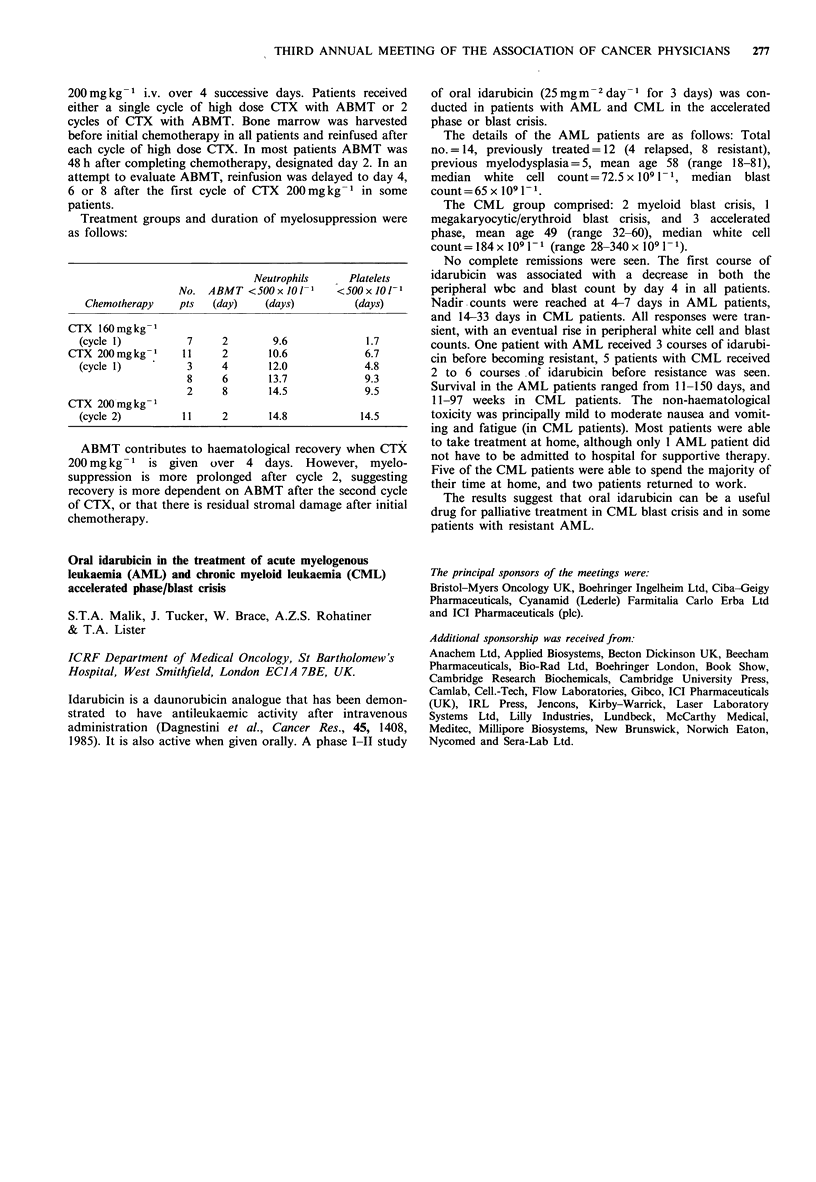# Third Annual Meeting of the Association of Cancer Physicians—22/24 March 1988. Abstracts of the proceedings*

**Published:** 1988-08

**Authors:** 


Br. J. Cancer (1988), 58, 255–277 © The Macmillan Press Ltd., 1988

### Third Annual Meeting of the Association of Cancer Physicians (in Conjunction with the 29th Annual General Meeting of the British Association for Cancer Research)*

(Incorporating Symposium on 'Development in the Medical Treatment of Cancer' and 'Gene Expression by Sequence Recognition'†) March 21–24, 1988

*Held at the University of East Anglia, Norwich, UK.*

### Titles of invited papers ‡

### Symposium on 'Developments in the Medical Treatment of Cancer'

**Intensive Treatment:** T. McElwain, *Royal Marsden Hospital, London, UK.*

**Pharmacology and Cancer Chemotherapy:** H.M. Pinedo, *Free University, Amsterdam, The Netherlands.*

**Chemotherapy of Lung Cancer,** N. Thatcher, *Christie Hospital, Manchester, UK.*

**Cytotoxic Drug Resistance:** A.L. Harris, *University of Newcastle upon Tyne, UK.*

**Biological Response Modifiers:** T.A. Lister, *St Bartholomew's Hospital, London, UK.*

**Germ Cell Tumours:** S. Williams, *University of Indiana, Indianapolis, USA.*

### Abstracts of Members' Proffered Papers - Oral Presentations

### The use of megace in advanced breast cancer

J.R.F. Robertson, M.R. Williams, D.A.L. Morgan, R.I. Nicholson & R.W. Blamey

*Department of Surgery, City Hospital, Nottingham, UK.*

We have treated 163 patients with megestrol acetate (megace, Bristol–Myers) assessable for response at 6 months by UICC criteria. They were also assessable for response to prior tamoxifen therapy. Response and static patients have been grouped together as stable disease. For patients receiving megace as second line therapy, stable disease on tamoxifen was a good predictor of response to megace. Sixty out of 97 patients (62%) stable on tamoxifen were subsequently stable on megace. Only 11 out of 66 patients (17%) who had progressed on tamoxifen were stable on megace.

ER status and concentration, available from the primary tumour in 93 patients, was a good predictor of response to tamoxifen as first line therapy. Response to second line megace was predicted better by previous response to tamoxifen than by ER status:

**Table**

| | ER positive | |
|---|---|---|
| *Response to first line tamoxifen* | *No. of patients* | *No. with stable disease on megace* |
| Stable disease | 33 | 21 (64%) |
| Progression | 27 | 3 (11%) |
| | *ER negative* | |
| *Response to first line tamoxifen* | *No. of patients* | *No. with stable disease on megace* |
| Stable disease | 14 | 7 (50%) |
| Progression | 19 | 3 (16%) |

Megace appears useful in patients who have had a previous response to tamoxifen. There is a poor response rate to megace in patients who have previously failed on tamoxifen. The concept of stable disease appears a useful clinical method of identifying hormone sensitive tumours likely to benefit from further hormone therapy.

### Mitozantrone as a single agent in advanced breast cancer

J.F.R. Robertson, M.R. Williams, D.A.L. Morgan & R.W. Blamey

*Department of Surgery, City Hospital, Nottingham, UK.*

The treatment of patients with metastatic breast cancer is palliative rather than curative. In treating patients with chemotherapy, clinicians have to carefully weigh the benefits against the side effects. We have treated 76 patients with single agent mitozantrone with an overall objective response rate at 6 months of 15%. A further 22% had static disease for a minimum period of 6 months. Side effects were acceptable. All 76 patients had received at least one hormonal therapy and 30 patients had previously received ifosfamide. Previous ifosfamide therapy gave no indication whether an individual patient would respond to mitozantrone.

Adriamycin as a single chemotherapeutic agent in the treatment of advanced breast cancer has a reported response rate between 38–50%. The rate of response for mitozantrone ranges from 4–44% with a reported mean of 17% (Leyden *et al., Aust. N.Z. J. Surg.*, **54,** 21, 1984). Our response rate lies at the lower end of this range, although with less side effects than adriamycin at standard dose.

*Enquiries to the ACP Secretariat, Department of Medical Oncology, Christie Hospital and Holt Radium Institute, Manchester M20 9BX, UK.

†For Abstracts, see pp. 223–254 this issue.

‡Reprints of these abstracts are not available – Ed.

### Doxorubicin concentrations in breast tumours, compared with concentrations in normal breast tissue from patients undergoing mastectomy

S. Stallard[1], J.G. Morrison[1], W.D. George[2] & S.B. Kaye[1]

*Departments of [1]Medical Oncology and [2]Surgery, University of Glasgow, UK.*

Chemosensitivity is often assumed to be related to the concentration of drug achieved in tissue, however little is known about the relative sensitivities of breast carcinomas and normal breast tissues to cytotoxic agents. In this study we have compared doxorubicin (DOX) concentrations in paired samples of tumour and normal breast from 21 previously untreated patients, undergoing mastectomy. The relative cellularity of tumour and normal tissue was estimated from the DNA content of both. We have correlated tissue drug levels with serum pharmacokinetic parameters, and also with other known prognostic factors for breast cancer such as ER status and histological grade.

Patients received (25 mg) i.v. DOX, ~1 h before removal of the breast (actual times 60±10 min s.d.) and serum samples were taken at 5, 15, 30, 60 min. Doxorubicin concentrations from serum and tissue were determined by HPLC and DNA content by Burton's method.

There was wide variation in intra-tumoral DOX concentrations (range 220 ng g$^{-1}$ to 1,590 ng g$^{-1}$). Normal tissue also showed a wide variation (range 81 ng to 1,000 ng). In all cases except one, tumour levels were higher than normal tissue levels for a single patient (tumour: normal ratio range 1.27 to 8.30). When DOX was expressed in terms of weight of DNA the tumour and normal drug concentrations were similar (tumour to normal ratio range 1.1 to 1.8). Tissue DOX concentrations (both tumour and normal breast) correlated with peak serum values ($P<0.05$). These findings suggest that, although there is interpatient variation in tissue levels, drug concentrations in tumour and normal breast are similar in a single patient.

### Chemotherapy of advanced breast carcinoma in Africans

R.O. Senbanjo, F.N. Ihekwaba & O.O. Ajayi

*Department of Surgery, University College Hospital, Ibadan, Nigeria, UK.*

Nigeria and indeed Africa remains today the greatest repository of advanced breast cancer. Some cancer patients from Africa seek treatment in Europe and America where chemotherapy is given using Caucasian standards. Fifty-four Nigerians with advanced breast cancer were treated with standard schedule of cyclophosphamide, methotrexate and 5-fluoro uracil (CMF) on out-patient basis. The dose of all chemotherapy drugs was reduced in any subsequent cycle by 30% with moderate toxicity (WBC <3,000 mm$^{-3}$; platelet <100,000 mm$^{-3}$) or 50% with severe toxicity (WBC <2,000 mm$^{-3}$; platelet <75,000 mm$^{-3}$). The mean total dose received was calculated as a percentage of the standard dose for each patient over a 6 month period.

The patients, aged between 25 and 69 years (mean 47 years) had inoperable Stage III disease (26 patients), Stage IV disease (10 patients) or recurrent/metastatic disease after mastectomy (18 patients). Only 2 patients (4%) were able to receive over 85% of the calculated standard dose, while 43 (80%) received less than 65% of the calculated standard dose. The maximum tolerance dose in Nigerians is much lower than in their Caucasian counterparts although comparable figures were obtained for objective response rate (47%); median duration of response (7 months) and median survival (9 months). Leukopenia (WBC<2,500) occurred in 48% during the first course and in subsequent courses in an additional 21% of patients. Three patients (6%) died of chemotherapy related causes all during the first course. The normal leucocyte count is lower in Africans and malnutrition, infections and infestations are rampant. The role of these factors in the poor tolerance of chemotherapy in Africans requires further investigations.

### A direct radioimmunoassay (RIA) for 4-hydroxyandrostenedione measurement in plasma

J. Khubieh, W. Aherne & J. Chakraborty

*Biochemistry Department, University of Surrey, Guildford GU2 5XH, Surrey, UK.*

4-Hydroxyandrostenedione (4-OHA) is a potent and specific inhibitor of aromatase which mediates the conversion of androgens to oestrogens, and has been successfully used for the treatment of breast cancer. Plasma levels of 4-OHA (0.7–23.2 ng ml$^{-1}$) have been measured in a Phase II study (Goss *et al., Cancer Res.*, **46**, 4823, 1986) by adapting an RIA for androstenedione which cross-reacts by 25% with 4-OHA. We report here the characteristics of an RIA using a specific antiserum to 4-OHA, and a tritiated label (15.6 Ci mmol$^{-1}$) for direct plasma assay at the low concentrations expected. The standard curve ranges from 50 ng ml$^{-1}$ to 5 ng ml$^{-1}$ with a lower limit of detection of 0.25 ng ml$^{-1}$. Inter-assay variation of a pooled patient sample measurement was 6% (37.6 ng ml$^{-1}$ ±2.32). Complete recovery of 4-OHA added to normal female plasma was obtained at concentrations of 1 ng ml$^{-1}$ to 2 ng ml$^{-1}$. Analysis of patient samples and normal plasma enriched with 4-OHA showed that up to 87% of plasma 4-OHA was bound to proteins.

The antiserum cross-reacts with androstenedione, testosterone, 3β-hydroxy-5α androstan-4,16 dione, 4-hydroxy-oestrone, 4-hydroxy-oestradiol and cortisol by 0.9, 2.4, 0.6, 0.3, 0.5 and 0.3% respectively. RIA of 4-OHA in plasma samples carried out in conjunction with solvent extraction and Lipidex 5000 chromatography confirmed that there was no interference from androstenedione. However, 4-hydroxy-testosterone (4-OHT), a putative metabolite of 4-OHA, cross-reacts by 100%. The RIA, will not only be useful for pharmacokinetic studies of 4-OHA, but, combined with Lipidex 5000, will provide a means of determining the extent and time course of metabolism to 4-OHT.

### HMFG2 expression in primary ovarian cancer, relationship to tumour differentiation and survival

S.G. Allan, F.G. Hay, L.W. Adams & R.C.F. Leonard

*Imperial Cancer Research Fund Medical Oncology Unit, Department of Clinical Oncology, Western General Hospital, Edinburgh, UK.*

The HMFG2 antigen is variably expressed and may be of prognostic significance in cancers of the lung and breast. It is also expressed variably on the cell surface of ovarian cancer primary tumours and is shed into the serum, a potential target for disease monitoring and therapy. Careful evaluation of the immunoreactivity among the histological subtypes of ovarian cancer is thus important and may provide prognostic information. We have looked at HMFG2 expression in a variety of formalin-fixed primary tumours of ovarian epithelial origin and related antigen expression to differentiation and to survival. Using a standard PAP technique tissue sections were assessed and scored by two observers for extent of staining, 0, <50% and >50% staining.

| | Serous | | Mucinous | | Endometrioid | | |
|---|---|---|---|---|---|---|---|
| | WD[a] | PD[b] | WD[a] | PD[b] | WD[a] | PD[b] | Clear cell |
| 0 | 1 | 0 | 2 | 0 | 0 | 0 | 0 |
| <50 | 1 | 3 | 3 | 0 | 3 | 1 | 4 |
| >50 | 3 | 10 | 4 | 1 | 2 | 0 | 0 |

[a]WD = Well differentiated. [b]PD = Poorly differentiated.

The majority of ovarian cancer primaries exhibit HMFG2 but unlike Ward *et al*., (*Cancer*, **60**, 787, 1987) the series shows that poorly differentiated serous carcinomas exhibits marked expression of HMFG2 in contrast to the other serous types. Other histopathological groups do not show such a consistent pattern. Within a single specimen heterogeneity of staining was seen. Regardless of therapy, the survival of patients who express HMFG2 strongly (>50%) was worse than those who expressed it poorly (<50%) respective survival times being 11 months and 16 months ($P=0.01$, Mann–Whitney U). Further data accrual will be required to verify this finding.

### Oestrogen and progesterone receptors in ovarian cancer

M. Harding[1], S. Cowan[2], L. Cassidy[3], H. Kitchener[3] & R. Leake[2]

*Departments of [1]Medical Oncology, [2]Biochemistry and [3]Gynaecology, University of Glasgow, UK.*

The prognostic significance of steroid receptors in breast cancer is well recognised. To determine whether expression of receptors is a biological marker of less aggressive disease in gynaecological cancer, quantitation of oestrogen (ER) and progesterone (PR) receptors has been carried out over the last 4 years. Soluble and nuclear receptor levels were determined by Scatchard analysis: tissue samples were defined as positive if soluble ER and/or PR levels exceeded 10 fmol mg$^{-1}$ protein with nuclear levels >150 fmol mg$^{-1}$ DNA.

The majority of benign ovarian cysts (14/15) and borderline malignant tumours (2/2) were ER− PR−: both (2/2) granulosa cell tumours were ER+ PR+. Of the 66 samples from patients with non-pretreated epithelial ovarian cancer, 18 were ER+ PR+ (27%), 15 were ER+ PR−, 5 were ER− PR+ and 28 were ER− PR− (42%); approximately 10% of each subgroup were premenopausal. FIGO Stage I and II tumours were more commonly PR+ (10/23) than PR- (4/43) but there was no association between early stage and ER status (6/33 ER+ vs. 8/33 ER−).

Survival of patients with advanced disease (FIGO Stages III or IV) indicates that expression of steroid receptors may be of prognostic significance though data are preliminary. Median survival (in months) according to receptor status was:

| | Receptor + | Receptor − |
|---|---|---|
| ER | 12 | 6.5 |
| PR | >24 | 8 |
| ER+PR | >12 | 5 |

### The effect of LHRH agonist, zoladex, on ovarian histology

J.F.R. Robertson, K. Williamson, I.O. Ellis, R.I. Nicholson & R.W. Blamey

*Department of Surgery, City Hospital, Nottingham, UK.*

In premenopausal women with advanced breast cancer the luteinising hormone releasing hormone (LHRH) zoladex produces response rates similar to surgical oophorectomy. There have been proposals both to use zoladex as adjuvant therapy and in benign disease: irreversible ovarian suppression would be an unwanted side effect in these patients. We have looked at the histological changes in the ovaries of patients with advanced breast cancer treated by zoladex.

In a phase 1 clinical trial of zoladex as first line hormonal therapy in premenopausal women with advanced breast cancer, patients on progression of their disease underwent surgical oophorectomy. The histology of 39 ovaries from 23 women treated with zoladex (Zx) has been compared to 68 ovaries from 34 patients who underwent surgical oophorectomy as primary therapy (Ox).

Histologically both the Zx and Ox groups show similar follicular phase development. Follicular cysts were seen more often in the ovaries of Zx treated patients than in the Ox patients ($P<0.05$). Corpus lutea were seen more often in the ovaries of Ox patients than in the Zx patients ($P<0.01$).

Zoladex appears to arrest development of follicles with formation of follicular cysts. Despite low FSH levels folliculogenesis was not inhibited.

### The use of verapamil to overcome drug resistance in myeloma

M.E. Gore, P.J. Selby, B. Millar, J. Maitland & T.J. McElwain

*Institute of Cancer Research and Royal Marsden Hospital, Downs Road, Sutton, Surrey, UK.*

There is considerable experimental evidence that verapamil can overcome adriamycin resistance in a variety of experimental tumour models. Following a suggestion made by Durie and colleagues that this phenomenon might be applied to patients with myeloma, we have conducted a study on 7 myeloma patients with acquired or primary resistance to the VAMP regimen (vincristine 0.4 mg daily, adriamycin 9 mg m$^{-2}$ both by continuous infusion for 4 days, methylprednisolone 1 gm$^{-2}$ IV orally daily for 5 days repeated every 3 weeks). Four patients were treated with VAMP to a plateau phase i.e. after achieving the maximum reduction in paraprotein no further response to VAMP was seen. Three patients had primary VAMP-resistant disease. These 7 patients were all treated with verapamil (10 mg day$^{-1}$ by continous infusion for 5 days) together with further treatments with the VAMP regimen. In 4 out of 7 patients the treatment produced further reductions in the paraprotein levels of >25%. In 2 of these patients, the paraprotein fell by >50% and in one case the paraprotein became unmeasurable. Two of the responders to initial observations support the hypothesis that verapamil can overcome some of the mechanisms of resistance to adriamycin and vincristine-containing regimens in patients suffering from myeloma. Further studies to elucidate its precise role in the management of myeloma patients treated on this and similar schedules are indicated.

**Phase I study of recombinant DNA granulocyte-macrophage colony stimulating factor (rGM–CSF)**

W.P. Steward[1], R. Austin[2], J.H. Scarffe[1], D. Crowther[1], N. Thatcher[1], G. Morgenstern[2] & P. Loynds[3]

[1]*CRC Department of Medical Oncology,* [2]*Department of Haematology, Christie Hospital, Wilmslow Road, Manchester M20 9BX;* [3]*Schering Corporation, Herts, UK.*

Recombinant DNA granulocyte-macrophage colony stimulating factor (rGM–CSF) (Schering Corporation, New Jersey) was administered as i.v. infusions over 30 min to patients (pts) with advanced progressive neoplasms. The schedule was as follows: 10 days of daily infusions, 10 days without GM–CSF, 10 days of daily infusions and finally 20 days of alternate day infusions. Regular routine haematological and biochemical investigations were performed and blood was taken to assess pharmacokinetics and neutralising antibodies to GM–CSF. Bone marrows were taken during treatment to obtain GM–CFC and mixed population assays. The study design was for 3 pts to be entered at each of the following dose levels: 0.3, 1, 3, 10, 30, 60 $\mu g\, kg^{-1}$ body weight. The study endpoint was a maximum tolerated dose (MTD) resulting in a WBC $>50,000\, mm^{-3}$ or platelets $>600,000$ in 66% of pts (6 pts to be entered at the MTD). Fifteen pts have entered this study to date, 3 at each dose level to 30 $\mu g\, kg^{-1}$. No significant alteration of any haematological parameters was seen with 0.3 or 1 $\mu g\, kg^{-1}$. The mean total WBC count ($\times 10^9\, l^{-1}$) over the first 10 days of GM–CSF rose from 11 to 14 at 3 $\mu g\, kg^{-1}$, 8 to 23 at 10 $\mu g\, kg^{-1}$ and 7 to 27 at 30 $\mu g\, kg^{-1}$. No significant difference in the granulocyte:monocyte ratio occurred, but an eosinophil was seen (up to 12% total WBC count) at dose levels above 3 $\mu g\, kg^{-1}$. The magnitude of the rise in WBC count appears to be greater during the second phase of daily infusions. There was a rapid fall of WBC count to pretreatment levels after the cessation of GM–CSF (mean 48 h). Side effects included transient pyrexia after the first two infusions of GM–CSF and bone pains (predominantly lower vertebrae) which were severe and required analgesia in 2 patients receiving 30 $\mu g\, kg^{-1}$ and one receiving 10 $\mu g\, kg^{-1}$. Further patients are being entered at a dose of 60 $\mu g\, kg^{-1}$ and this is anticipated to be the MTD. No antibodies to GM–CSF have been detected in 6 patients tested to date.

**Phase I study of the anthrapyrazole CI-941 with pharmacokinetically guided dose escalation**

M.A. Graham, B.J. Foster, D.R. Newell, L.A. Gumbrell & A.H. Calvert

*Drug Development Section, Institute of Cancer Research, Sutton, Surrey, UK.*

CI-941 is a novel DNA binding drug which displays equivalent or superior antitumor activity to doxorubicin in experimental systems yet does not undergo metabolic reduction *in vitro* and hence is unlikely to cause free radical mediated tissue damage *in vivo*. The MTD and $LD_{50}$ of CI-941 in mice (i.v. bolus dose) were 45 and 60 $mg\, m^{-2}$, respectively, with a plasma concentration *vs.* time AUC at the MTD of 110 $\mu M \cdot min$. The pharmacokinetics of CI-941 were linear in mice over the dose range 4.5–45 $mg\, m^{-2}$ and the plasma protein binding in human and mouse plasma was similar (80–90%). The dose limiting toxicity in mice was leukopenia. In a phase I study of CI-941 given as an i.v. bolus dose once every 3 weeks 14 patients have received a total of 23 evaluable courses. The starting dose was 5 $mg\, m^{-2}$ with a target plasma AUC of 110 $\mu M \cdot min$. Wide variation in AUC at 5 $mg\, m^{-2}$ (23, 17, 54 $\mu M \cdot min$) cautioned against rapid escalation and hence 5 $mg\, m^{-2}$ dose increments were employed up to 30 $mg\, m^{-2}$. Pharmacokinetic monitoring has been completed on 10 patients following 14 courses at doses up to 25 $mg\, m^{-2}$. CI-941 plasma clearance is triphasic with $t_{1/2}$ values of $7.5 \pm 1.8$ min, $69 \pm 30$ min and $19 \pm 7$ h. The mean total plasma clearance of CI-941 was rapid (436 $ml\, min^{-1}\, m^{-2}$), however, over this dose range considerable variation was found (coefficient variation 47%). Variation in CI-941 clearance was not explained by pretreatment renal, hepatic, or cardiac function. In 4 patients who received 2 courses of CI-941 the interpatient variation in clearance was larger than the intrapatient variation. At 25 $mg\, m^{-2}$ the AUC was 81, 89 and 129 $\mu M \cdot min$ indicating that the target AUC should be achieved at 30 $mg\, m^{-2}$. In agreement with this prediction leukopenia has been observed: 25 $mg\, m^{-2}$ 2/6 WHO grade 1, 4/6 grade 2; 30 $mg\, m^{-2}$ 1/1 grade 1. Other toxicities at 25 $mg\, m^{-2}$ include nausea and vomiting; 3/6 grade 1, 1/6 grade 2, 1/6 grade 3 and mucositis; 1/6 grade 1, 3/6 grade 2. This study has shown that although pharmacokinetics were of value in guiding dose escalation the interpatient variability found with CI-941 precluded the use of dose increments dictated solely by pharmacokinetics.

**Chronopharmacology of carboplatin**

D.J. Kerr, C. Lewis, B.O'Neil, N. Lawson, R. Blackie, E.M. Rankin & S.B. Kaye

*Department of Medical Oncology, University of Glasgow, UK.*

Depending on the time of administration, a fixed dose of drug may be toxic or therapeutic. Circadian (about daily) stage dependence has been demonstrated in animal models for a number of commonly used cytotoxic agents and there is increasing clinical awareness of the importance of timing drug delivery. We treated 8 patients with advanced ovarian cancer (stages II and IV) with carboplatin (400 $mg\, m^{-2}$) at 0600 or 1800 on successive courses, in random order. Plasma ultrafiltrate and urinary carboplatin concentrations were measured using a sensitive and specific HPLC assay and full blood count was estimated weekly.

| *Time* | *Peak plasma core* ($\mu g\, ml^{-1}$) | *AUC* ($\mu g\, ml^{-1}\, h$) | *Urinary excretion* ($mg\, 24\, h^{-1}$) |
|---|---|---|---|
| 0600 | $42.3 \pm 21$ | $92 \pm 22$ | $174 \pm 36$ |
| 1800 | $47.3 \pm 16$ | $113 \pm 28$ | $58 \pm 28$ |

| *Time* | *WBC* | *Platelets* |
|---|---|---|
| 0600 | $3.0 \pm 1.0$ | $95,000 \pm 23,000$ |
| 1800 | $3.7 \pm 0.9$ | $180,000 \pm 56,000$ |

It is clear from this data that the time of administration of carboplatin has a significant effect on the pharmacokinetics of the drug and the ensuing degree of myelosuppression.

**Predictors of etoposide pharmacokinetics in man**

P.I. Clark, S.P. Joel, S. Houston, W.M. Gregory & M.L. Slevin

*ICRF Department of Medical Oncology, St Bartholomew's and Homerton Hospitals, London, UK.*

Pharmacokinetics (PK) of 101 patients consecutively treated with single-agent etoposide (50–5000 $mg\, m^{-2}$) were analysed for factors predictive of etoposide PK. The areas under the plasma concentration versus time curve (AUC) have been standardised to a dose of 100 mg m$^{-2}$. Mean values $\pm$s.d. of elimination half-life ($t_{1/2}$) were 6.5$\pm$1.9 h, AUC 105$\pm$31 $\mu$g ml$^{-1}$ h$^{-1}$, volume of distribution (Vd) 9.4$\pm$3.0 l m$^{-2}$ and plasma clearance (Cl) 17.2$\pm$5.5 ml min$^{-1}$ m$^{-2}$. Univariate analysis showed significant correlations between AUC and both plasma creatinine (P) ($r$=0.50, $P$<0.001) and log$P$ ($r$=0.50, $P$<0.001). Multivariate linear regression analysis showed no further significant correlations of either AUC or Cl with age, albumin, bilirubin, alkaline phosphatase (ALP), aspartate transaminase or gamma-glutamyl transferase (GGT). Patients with renal and hepatic impairment were examined separately and compared to those with normal function. Eighty-three patients had $P$<130 $\mu$mol l$^{-1}$, ALP<200 IU l$^{-1}$ and GGT<100 IU l$^{-1}$, and for these, mean $\pm$s.d. were $t_{1/2}$ 6.5$\pm$1.6, AUC 104$\pm$25, Vd 9.4$\pm$3.1 and Cl 17.0$\pm$4.4. Seven patients had renal impairment with $P$>130 $\mu$mol l$^{-1}$ and had significantly altered $t_{1/2}$ 9.2$\pm$2.9 ($P$=0.004, Mann–Whitney), AUC 163$\pm$36 ($P$=0.0002), Cl 10.7$\pm$2.4 ($P$=0.0002), Cl 10.7$\pm$2.4 ($P$=0.0002) but not Vd 8.1$\pm$1.1 ($P$=0.25). Eleven patients had normal renal function but either ALP>200 or GGT>100 or both, and had significantly changed $t_{1/2}$ 4.8$\pm$1.6 ($P$=0.004), AUC 79$\pm$22 ($P$=0.004), Cl 12.1$\pm$8.3 ($P$=0.004) but not Vd 9.8$\pm$2.8- ($P$=0.25). In one patient with obstructive jaundice, PK returned to normal after resolution of porta hepatis lymphadenopathy. Etoposide may need dosage reduction in renal impairment. Further studies of PK and drug metabolism are necessary in patients with hepatic dysfunction before recommending increasing dosage in such patients.

### Sclerosis of lytic bone metastases from breast cancer in response to 3-aminohydroxypropylidene-1,1-biphosphonate (APD)

A.R. Morton[1], G.V. Pillai[2], J.A. Cantrill[2], D.C. Anderson[3] & A. Howell[1]

[1]*CRC Department of Medical Oncology, Christie Hospital, Manchester; and University Departments of* [2]*Pharmacy and* [3]*Medicine, Hope Hospital, Salford, UK.*

The affect of APD was assessed in 16 patients with advanced endocrine unresponsive breast cancer metastatic to bone. 30 mg of APD was given as a 2 h infusion weekly for 4 weeks and 2 weekly for 6 months or until disease progression. Pain was assessed using a linear analogue scale. The effect of APD on bone tumour was measured by urinary calcium/creatinine (Ca/Cr) ratio and by hydroxyproline/creatinine (OHPr/Cr) ratios and serum oesteocalcin. The effect on the tumour was estimated by serial radiographs and monthly measurements of CEA and Cal 5.3. Radiological evidence of sclerosis of lytic metastases was seen in 4 patients and the disease remained stable for the 6 months of the study in a further 4 patients. Disease progressed within 6 months in 8 patients. Pain was significantly decreased by treatment ($P$<0.01). There was a significant decline of Ca/Cr ratio in all patients ($P$<0.001); OHPr/Cr and osteocalcin remained stable. CEA and Ca 15.3 fell in parallel in 3 patients (2PR, 1 stable). No significant side-effects of treatment were seen. We conclude that APD has apparent 'antitumour' activity and gives partial relief of bone pain. Further studies of APD in combination with standard therapy are warranted.

### Mitomycin, ifosfamide and *cis*platin in non-small cell lung cancer

M.H. Cullen[1], A.D. Chetiyawardana[1], R. Joshi[2] & C.M. Woodroffe[1]

[1]*Queen Elizabeth Hospital, Birmingham; and* [2]*Manor Hospital, Walsall, UK.*

Mitomycin, ifosfamide and *cis*platin are three of the most active single agents in the chemotherapy of non-small cell lung cancer. We have combined them in a 24 h schedule ('MIC') for a phase 2 study in patients with inoperable non-small cell lung cancer. The regime consists of: mitomycin 6 mg m$^{-2}$ i.v. bolus, ifosfamide 3 g m$^{-2}$ i.v. infusion over 3 h and *cis*platin 50 mg m$^{-2}$ i.v. infusion over 1 h. Mesna is given with the ifosfamide infusion at a dose of 1 g m$^{-2}$ and a further 500 mg m$^{-2}$ bolus is given 3 h later followed by a 6 h infusion (500 mg m$^{-2}$). The anti-emetic regime consists of lorazepam 2 mg m$^{-2}$ dexamethasone 8 mg m$^{-2}$ and metoclopramide 1 mg kg$^{-1}$ i.v. 30 min prior to chemotherapy, metoclopramide 9 mg kg$^{-1}$ over 24 h and further doses of dexamethasone 4 mg m$^{-2}$ i.v. 4 hourly. Fifty-four ambulatory (WHO performance status 0, 1 or 2) patients with inoperable limited (LD) or extensive stage (ED) disease have entered this study, and 44 are evaluable for response. Eighteen patients have achieved partial remission (41%) and 6 have achieved complete remission (14%) as assessed radiologically. The overall response rate is thus 55%. There have been 19/28 responses in LD (68%) and 5/16 in ED (31%). Sixteen patients have experienced an improvement in WHO performance status rating. Although well tolerated in the majority of patients, the principal toxicity has been vomiting which was severe (WHO 3/4) in 9 patients. There has been one treatment related death. MIC is clearly among the most active combinations in non-small cell lung cancer and will now be tested in a randomized trial against no chemotherapy.

### Transforming growth factor alpha (TGFα) and epidermal growth factor (EGF) in tumour and urine of breast cancer patients

A. Howell[1], H. Anderson[1], H. Gregory[2], C. Thomas[2], I. Willshire[2] & J. Young[2]

[1]*Department of Medical Oncology, Christie Hospital, Manchester; and* [2]*ICI Pharmaceuticals Division, Alderley Park, Macclesfield, UK.*

Extracts of breast tumours ($n$=15) and of urine of breast cancer patients and age match controls were subjected to separation by high performance liquid chromotography. Fractions were evaluated by mitogenic activity using 3T3 cells, colony formation using NRK cells and by specific immunoassays for EGF and TGFα. The system was calibrated using pure biosynthetic growth factors. Concentrations of TGFα were similar in 5 oestrogen and progesterone receptor positive tumours and 5 receptor negative tumours. In 5 tumours removed from patients on tamoxifen TGFα levels were greatly reduced compared with the other two groups. No EGF was detectable in tumours. Both TGFα and EGF were present in similar concentrations in the urine of breast cancer patients and controls. We conclude that tumours do not produce EGF, but produce TGFα which is suppressable by tamoxifen. Similar concentrations of both growth factors in patients and controls suggest that the major source of TGFα is from sites other than tumours.

**Enzyme activity in concentration of serum placental alkaline, phosphatase (PLAP) in monitoring patients with ovarian cancer**

A. Bowman[1], J. Fisken[2], J.E. Roulston[2] & R.C.F. Leonard[1]

[1]*University Department of Clinical Oncology, Western General Hospital, Edinburgh; and* [2]*University Department of Clinical Chemistry, Edinburgh, UK.*

One hundred and fourteen patients with ovarian carcinoma (FIGO stage I (12), II (10), III (69), IV (22) at presentation) had 424 serum samples assayed for the presence of PLAP using the monoclonal antibody $H17E_2$ in a novel immunoradiometric assay which measures simultaneously PLAP activity (A) and concentration (C). Normal ranges were defined on 387 blood donor samples with known smoking history and patients' borderline results disregarded. Correlating serum PLAP with surgical, clinial and radiological disease status, overall sensitivity of PLAP A was 61.7% and C 35.3%, specificity of A was 53.7% and C 74.6% and accuracy of A was 60% and C 49%. Serial levels correlated with clinical disease course in less than 50% of patients. In 21 patients with pathological results available from second-look laparotomy, false positive results for both A and C were noted.

| | *CA 125* | | *PLAP A* | | *PLAP C* | |
|---|---|---|---|---|---|---|
| | *+ve* | *−ve* | *+ve* | *−ve* | *+ve* | *−ve* |
| Pathological CR | 0 | 11 | 8 | 3 | 2 | 6 |
| Pathological PR | 4 | 6 | 5 | 5 | 2 | 6 |

This rigorous clinical test illustrates the severe limitations on the value of PLAP as the sole marker of disease activity in carcinoma of the ovary.

**c-*myc* oncogene expression and cell growth parameters in the evolution of colorectal carcinoma**

P. Johnston[1], M. O'Brien[1], P. Dervan[1], B. Guillick[2] & D.N. Carney[1]

[1]*Mater Hospital, Dublin 7, Ireland; and* [2]*Institute of Cancer Research, London, UK.*

Using an immunohistochemical technique and radiolabelled oncogene probe hybridization we have evaluated the expression of c-*myc* oncogene and Ki-67 nuclear antigen in colorectal carcinomas, adenomas and normal colonic epithelium. Results were correlated with histological grade and Dukes' stage. Cytoplasmic expression of c-*myc* was detected in 40/44 carcinomas, 25/28 adenomas and in the crypts of all 10 normal colonic mucosas. In the normal mucosas c-*myc* expression was noted throughout the intestinal tract with staining intensity similar in both the surface epithelial cells and the basal areas of the crypts. Heterogeneity in cytoplasmic staining was present in many adenomas and carcinomas. c-*myc* gene amplification was detected in only 1 of 20 tumours analysed and in no adenomas. No correlation was noted between c-*myc* expression, histological grade or Dukes' stage. Ki-67 nuclear expression was found in all specimens examined. The percentage of Ki-67 positive nuclei varied among the specimen type, 19–42% in proliferative zones of normal crypts, 20–67% in adenomas and 20–87% in adenocarcinomas. Ki-67 expression was confined to the proliferative zone of the crypt while in carcinomas and adenomas diffuse staining was noted. No correlation was noted between histological grade and Dukes' stage. These data suggest that Ki-67 antigen expression correlates with disease evolution of colorectal adenomas and carcinomas. However no correlation was noted between c-*myc* expression and the evolution from normal mucosa to adenoma and adenocarcinoma.

**Molecular studies of multiple endocrine neoplasia, Type 2a (MEN 2a) and clinical implications**

B.A.J. Ponder[1], C.G.P. Mathew[1], D.F. Easton[2], K. Chin[1], B. Smith[1], M.A. Ponder[1], H. Telenius[1], K. Nakamura[3], T. Cummings[4], H. Sobol[5] and G. Lenoir[5]

*Sections of* [1]*Human Cancer Genetics and* [2]*Epidemiology, Institute Cancer Research, Sutton, UK;* [3]*Howard Hughes Institute, Salt Lake City;* [4]*Tufts New England Medical Center, Boston, USA; and* [5]*IARC, Lyon, France.*

MEN 2a is an autosomal dominant inherited cancer syndrome comprising medullary thyroid carcinoma (MTC: arises from thyroid 'C' cells) and phaeochromotocytomas. Family members can be screened by regular measurement of calcitonin after a provocative stimulus, and by measurement of catecholamines. A genetic marker would eliminate from screening the 50% of family members not at risk, and would be the first step towards elucidating the gene defect and its mechanism. We have mapped the MEN 2a locus to chromosome 10 by genetic linkage using DNA polymorphisms (Mathew *et al.*, *Nature*, **328**, 527, 1987). Currently we have identified 2 DNA markers which are close to the MEN 2a locus, IRBP/RBP3 (LOD score $Z$ 12.78, $\theta=0.03$, 95% upper CL 0.09) and MCT 50 ($\hat{Z}$ 7.10, $\theta=0.03$). Each of 13 informative families tested shows evidence of linkage, but no data are yet available for families with MTC only, nor MEN 2b. The progress of this work and the potential clinical use of current markers will be presented.

Analysis of MEN 2 tumours with chromosome 10 probes has so far shown no allele loss in 16 informative cases. It is, however, premature to conclude that the double recessive mechanism described for retinoblastoma and other inherited tumours does not also operate in MEN 2.

**Inherited predisposition to breast cancer**

B.A.J. Ponder[1], D.F. Easton[2], K. Anderson[2] & J. Peto[2]

*Sections of* [1]*Human Cancer Genetics and* [2]*Epidemiology, Institute of Cancer Research, Sutton, Surrey SM2 5PX, UK.*

There is no proof that familial clustering of breast cancer has an inherited rather than an environmental basis. Proof will only come from the identification of a genetic marker which segregates with breast cancer in the families. Such a marker would improve the recognition of individuals at risk. It would also be a first step to unravelling the mechanism of predisposition, which might lead to the identification of the important carcinogens and to rational efforts at prevention or treatment. The search for the gene presents problems. One approach is to identify pairs of affected siblings with premenopausal disease and to obtain blood for linkage studies using DNA markers. On the basis of random segregation, sisters should share both alleles at any locus by inheritance from their parents with a probability of 1:4, neither allele 1:4, and one or other allele 2:4. The presence of a 'breast cancer gene' will distort this random inheritance pattern for a DNA marker close to the disease locus. The advantage of this approach over 'classical' linkage is that it avoids the need to specify (1) a dominant or recessive model, or (2) the penetrance, and (3) it can accommodate the problem of phenocopies. Our analysis of a large population-based cohort shows that the risk of ca. breast in sisters themselves under 50 years, of cases diagnosed below 50 years, is increased 3.5-fold. If this increased risk were due to a single dominant gene, with 150–200 highly polymorphic DNA markers at 20 cM intervals over the genome, this gene could be detected by linkage using 80 sib pairs, while a recessive gene would require at most 30 pairs. If the effect were due to two dominant genes, in the worst case up to 200 pairs may be required.

### Ototoxicity of *cis*-platinum

R. Skinner[1], A.D.J. Pearson[1], H.A. Amineddine[1], D.B. Mathias[1] & A.W. Craft[1]

[1]*Department of Child Health, University of Newcastle upon Tyne; and* [2]*Ear, Nose and Throat Department, Freeman Hospital, Newcastle upon Tyne, UK.*

*Cis*-platinum is an important drug in the treatment of childhood malignancy. However, it has a number of adverse effects, one of which is ototoxicity. To determine the extent and reversibility of hearing damage in children treated with *cis*-platinum, 22 patients had serial audiograms. Their ages at start of treatment ranged from 7 to 19 years, with a mean of 13 years and they received a median cumulative dose of *cis*-platinum of 543 $mg\,m^{-2}$ (range 312 to 1072 $mg\,m^{-2}$) for oesteogenic sarcoma (11), primitive neuroectodermal tumour (5), rhabdomyosarcoma (3), neuroblastoma (2) and dysgerminoma (1).

Maximum hearing loss was greater at high frequencies with a median loss of 12.5 decibels (dB) (range 2.5–32.5) at 1,000 Hz, 10.0 dB (range 0–62.5 dB) at 2,000 Hz, 31.25 dB (range 7.5–95 dB) at 4,000 Hz, and 70.0 dB (range 17.5–95 dB) at 8,000 Hz ($P<0.0001$). There was no significant difference in the severity of hearing loss between right and left ears.

There was no significant correlation between the severity of hearing loss and the number of days of other ototoxic treatment, the total dose and the number of courses of *cis*-platinum, and the age at the start of treatment. There was no significant difference in hearing loss between children receiving high dose (200 $mg\,m^{-2}$) and those receiving conventional dose (75–125 $mg\,m^{-2}$) *cis*-platinum.

Five of 7 children who had follow-up audiometry continued for more than a year after the end of treatment showed some reversibility of hearing loss (defined as an improvement of 15 dB or greater) in at least one ear.

This study demonstrates that ototoxicity is a significant adverse effect of *cis*-platinum, but further follow-up is needed to determine how much recovery may occur. No increase in toxicity occurred with use of high dose *cis*-platinum.

### Persistent renal damage after treatment with *cis*-platinum for childhood malignancies

A.L. Brunetto[1], A.D.J. Pearson[1], L. Price[1], H.A. Aminnedine[1], W. Sheldon[1] & A.W. Craft[1]

*Departments of* [1]*Child Health and* [2]*Clinical Biochemistry, University of Newcastle upon Tyne, UK.*

It is recognised that *cis*-platinum may cause acute renal damage with a reduction in the glomerular filtration rate as well as renal tubular dysfunction. As there is now an increasing number of survivors of childhood cancer it is important to determine the late effects of therapy. Therefore a group of children who had completed therapy, which included *cis*-platinum, have been studied to determine the extent of renal damage which persists after cessation of therapy.

Twenty-one children (12 males) who had received *cis*-platinum between 0.3 and 5.6 years previously for oesteogenic sarcoma (12), rhabdomyosarcoma (13), yolk sac tumour (3), neuroblastoma (2) and primitive neuroectodermal tumour had their tubular function investigated and glomerular filtration rate (GFR) measured. GFR was determined by the disappearance from the plasma of $^{51}Cr$ EDTA.

Fifteen patients had a serum magnesium (Mg) concentration below the reference range. In these patients the urinary excretion of Mg was inappropriately high as reflected by a reduced tubular absorption of Mg. Compared to control children these children had a significantly lower Mg concentration ($P=0.014$), lower tubular reabsorption of Mg ($P=0.09$) and lower tubular reabsorption of phosphate ($P=0.06$). No other abnormalities of renal tubular function were detected. The median GFR for the group was 89 with a range of 44–125 $ml\,min^{-1}$ 1.73 $m^{-2}$. The serum Mg concentration, an index of tubular damage, was related to the child's age, time since completion of chemotherapy, sex, disease, and amount of gentamycin that the child had been prescribed. Those patients who had received more gentamycin tended to have a lower serum Mg concentration ($P=0.07$). However, there was no relationship between time since completion of therapy with serum Mg concentration. This study suggests that renal damage persists in the survivors of childhood cancer treated with *cis*-platinum. This dysfunction is manifested by low serum Mg concentrations and a reduction of GFR. Early results of follow up studies suggest that hypomagnesaemia may persist in some children while GFR may recover.

### Gut damage during the treatment of childhood acute lymphoblastic leukaemia

A.L. Brunetto[1], A.D.J. Pearson[1], L. Price[1], M.F. Laker[2] & A.W. Craft[1]

*Departments of* [1]*Child Health and* [2]*Clinical Biochemistry, University of Newcastle upon Tyne, UK.*

Gut toxicity in patients receiving treatment for childhood acute lymphoblastic leukaemia (ALL) is assuming greater importance as new intensive strategies are being used. Recent retrospective reviews show that following consolidation therapy of ALL 38% of patients develop gastrointestinal toxicity and 25% of these develop persistent intestinal damage. At present it is impossible to predict prior to therapy which children will develop these complications. In an attempt to identify those children at greatest risk of gut toxicity a cohort of children undergoing intensive therapy for childhood ALL have been studied sequentially. Small intestinal mucosal function has been assessed by a novel intestinal permeability test. Three non-metabolised sugar markers were administered in a oral solution of 696 $mOsm\,kg^{-1}$ containing 4 $mg\,m^{-2}$ mannitol (M), 4 $mg\,m^{-2}$ lactulose (L) and 1.6 $mg\,m^{-2}$ of 3-0-methyl-glucose (MG). The percentage of the amount ingested that was excreted in a 5 h urine collection was measured. M and L passively permeate the small bowel mucosa while MG is actively absorbed. Fifty normal children excreted a mean of 0.29% (s.d. = 0.1) of L, 42.8% (s.d. = 10.7) of MG and 16.3% (s.d. = 4.1) of M.

Children with ALL were studied prior to and weekly on 5 occasions after intensification therapy. Two weeks after chemotherapy M, MG and L absorption was impaired compared to controls and values before consolidation ($P=0.02$ and $P=0.05$). This was followed by gradual recovery which reached normal levels by 5 weeks in the majority of children. However in some children recovery of sugar permeation did not occur. Longitudinal studies revealed that these children developed persistent enteropathy with malabsorption.

Studies are now in progress to identify the clinical and nutritional characteristics of those children who developed gut toxicity. By these means it may be possible to identify those children at greatest risk of gut damage and to develop methods of intervention to prevent toxicity.

### Evaluation of simultaneous four-parameter flow cytometry for the screening of samples from the uterine cervix

D.C. Davies[1], R.L. Adams[1], M. Driver[2] & D.E.H. Tee[1]

*[1]Departments of Immunology and [2]Morbid Anatomy (Cytology), King's College School of Medicine and Dentistry, London SE5 8RX, UK.*

The purpose of this study is to investigate the role of multiparametric flow cytometry in the identification of the normal population in a cervical screening programme. Using samples from normal and at-risk patients we are simultaneously analysing DNA content, cell markers, cell volume and wide angle light scatter.

A fixation regime has been established allowing staining of nuclear DNA by propidium iodide without removal or alteration of the cell surface and without a change in cell volume. A previous immunohistochemical study, using an antibody to the transferrin receptor, showed positive staining with the majority of tumours and severe dysplasias and the complete absence of staining with normal epithelia suggesting that this antibody may distinguish between normal and neoplastic cells (Lloyd *et al.*, *J. Clin. Path.*, **34**, 131, 1984). This antibody is now being used in flow cytometry although other markers are being investigated.

The most effective combination of parameters giving rise to no false negative results is being established and this combination will be used to identify normal specimens. Data from 2,000 cervical samples will be reported comparing flow cytometry with routine cytology.

Application of multiparametric flow analysis as a primary screening procedure will drastically reduce the labour content associated with the examination of normal smears which form at least 70% of the samples processed by most routine cytology laboratories.

### Urinary ifosfamide metabolite profile after oral and intravenous administration

H.L. Roberts[1], M.J. Lind[2], N. Thatcher[2] & J.R. Idle[1]

*[1]Department of Pharmacology, St Mary's Hospital Medical School, London W2 1PG; and [2]Department of Medical Oncology, Christie Hospital, Manchester M20 9BX, UK.*

Quantitation of the metabolites of oxazaphosphorine drugs such as ifosfamide (IF) is important in understanding both the efficacy and toxicity of the drug in individual patients. IF and its principal metabolites were determined by high-performance thin layer chromatography-densitometry (HPTLC-PD) in 0–24 h urines from 18 NSCLC patients who had been given IF (1.5 $g\,m^{-2}$) either orally or intravenously.

On HPTLC-PD, $R_f$ values for the various metabolites were: IF (0.70), the two dechloroethyl metabolites (0.47), carboxyifosfamide (0.33), 2-chloroethylamine (0.10) and isophosphoramide mustard (0.02). At high concentration it was sometimes difficult to separate these last two alkylating metabolites; they were thus estimated conjointly.

Percent dose eliminated (mean $\pm$ s.d.) after i.v. and p.o. administration respectively for each compound was: IF ($1.9 \pm 1.5$, $4.1 \pm 2.1$), dechloroethyl metabolites ($2.1 \pm 1.2$, $6.4 \pm 3.8$), carboxyifosfamide ($0.6 \pm 0.4$, $6.0 \pm 10.0$;), 2-chloroethylamine plus isophosphoramide mustard ($0.2 \pm 0.5$, $5.3 \pm 6.2$), total recovery of metabolites ($2.8 \pm 1.5$, $17.8 \pm 14.0$).

HPTLC-PD presents a novel means of determining the urinary metabolites of ifosfamide. Production and excretion of both the detoxicated and activated metabolites are increased over 6-fold after p.o. compared with i.v. administration.

These data may be interpreted as the result of a significant first-pass metabolism of IF, although this requires further investigation. Since the cytotoxic metabolites are increased over 20-fold after p.o. administration, this has clear implications for the use of oral IF in cancer chemotherapy.

### A new approach to rIL-2 treatment in malignant melanoma – Preliminary analysis of the immunological response in relation to clinical outcome

A.K. Ghosh[1], H. Dazzi[2], N. Thatcher[2] & M. Moore[1]

*[1]Paterson Institute for Cancer Research and [2]Department of Medical Oncology, Christie Hospital, Manchester M20 9BX, UK.*

Interleukin 2 (IL-2) is a T cell growth factor and essential for the induction and growth of lymphokine activated killer (LAK) cells, a phenotypically heterogeneous effector population, derived largely from natural killer (NK) precursors ($CD3^-$ $CD16^+$). Adoptive immunotherapy where LAK cells were given in conjunction with IL-2 has resulted in an increased response rate compared to IL-2 alone but treatment toxicity was high (Rosenberg *et al.*, *N. Engl. J. Med.*, **316**, 899, 1987).

To avoid multiple leukophoresis for *in vitro* generation of LAK cells and to reduce toxicity, recombinant IL2 (rIL-2) was given as a first dose via an intra arterial catheter into the spleen, followed by 4 i.v. doses on alternate days. A total of 3 courses were planned at 3 week intervals. The maximum tolerated single dose was $11 \times 10^6$ Cetus $Um^{-2}$. Thirty patients with progressive metastatic malignant melanoma entered the study; in 4, treatment is still ongoing. Four patients showed a PR, 5 have stable disease.

The PBMC of all patients tested contained LAK precursors estimated by *in vitro* rIL2 induction of cytotoxicity against NK-sensitive (K562) and NK resistant, LAK-sensitive (MEL 1) targets. After *in vivo* administration, the proportion of IL-2 receptor (Tac) positive lymphocytes was elevated 5- to 30-fold and NK activity increased and LAK activity induced in 12/16 and 8/16 patients respectively. However, LAK inducibility *in vivo* did not correlate with clinical response. Provisionally the results suggest that *in situ* events are not necessarily reflected in the periphery, or that IL-2 acts upon other components of the host response.

### DNA ploidy and prognosis in superficial bladder cancer

J.R.W. Masters[1], R.S. Camplejohn[2], M.C. Parkinson[1] & C.R.J. Woodhouse[1]

*[1]Institute of Urology, University College London; [2]Richard Dimbleby Department of Cancer Research, St Thomas's Hospital, UK.*

The majority of superficial bladder cancers recur, and 10–20% progress to muscle invasion. The course of the disease usually is prolonged, but regular (3–12 month interval) cystoscopic review is required, and a reliable predictor of either recurrent superficial or muscle-invasive disease would improve patient management. DNA ploidy was measured by flow cytometry using nuclei extracted from paraffin sections in an unselected series of 136 biopsies from 100 patients presenting in 1978–1979 with a histopathological staging of P1 disease. DNA ploidy was measurable in 107 (79%) of the biopsies, of which 22 (21%) were diploid, 19 (18%) were tetraploid and 66 (62%) were aneuploid. Diploid tumours tended to be histopathologically better-differentiated ($P<0.01$) and have fewer S-phase nuclei ($P<0.001$). Muscle-invasive disease was more likely to develop in patients whose tumours had been recurrent, poorly-differentiated or aneuploid. None of the 16 well-differentiated tumours developed muscle-invasive disease during a 5 year follow-up period, but in every category a minimum of 60% of the cases recurred, at least with superficial disease. Of the 67 cases that were evaluable and had a complete 5 year follow-up, 61% had developed at least one superficial recurrence, 32% had developed more advanced disease (minimum P2) and only 7% had been completely free of disease. The results indicate that DNA ploidy is strongly associated with prognosis, but of less value than histopathological grade in planning patient management, and not useful for distinguishing the prognosis of moderately-differentiated tumours. It is concluded that the routine measurement of DNA ploidy is of limited assistance in this disease.

**Fatty acid saturation index in peripheral blood cell membranes of AIDS patients**

N.A. Habib[1], K. Apostolov[2], W. Barker[2], S.B. Kelly[1], D. Jeffries[3], S.M. Forster[3], R.C.N. Williamson[1] & C.B. Wood[4]

[1]*Bristol Royal Infirmary;* [2,4]*Royal Postgraduate Medical School, London; and* [3]*St Mary's Medical School, London, UK.*

Optimal membrane fluidity is an important factor in cell physiology and is dependent mainly on the degree of fatty acid desaturation of the lipids in the membrane bilayer. Significant changes have been reported in the saturation of fatty acids, predominantly in the ratio of stearic to oleic acid, in erythrocyte cell membranes of cancer patients (Wood *et al., Br. Med. J.*, **291**, 163, 1985). This ratio is referred to as the saturation index (SI).

In this study, the lipid analysis of peripheral blood cells from patients with AIDS ($n=12$) and control subjects ($n=24$) was investigated by gas liquid chromatography.

The SI was significantly lower ($P<0.0001$) in AIDS patients (means: RBC=0.18; WBC=0.11) compared to the controls (means: RBC=1.03; WBC=1.40). This suggests the HIV could be responsible for the low SI seen in peripheral blood cells of AIDS patients. It is also interesting that the index is differentially lower in the WBCs, the target cells of the AIDS virus.

This desaturation of peripheral cell membranes could partly explain the systemic metabolic disturbances seen in this infection.

**Some patients with epithelial ovarian cancer (EOC) relapsing from first line therapy will never respond: Prediction of these patients and implications for phase III studies**

F. Lawton, C. Redman & G. Blackledge

*West Midlands CRC Clinical Trials Unit, Queen Elizabeth Hospital, Birmingham B15 2TH, UK.*

Median survival for patients (pts) with EOC from first relapse or progression is 5–6 months. New drugs are needed in EOC but must be tested in pts who are appropriate to demonstrate activity. Between May 1983 and June 1987, 93 pts with EOC were entered into 4 phase II studies of chemotherapy. The regimens had been chosen on the basis of *in vitro* cytotoxicity data. 35/93 (38%) responses were seen. In an attempt to identify which pts might be most likely to respond, pts were divided into the following groups: (a) pts progressing on first line *cis*-platinum therapy ($n=22$); (b) pts relapsing from first line *cis*-platinum therapy and treated with phase II agents 3–20 months after cessation of first line therapy ($n=40$); (c) pts relapsing 20+ months after primary treatment ($n=17$); and (d) pts receiving phase II agents as third line or greater treatment ($n=14$).

Response rates for the groups were: (a) 1/22 (4.5%); (b) 14/40 (38%); (c) 16/17 (94%); and (d) 4/14 (29%). There was no relationship between the dose of first line *cis*-platinum and response to phase II agents. No other factors appeared to be of relevance in predicting for response on univariate analysis, although pts in group (c) were more likely to be entered into phase II studies once activity had been seen for the agents in other pts. Two responding pts in group (d) had had responses to previous treatments, but the other two responders to phase II agents had been non-responsive to earlier treatments.

These data suggest that pts progressing on first line treatment are unlikely to respond to phase II agents even if activity is seen *in vitro* and in other pt groups. Patients receiving phase II agents as third line or greater treatment are also unlikely to respond. These observations should be borne in mind when writing inclusion criteria for protocols of phase II agents, to ensure that active compounds are not missed in a disease where new treatment approaches are required.

**Comparison of toxicity of mitozantrone and doxorubicin when used in combination with vincristine and prednisolone for advanced breast cancer**

V.L. Hall, R.B. Buchanan & C.J. Williams

*Combined Breast Clinic, Royal South Hants Hospital, Southampton SO9 4PE, UK.*

Mitozantrone (M) is less toxic than doxorubicin (D) in controlled single agent trials though there is little comparative data when they are used in combination. In this trial patients with advanced breast cancer were randomly assigned to vincristine (V) 1.4 mg m$^{-2}$ (max. 2.0) i.v. D1 and 8, D 40 mg m$^{-2}$ i.v. D1 and 8, prednisolone (P) 12 mg m$^{-2}$ PO D1–14 q28 days (VAP) or the same doses of V and P together with M 14 mg m$^{-2}$ i.v. D1 and 8 (VMP). Sixty patients were randomized (30 to each arm). They were well balanced for prognostic factors (age, menopausal status, disease free interval, sites of disease). Overall response rates were VMP 48% and VAP 44%. Median response duration was VMP 9.6 mo, VAP 5.9 mo (log rank $P=0.2$). Median survival was VMP 14.6 mo VAP 10.5 mo (log rank $P=0.5$). There were no significant differences (Mann–Whitney U test) for the following toxicities: nausea, stomatitis, performance status, neuromuscular. All patients were offered head cooling and most started this (VAP 25, VMP 24). Alopecia was significantly more severe for VAP (third cycle $P=0.0002$, sixth cycle $P=0.02$), though most VMP patients had WHO 2 toxicity by the sixth cycle. By the third cycle anaemia and neutropenia were significantly more severe in VMP ($P=0.01$). One VAP patient died of heart failure, in remission, 6 mo after chemotherapy. One other had WHO grade 2 toxicity. One VMP patient died of sepsis during chemotherapy. Quality of life assessment revealed no significant differences between VMP and VAP. Using this combination the only significant reduction in subjective toxicity for M was a marginal reduction in alopecia. This is at the expense of significantly greater neutropenia and anaemia. There was no cardiac toxicity in the VMP regime.

### Mitozantrone or doxorubicin with vincristine and cyclophosphamide (VNC *vs.* VAC) in the treatment of advanced breast cancer

A.J. Slater[1] & J.A. Green[2] for the Mersey Breast Group

[1]*Mersey Regional Centre for Radiotherapy and Oncology and CRC Department of Radiation Oncology, Clatterbridge Hospital, Merseyside L63 4JY, UK.*

This randomised study asks the question whether mitozantrone in combination can achieve equivalent disease control with reduced toxicity compared to doxorubicin. Seventy-one patients (13 pre-, 57 post-menopausal and 1 male) with a mean age 54.5 (range 28–77) have been randomised to receive either vincrinstine $1.4\,mg\,m^{-2}$ i.v., mitozantrone $10\,mg\,m^{-2}$ i.v., cyclophosphamide $600\,mg\,m^{-2}$ i.v. (VNC), or vincristine $1.4\,mg\,m^{-2}$ i.v., doxorubicin $50\,mg\,m^{-2}$ i.v., cyclophosphamide $600\,mg\,m^{-2}$ i.v. (VAC), both given with a cycle time of 21 days. The coded results are as follows: on arm A out of 36 patients there have been 10 CR, 12 PR, 5 SD, 1 PD and 8 NE with the median time to relapse in the responders of 33 weeks. On arm B out of 35 patients there have been 3 CR, 13 PR, 12 SD and 7 PD and the median duration of response was 24 weeks. The mean number of cycles given on arm A was 4.4 and arm B 4.1.

WHO grade 3/4 alopecia was observed in 23 patients on arm A and 10 patients on arm B. Nausea and vomiting grade 3/4 was seen in 13 patients on arm A and 11 patients on arm B. Myelosuppression was similar in both arms. No patient has discontinued chemotherapy on account of bone marrow toxicity, but one patient experienced profound hypocalcaemia in VNC and treatment was stopped. There have been no toxic deaths. The present study has shown important differences in response rate, duration of remission and toxicity between VAC and VNC in the preliminary analysis, and remains open to entry.

### Chemotherapy in poor risk germ cell tumours: An independent evaluation of POMB/ACE (*cis*-platinum, vincristine, methotrexate, bleomycin/actinomycin-D, cyclophosphamide, etoposide)

N.S. Stuart[1], M.H. Cullen[1], P. Harper[2], C.M. Woodroffe[1], P. Kirkbride[2] & J. Clarke[2]

[1]*Queen Elizabeth Hospital, Birmingham; and* [2]*Guy's Hospital, London, UK.*

The seven drug, alternating, high-dose *cis*-platin regime, POMB/ACE, has been reported by the Charing Cross Group (CCG) as producing excellent results in all patients with advanced germ cell tumours. We have independently evaluated this regime in 60 poor risk patients; 55 patients with advanced teratoma and 5 with bulky, relapsed seminoma. Five teratoma patients had relapsed following previous chemotherapy (CT) or radiotherapy (XRT) or both, 13 had extragonadal tumours, 6 had hepatic and 4 cerebral metastases. Twenty-eight patients had 'high markers' by MRC criteria and 22 'very high markers' by CCG criteria. The primary testicular patients comprised 16 with 'large volume' and 21 with 'very large volume' disease by MRC criteria. The CT regime used was that reported by Newlands *et al.* (1983, 1986) with the difference that CT was continued for only two courses (one full cycle) beyond marker remission with a minimum of 5 courses. Intrathecal methotrexate was given to only one of the patients with cerebral metastases (mets) and none received XRT. Tumour masses remaining after CT were resected if possible.

The median number of courses of CT received by patients completing treatment was 6 (range 5–13). Median follow-up is 32 months. Overall 2-year actuarial survival (AS) is 74% with all 14 deaths occurring within 16 months of start of treatment. For the 'very high marker' group, 2-year AS is 60%; for the low marker group, 84%. These figures are very similar to equivalent groups in the CCG series. Using MRC prognostic criteria patients with 'large volume' and 'very large volume' disease had similar survival while the 'high marker' group had a significantly worse 2-year AS than the 'low marker' group (58% *vs*, 91%, $P<0.01$). Four of the 6 patients with hepatic mets and 3 of the 4 with cerebral mets are alive in remission. Sixty patients were evaluable for toxicity. Septicaemia occurred in 5 patients, auditory loss in 10, severe neuropathy in 2, persistent CT induced vomiting requiring re-admission in 5 and impaired renal function in 8. There were three treatment related deaths.

We conclude that POMB/ACE is an effective regime in the treatment of poor risk germ cell tumours even when a shorter treatment duration that originally described is used. We confirm the prognostic significance of high tumour markers. We feel the complexity and toxicity of this regime makes it inappropriate for the treatment of good risk patients.

### Intensive platinum based combination chemotherapy (BOP/VIP) for germ cell tumours with the worst prognosis

M. Harding[1], C. Bradley[1], C. Lewis[1], L. Mill[1], J.J. Keizer[1], M. Soukop[1] & S. Kaye[1]

[1]*Department of Medical Oncology, University of Glasgow, UK;* [2]*University of Leiden, The Netherlands; and* [3]*Glasgow Royal Infirmary, UK.*

Glasgow data show that patients with very high volume metastases (MRC definition) from testicular germ cell tumours or extragonal primary sites, have 2 year survival rates of 58% and 25% respectively. This pilot study was designed to improve these results. To date 27 patients (median age 33 years) have completed therapy with nodal disease $>10\,cm$ ($n=7$), advanced pulmonary metastases (number $>20$ and/or $>3\,cm$: $n=4$), brain ($n=1$), bone ($n=1$) or liver ($n=4$) involvement or extragonadal primary sites ($n=10$: all with lesions $>7\,cm$). Twenty-three of 24 patients with teratoma were marker producing: median HCG level $93{,}750\,u\,l^{-1}$ ($561-1.6\times10^{6}$), median AFP 6, $170\,u\,ml^{-1}$ ($125-73$, 800).

Treatment comprised 3 cycles of CDDP $50\,mg\,m^{-2}$ days 1 and 2 with vincristine 2 mg and bleomycin 30 mg day 1 at 7–10 day intervals (BOP): then 3 courses of CDDP $20\,mg\,m^{-2}$, ifosfamide $1.2\,mg\,m^{-2}$ days 1–5 with VP16 75–100 $mg\,m^{-2}$ days 1, 3, 5 (VIP) q 3 weekly. Grade 4 myelotoxicity necessitated reduction in VP16 dosage in 7 of 10 patients receiving $100\,mg\,m^{-2}$: 2 septicaemias occurred. Toxicity requiring protocol modification was neuropathy ($n=2$) and renal failure ($n=1$); 3 patients developed significant neuropathy after completion of BOP/VIP.

Twenty-six patients are evaluable for response: 12 achieved surgically confirmed complete remission (CR) and immature teratoma was resected from a further 4. Three patients reached clinical CR and 6 a partial remission (PR) only; of these 2 continue on treatment but 4 have relapsed. A single patient had a marker PR but no radiological response. Eighteen patients are disease free with a median follow-up of 12 months (6–24). A further 16 patients are currently on treatment and accrual continues.

**Intensive weekly combination chemotherapy for initial treatment of advanced intermediate and high-grade non-Hodgkin's lymphoma (NHL)**

J.W. Sweetenham, J.M.A. Whitehouse & G.M. Mead

*CRC Wessex Medical Oncology Unit, Southampton General Hospital, Southampton, Hants. SO9 4XY, UK.*

High response and survival rates have been reported for the MACOP-B regimen in aggressive NHL (*Ann. Int. Med.*, **102**, 506, 1985). The major toxicity of this regimen was mucositis related to methotrexate (MTX). We have devised a similar regimen for a comparable group of patients with a reduced dose of MTX, but with the addition of etoposide.

From June 1986 to November 1987 30 patients (pts), median age 58 (33–69), with NHL (intermediate and high grade) entered a prospective trial of intensive weekly chemotherapy comprising: A – Adriamycin 35 mg m$^{-2}$ i.v. d1, cyclophosphamide 300 mg m$^{-2}$ i.v. d1, etoposide 150 mg m$^{-2}$ i.v. d1. B – MTX 100 mg m$^{-2}$ i.v. d8 (with LV rescue), bleomycin 10 mg m$^{-2}$ i.v. d8, vincristine 1.4 mg m$^{-2}$ i.v., d8.

A and B given in a 2-weekly cycle for 6 cycles of each. Prednisolone 50 mg daily given weeks 1–4 then on alternate days weeks 5–12. Prophylactic co-trimoxazole given throughout. Dose modifications exactly as for MACOP-B. Intrathecal MTX and cytosine arabinoside were given to marrow + pts.

*Patient characteristics:* Number currently evaluable – 20. Histology (WF): DLC (14), DM (4), Immunoblastic (2); Stage: II (>10 cm) (2), III (9), IV (9) (Marrow +5). B symptoms (8). Outcome: 12/20 CR (2) subsequent relapses (1 death). 3/20 PR but with resolving radiological abnormalities. 2/20 PR with subsequent progression (1 death).

One neutropenic death. One death from gastric perforation on treatment. 1 death from WI on treatment.

The dose limiting toxicity has been mucositis. Myelosuppression has not been severe. This intensive regimen has proved to be effective in tis preliminary study, with manageable toxicity. Updated results will be present.

**Spinal cord compression in small cell lung cancer**

R.L. Souhami[1], C.M. Ash[1] & J.M. Goldman[2]

[1]*Department of Oncology, University College London and Middlesex School of Medicine, London;* [2]*Brompton Hospital, London, UK.*

The records of 610 consecutive patients taking part in a randomised trial in small cell lung cancer were reviewed and 24 (4%) cases of spinal cord compression (CC) identified. Five hundred patients had isotope bone scans performed, and in 131 (26%) there was abnormal isotope uptake in the spinal column, only 7% of these patients developed CC. However, of the 25 patients who presented with back pain and had a positive bone scan affecting the spine, 36% developed CC. The incidence of cerebral metastases in the trial was 19.5% and in patients with CC 45%. The combination of cerebral metastases and a positive bone scan gave a 25% chance of developing CC. There were 2 distinct forms of clinical presentation. Six patients (Group A) presented with CC, all had back pain and positive bone scans, 5 out of 6 had sphincter disturbance. Median survival from CC was 30 weeks. Eighteen patients (Group B) developed CC whilst on treatment, 28% had positive bone scans, 44% back pain and 61% sphincter disturbance, median survival from CC was 4 weeks. Overall median survival was 30 weeks in Group A, 34 weeks in Group B and 37 weeks in the multicentre trial. Three patients received surgical treatment (survival 17, 55 and 57 weeks). Fourteen patients received radiotherapy, median survival 6 weeks, and 7 patients received symptomatic treatment, median survival 4 weeks. CC is an important cause of morbidity and mortality in small cell lung cancer. The study shows that it is possible to select patients at high risk (those with back pain and positive bone scans, and those with cerebral metastases and positive bone scans) who should receive radiotherapy as prophylaxis against this complication.

**Mathematical modelling in locally advanced breast cancer (LABC)**

M.A. Richards, W.M. Gregory & R.D. Rubens

*ICRF Clinical Oncology Unit, Guy's Hospital, London on behalf of the EORTC Breast Cancer Cooperative Group, UK.*

A mathematical model has been developed to study mechanisms of tumour response and applied to data from a study of LABC. Patients were randomly allocated to treatment with radiotherapy (RT), RT + chemotherapy (CT), RT + endocrine therapy (ET) or RT + CT + ET. The best fit curves derived by the model give the mean ±s.d. of the number of residual tumour cells and the mean and spread of tumour doubling time (DT).

The model fitted the actuarial curves for time to local progression well for RT and RT + CT. It was estimated that the addition of CT reduced the mean number of local tumour cells by an extra 2 logs. The DTs for RT and RT + CT were similar (means 21.4 and 23.5 days). The model gave only a poor fit for the actuarial curve for patients treated with RT + ET. Further application of the model to separate intervals on this curve suggests that ET resulted in a cell kill similar to that of CT and also caused growth arrest in a proportion of patients for a mean of 14 months. Growth thereafter was similar to that for patients treated with RT only. For time to distant metastases, neither CT nor ET reduced the micrometastatic tumour burden significantly, when compared with that estimated for patients receiving RT only. The model estimates for the doubling times of locally recurrent tumours and distant metastases following RT alone or RT + CT were similar.

The results suggest either a difference in chemosensitivity between primary and metastatic cells or that RT enhances response to CT. The model will be of value in the design of new clinical trials.

**Combination chemotherapy for adenocarcinoma of unknown origin**

T.S. Ganesan, P. Clark, R.J. Osborne, S. Malik, M.L. Slevin, P.F.M. Wrigley & R.I.D. Oliver

*ICRF Department of Medical Oncology, St Bartholomew's Hospital, Smithfield, London EC1A 7BE, UK.*

Poorly differentiated adenocarcinoma or undifferentiated carcinoma, is a common problem in clinical practice. Treatment of this clinical syndrome with teratoma-like chemotherapy may result in long-term remission for a proportion of younger patients (Greco *et al.*, *Ann. Int. Med.*, **104**, 547, 1986), though careful selection of patients with particular disease patterns may be required. From 1983 to 1987, 15 patients (10 females and 5 males), with metastatic adenocarcinoma of unknown primary origin was treated with BEP, bleomycin 30 mg day$^{-1}$ i.v. infusion days 1–3, *cis*-platinum 40 mg m$^{-2}$ i.v. days 1–3, etoposide 120 mg m$^{-2}$ i.v. days 1–3, repeated every 21 days, at least 3–4 courses were given to each patient.

The median age of the patients was 38 years with a range between 22–44 years. At presentation the sites of metastasis in patients were lung 12, liver 6, bones 7, brain 2, lymph-nodes 5, skin 1 and fundus 2. Two of 15 patients had disease confined to the lung, one confined to the bone, and one to the pelvis. Alpha-foetoprotein and HCG levels were normal in all patients. Fourteen of 15 patients had poorly differentiated or undifferentiated adenocarcinoma and one patient had a well differentiated tumour. Five of 15 patients (2 CR, 3 PR) responded to therapy, 6 of 15 patients were unresponsive, and there were 4 of 15 early deaths (after one cycle of treatment). One patient with CR relapsed 2 months after completion of therapy, and the other patient having had previous radiation to the chest died in CR due to bleomycin toxicity. Two of 15 patients are alive (1 PR, 1 fail) at the time of the report. Lung toxicity was observed in 6 of 15 patients (assessed by PFT and chest X-ray) and in 1 patient it was fatal. Renal toxicity was observed in 4 of 15 patients and in 2 of 15 it was fatal. Post mortem examination was performed in 6 of 15 patients: 3 patients had a primary tumour in the lung, 1 patient died of severe bleomycin toxicity (having had previous chest irradiation) without any residual tumour detected, and in 2 patients a primary site could not be located. This regime was considerably more toxic in this group of patients than in the younger patients with teratoma receiving the same regimen, and there were no long-term survivors.

### A randomised double-blind cross-over trial of dexamethasone and lorazepam with or without high dose metoclopramide infusion in the treatment of chemotherapy induced emesis

M.E.R. O'Brien[1], M.H. Cullen[1], C. Woodroffe[1], K. Kelly[1], C. Burman[1], K. Palmer[2], G.R.P. Blackledge[2] & J. Sharpe[2]

[1]*Cancer Research Campaign, Clinical Trials Unit, Queen Elizabeth Hospital and* [2]*St Chad's Hospital, Birmingham, UK.*

High dose metoclopramide (MET) is an effective anti-emetic for use with *cis*-platin containing chemotherapy regimens but can cause extrapyramidal reactions (EPR). Lorazepam (L) and dexamethasone (D) are being used increasingly to alleviate chemotherapy induced emesis. This trial addresses the question 'does high dose MET contribute to anti-emetic control when given with dexamethasone and lorazepam? Eighty-one patients have entered a randomised double blind cross over trial comparing lorazepam and dexamethasone with or without high dose MET. Lorazepam 2 mg m$^{-2}$ was given with dexamethasone 8 mg i.v. and either MET 1 mg kg$^{-1}$ or a placebo of N Saline 60 min before chemotherapy. This was followed by a 24 h infusion of MET 9 mg kg$^{-1}$ or placebo with 4 mg boluses of dexamethsone 4 hourly i.v. If there is no vomiting patients then receive oral dexamethasone 4 mg qds plus either MET 20 mg qds or placebo tablets for 3 days. Patients were crossed over for the second course of treatment and then chose their preferred regimen for subsequent treatments. Randomisation was stratified according to whether or not the patient will be receiving *cis*-platin. Fifty-four patients are assessable having completed two courses and stated a preference, 29 received LD Met first and 25 LD Plac first. The chemotherapy regimens were given for a range of gynaecological and other malignancies, 48 contained *cis*-platin and 6 non-*cis*-platin regimens. The mean number of episodes of vomiting during the first 24 h for first and second courses (see Table) was significantly fewer when receiving LD Met than with LD Plac ($P<0.0001$).

| *Course* | *LD Met* | *LD Plac* |
|---|---|---|
| 1st | 2 | 7 |
| 2nd | 5 | 6 |

The difference in nausea control also significantly favoured the 3 drug regimen ($P<0.0004$). However, there was no difference in the control of nausea and vomiting during the following 3 weeks. Nausea was significantly more prevalent during the period after the 2nd course of chemotherapy compared with the 1st, irrespective of anti-emetic regimen ($P<0.007$). Vomiting was completely abolished in 45% of patients, and 69% had major control ($\leq 2$ episodes) when they received LD Met on first exposure to chemotherapy. More patients stated a preference for the 3 drug regimen although this is not statistically significant. EPR were recorded in 14% of patients receiving MET. In conclusion the combination of dexamethasone and lorazepam can give major control of emesis in 25% of patients but with the addition of metoclopramide the control of both nausea and vomiting are significantly improved.

### BRL 43694, a selective 5-$HT_3$ receptor antagonist: Dose ranging study for control of *cis*-platin induced emesis

J. Carmichael[1], B. Cantwell[1], G. Rapeport[1], P. Coates[1], S. Thompson[1] & A.L. Harris[1]

[1]*University Department of Clinical Oncology, Newcastle upon Tyne; and* [2]*Beecham Pharmaceuticals Research Division, Harlow, Essex, UK.*

Thirty-eight patients receiving *cis*-platin (CP), either alone or in combination, were treated with BRL 43694 (BRL), a new selective 5-$HT_3$ receptor antagonist. BRL was administered as a 30 min infusion immediately following a 30 min CP infusion. The starting BRL dose was 10 $\mu$g kg$^{-1}$, with 8 patients receiving up to 30 $\mu$g kg$^{-1}$ with clinical benefit observed above 20 $\mu$g kg$^{-1}$. Of 14 pts treated at 40 $\mu$g kg$^{-1}$ BRL, complete control of CP induced nausea and vomiting was achieved in 8 (57%), over 24 h, with the onset and severity of these symptoms significantly modified in the remaining 6 patients. Symptoms of nausea were assessed using linear visual analogue scores (VAS) and global rating scores. No unexpected toxicities were observed, as determined by a systematic enquiry covering 35 general questions, and in particular, symptoms of anxiety or sedation were not problematical. 24 h Holter monitoring revealed no significant arrhythmias. Detailed pharmacokinetic studies were performed at BRL doses of 40 $\mu$g kg$^{-1}$ and these showed wide inter-individual variation: Mean values (range) for $C_{max}$ 33 ng ml$^{-1}$ (11–81); Cl 0.235 (0.035–0.602) l h$^{-1}$ kg$^{-1}$; VD 2.29 l kg$^{-1}$ (1.06–4.2); AUC 353 ng k ml$^{-1}$ (66–1127) and $t_{z1/2}$ 10.4 h (2.9–21).

One patient with SVC obstruction developed transient renal impairment following CP, and in this patient BRL levels remained elevated over 24 h, suggesting that the drug is predominantly excreted by the kidney. BRL is an effective, safe and well tolerated anti-emetic for the control of CP induced nausea and vomiting. Further studies have been performed at BRL doses of 80 $\mu$g kg$^{-1}$ (3 pts) and 120 $\mu$g kg$^{-1}$. Sixteen patients have received the latter dose either as a single 30 min infusion or by a split dose schedule. Both of these schedules have been effective with no acute side effects.

### Balancing the possible benefits against the risk of cytotoxic chemotherapy – Patients' and doctors' decisions

M.L. Slevin, H. Plant, L. Stubbs, P. Wilson & K. O'Sullivan

*ICRF Department of Medical Oncology, St Bartholomew's and Homerton Hospitals, London, UK.*

Estimates of patients' willingness to tolerate adverse side effects of cytotoxic chemotherapy are largely a matter of clinical intuition. A study has been conducted to investigate how patients balance the benefit and cost of chemotherapy and to compare this to the response of cancer specialists to the same questionnaire. One hundred and six patients and 165 cancer doctors (60 medical oncologists, 87 radiotherapists, 18 others), completed questionnaires which invited responses to two fictional cytotoxic treatments (intensive treatment and mild treatment). All subjects were asked to assess their willingness to undergo these two treatments in three hypothetical situations: (1) chance of cure; (2) prolongation of life; (3) relief of symptoms. There were significant differences between patients and doctors responses in each of the three parameters in both treatment situations. Offered intensive treatment 53% of patients were willing to accept 1% chance of cure whereas 11% of doctors would do so ($P=0.001$). In the prolongation of life question 42.1% of patients would accept treatment which might extend their lives by 3 months; 3.7% of doctors would do the same while 75% of doctors would require an extension of at least a year. Offered treatments with expected mild toxicity, both patients and doctors were prepared to accept a lower chance of success. Sixty-seven percent of patients and 39% of doctors would accept a 1% chance of cure. Prolongation of life for the minimum 3 months was acceptable to 53.8% of patients and 27% of doctors ($P<0.0001$). These data suggest that most patients are prepared to undergo intensive therapy for very little chance of benefit and may be unable to make rational decisions about treatment on their own.

### Posters

### A randomised double blind placebo controlled trial of medroxyprogesterone acetate (MPA) in cancer cachexia

D.C. Talbot, M.L. Slevin, S.P. Joel, L. Stubbs, H. Plant, A. Allbright, D. Lynch & B. Hemmings

*ICRF Department of Medical Oncology, St Bartholomew's and Homerton Hospitals, London, UK.*

Patients with breast cancer treated with MPA often report an increased appetite. MPA has a potential advantage over corticosteroids in that it does not exert a catabolic effect. MPA (100 mg tds orally) has therefore been compared with placebo in 60 patients with advanced malignant diseae (33 Ca lung (26 oat-cell, 7 non-oat-cell), 5 mesotheliomas, 3 Ca colon, 3 NHL, 3 Ca ovary, 2 Ca breast, 2 myelomas, 9 other). Twenty-one patients in the MPA group and 20 in the placebo group were receiving chemotherapy. Patients were treated for 6 weeks and were assessed at weeks 0, 3 and 6 for appetite, mood and energy using linear analogue scales and serum proteins as indicators of nutritional status. There was a significant improvement in appetite in the MPA group between assessment 1 and 2 ($P=0.0002$) and 1 and 3 ($P=0.015$). There was significant improvement in appetite in the placebo group. This improvement in appetite in the MPA occurred in both patients with oat-cell lung cancer and those with other malignancies. Supporting this finding was the significant increase in serum thyroid binding pre-albumin and retinol binding protein in the MPA group between assessment 1 and 3 ($P=0.023$ and $P=0.039$ respectively). These two parameters showed no significant change in the placebo group. There was no change in performance status, energy, mood or pain in either group. This data indicates that there is an apparent increase in appetite in anorexic patients with advanced cancer treated with MPA which is reflected in increases in protein indicators of nutritional status. However, this apparent increase in appetite did not result in improved weight, performance status or mood.

### Immunohistochemical analysis of C-erbb$_2$/her-2/neu in breast tumours

P. Johnston[1], P. Dervan[1], A. McCann[1], L. Doyle[1], B. Guillick[2] & D.N. Carney[1]

[1]*Mater Misericordiae Hospital, Dublin, Ireland; and* [2]*Imperial Institute of Cancer Research, London, UK.*

Using an immunohistochemical technique we have evaluated and compared the expression of C-erbb$_2$ oncogene and ER status in 95 primary breast tumours. Results of C-erbb$_2$ expression were also correlated to lymph node status and tumour grade. In 29 of 95 (30%) tumours intensive membrane staining indicative of C-erbb$_2$ was noted using the monoclonal antibody to the 21 N peptide (C terminus residues 1243–1255). In positive specimens heterogeneity of expression was noted throughout. Of the 29 positive tumours axillary lymph node metastases were present in 25 of the cases (86%). Among these 29 cases the average age was 50 years (range 36–79). No correlation was noted between C-erbb$_2$ expression, ER status and tumour grade (Table). Among the 66/95 (70%) of C-erbb$_2$ negative tumours lymph node metastases were detected in only 27% and the average age was 56 years (range 32–85).

| *C-erbb$_2$ expression* | *Number* | *Mean age* | *% ER+* | *% Grade III* | *%LN+* |
|---|---|---|---|---|---|
| Positive | 29 | 50 | 50 | 65 | 86[a] |
| Negative | 66 | 56 | 65 | 46 | 27 |

[a]$P<0.001$.

These data suggest a strong correlation between C-erbb$_2$ amplification in breast cancer and axillary lymph node metastases. Thus the presence or absence of C-erbb$_2$ gene amplification may be a significant predictor of disease progress and long term survival in patients with breast cancer.

### Mitozantrone and ifosfamide in metastatic breast cancer

D. Parker. M.K. Gough, A.K. Hancock, M.B. Masood, B. Gadsby-Peet & J.J. Price

*Oncology Unit, Bradford Royal Infirmary, Bradford BD9 6RY, UK.*

The combination of an anthracycline and alkylating agent has been shown to be synergistic in both animal models and clinical breast cancer. We have used the combination of mitozantrone (10 mg m$^{-2}$ i.v.) with ifosfamide and MESNA 1 gm$^{-2}$ i.v. days 1–3 as an infusion. The treatment was repeated every 3 weeks. The dose of both drugs was designed to increase the therapeutic index of the combination and in particular to reduce both nausea and alopecia.

Twenty patients have been treated with this combination, four of whom were pre-menopausal. Thirteen patients had local disease, 9 had pleural or pulmonary disease and 9 had bone metastases. Seven (35%) patients showed a partial response to the combination and 2 patients had static disease. Toxic effects included moderate neutropenia without clinical infection and frequent nausea/vomiting. Noticeable alopecia affected the majority of patients.

This combination shows moderate activity in metastatic breast cancer, but the regimen has not substantially reduced the nausea and alopecia associated with alkylator/anthracycline combinations.

### A phase II study of mitozantrone in advanced breast cancer

P.I. Thompson & V.J. Harvey

*Department of Clinical Oncology, Auckland Hospital, Auckland, New Zealand*

Mitozantrone is a synthetic anthracenedione which has been reported to produce a similar response rate to doxorubicin in advanced breast cancer with a relative absence of acute side effects. Fifty-six patients with advanced breast cancer have been treated. Mean age was 53 years (range 35–75 years) and average Karnofsky score at commencement of therapy 72%. Initial dosage was $12\,mg\,m^{-2}$ with provision for escalation. Fifty-three patients are evaluable for response, all having completed therapy. Eleven patients (21%) achieved a partial remission with remission durations of 4–16+ months. Ten patients (19%) had static disease and maintained this for 3–19+ months Thirty-one patients (58%) had progressive disease. The response rate in previously treated patients was 13% (5/38) but increased to 40% in the 15 previously untreated patients. Improvement in quality of life as measured by a symptom score, linear analogue scale assessment (LASA) score, category score and Karnofsky performance status correlated with objective response. Therapy was well tolerated by patients. Haematological toxicity was significant with dose modification frequent, resulting in a mean dose achieved per 3 week cycle of $9.1\,mg\,m^{-2}$ both overall and in responding patients. Gastrointestinal toxicity was mild. Seventeen patients (30%) experienced some hair loss but in all this was only mild. There was no clinical or ECG evidence of cardiotoxicity. Mitozantrone offers a useful alternative to combination chemotherapy as first line treatment in less fit patients but its role in previously treated patients is less well defined.

### Aminoglutethimide and estramustine: Combined endocrine therapy in advanced breast cancer

D. Parker, M.K. Gough, A.K. Hancock, M.B. Masood, B. Gadsby-Peet & J.J. Price

*Oncology Unit, Bradford Royal Infirmary, Bradford BD9 6RJ, UK.*

Twenty-six post-menopausal patients with metastatic breast cancer were treated with escalating doses up to a maximum of 1 G aminoglutethimide and 520 mg estramustine daily. Aminoglutethimide was given with estramustine as it was thought that estramustine might occupy oestrogen receptor sites vacated by the action of aminoglutethimide in reducing free oestrogen. The mean age of the patients was 55 years, mean 11 years post-menopausal. Nineteen patients had bone metastases demonstrated by scintiscan and 8 patients had liver metastases shown by computerised tomographic scan. Sixteen patients had local disease. Twenty had received adjuvant cytotoxic therapy and 23 had received previous endocrine therapy. There were two objective responses, one complete clinical response of local disease and one partial response in lung, bone and liver metastases. Eleven patients had nausea and vomiting which led to 3 patients stopping treatment.

This low response rate suggests that aminoglutethimide and estramustine are antagonistic. This may be due to induction of liver enzymes by the aminoglutethimide leading to enhanced hydrolysis of estramustine with release of free estradiol or estrone. The combination of aminoglutethimide and estramustine cannot be recommended for the treatment of advanced breast cancer.

### Weekly epirubicin for liver metastases in breast cancer

C.J. Twelves, R.E. Coleman, S. O'Reilly, M.A. Richards & R.D. Rubens

*ICRF Clinical Oncology Unit, Guy's Hospital, London, UK.*

Patients with breast cancer who develop liver metastases have a poor prognosis. Administration of chemotherapy is difficult; in particular, anthracyclines may cause severe toxicity because of reduced hepatic drug clearance. We have studied the efficacy and toxicity of epirubicin using a weekly schedule, adjusting dose intensity according to myelosuppression.

Between November 1986 and February 1988 we treated 28 consecutive patients with liver metastases demonstrated by isotope or ultrasound scan, who had an AST $>2\times$ normal or bilirubin $>20\,\mu mol\,l^{-1}$. Median age was 56 years (range 27–78) and median UICC performance status was 2 (range 1–3). None had received prior anthracyclines, but 6 had received other chemotherapy (2 as adjuvant treatment). Epirubicin $25\,mg\,m^{-2}$ i.v. was given weekly. Dosage adjustments were not made for abnormal liver biochemistry, but if the WBC $<2.0\times10^9\,l^{-1}$ or platelets $<70\times10^9\,l^{-1}$, treatment was delayed until these levels were reached.

It is too early to assess response in 2 patients. Of the 26 patients assessable to date, 1 had a complete remission and 8 a partial remission (UICC); 3 others had a >50% improvement in liver biochemistry. All responders improved symptomatically. During the first month of treatment, 6 of the 12 responding patients had a >25% deterioration in AST or bilirubin, but liver biochemistry subsequently improved as chemotherapy continued. All patients are evaluable for toxicity; 4 had WHO grade III or IV vomiting, 1 grade III stomatitis and 6 grade III myelosuppression.

In patients with advanced liver metastases from breast cancer, weekly epirubicin is well tolerated and has a >30% response rate. An initial deterioration in liver biochemistry may occur before there is a response.

### APD treatment of bone metastases from breast cancer

R.E. Coleman, P. Woll & R.D. Rubens

*ICRF Clinical Oncology Unit, Guy's Hospital, London SE1 9RT, UK.*

Osteoclastic bone destruction is inhibited by the biphosphonates and repeated administration may achieve long-term control of oesteolysis. In this study we have investigated the effect of APD (3 amino-1, 1-hydroxypropylidene bisphosphonate) on bone metastases from breast cancer.

Twenty-eight patients with progressive symptomatic bone metastases received APD 30 mg as a 2 h intravenous infusion every 14 days. No other systemic therapy for breast cancer was prescribed and APD continued until the development of progressive disease. Patients were assessed for objective response by the UICC criteria. In addition, subjective response was determined by a pain questionnaire and bone metabolism monitored by serial biochemical measurements.

Response in bone assessable in 24 patients. Evidence of bone healing with sclerosis of lytic metastases (PR) occurred in 4 patients. The median duration of response was 10+ months. Ten patients had stable disease for at least 3 months

(median 5 months), and 10 progressed. Symptomatic response occurred in 10 patients. Biochemical monitoring indicated selective inhibition of osteolysis with no effect on oestoblast function. Treatment was tolerated well without toxicity.

APD is a potent inhibitor of osteolysis and a valuable new treatment for the palliation of bone metastases. The role of APD in conjunction with established systemic therapy is to be studied in a randomised phase III study.

### Single high dose (45 mg) infusions of aminohydroxypropylidene diphosphonte (APD) for severe malignant hypercalcaemia

B.M.J. Cantwell, J. Carmichael, K.A. Mannix & A.L. Harris

*University Department of Clinical Oncology, Newcastle General Hospital, Newcastle upon Tyne NE4 6BE, UK.*

We previously found that single i.v. infusions of 30 mg APD were effective in lowering serum calcium and induced normocalcaemia in 10 (63%) of 16 patients with malignant hypercalcaemia. Those that did not achieve normocalcaemia tended to have the highest pretreatment serum calcium levels (Cantwell & Harris, *BMJ*, **294**, 467, 1987). To induce normocalcaemia in a greater proportion and to maintain the advantages of single infusion therapy we gave single infusions of 45 mg APD in 750 ml normal saline, infused i.v. over 3 h to 27 patients with malignant hypercalcaemia. Intravenous hydration with 3 l of normal saline daily was given with APD infusions. Serum calcium levels were adjusted for albumin. The majority had severe hypercalcaemia, MEAN serum calcium was 3.57 mmol l$^{-1}$ (normal range 2.25–2.75 mmol l$^{-1}$) and 15 patients had levels >3.5 mmol l$^{-1}$ with 2 having levels >4 mmol l$^{-1}$. There were 16 males and 11 females, 8 had non-small cell lung cancer, 7 had breast cancer and the remainder a variety of other cancers. Sixteen were resistant to prior calcium lowering therapies. After a single 45 mg infusion of APD 22 (81%) patients achieved normocalcaemia, including 2 patients with pre APD serum calcium levels >4 mmol l$^{-1}$. One patient only showed no response and the mean NADIR level after APD in 26 patients who responded was 2.57 mmol l$^{-1}$. Three patients had mild temporary hypocalcaemia and 1 patient whose pre-APD serum calcium was 3.20 mmol l$^{-1}$ had prolonged hypocalcaemia after APD (serum calcium = 1.99 mmol l$^{-1}$ day 26). Single infusions of APD 45 mg are well tolerated and effective for the majority of patients with severe malignant hypercalcaemia.

### High dose etoposide at varying schedules for metastatic bronchogenic carcinoma

W.P. Steward[1], P.M. Wilkinson[2], J.M. Edmundson[2] & N. Thatcher[1]

[1]*CRC Department of Medical Oncology and* [2]*Department of Clinical Pharmacology, Christie Hospital and Holt Radium Institute, Manchester M20 9BX, UK.*

Sixty previously treated patients (pts) with metastatic small cell and non-small cell lung cancer were treated with up to 6, 3-weekly intravenous infusions (inf) of etoposide given either over 45 min or 24 h. Dosages used were either 600 mg m$^{-2}$ or 900 mg m$^{-2}$.

Median follow-up was 7.5 months (range 1–32 months). Partial responses were seen in 2 pts and disease was static during treatment in 11 pts. Duration of response was short (6 and 9 weeks). Median survival from starting treatment was 3 months for all patients – no significant difference between treatment schedules or according to histology. There were two treatment related deaths.

Pharmacokinetic data from 26 pts (using HPLC analysis of serial serum specimens over 48 h following drug administration) showed peak drug concentrations were 10-fold higher with 45 min infusions compared with 24 h inf (mean [$\mu$m] – 390 *vs.* 28.9 at 600 mg m$^{-2}$ and 394 *vs.* 36.9 at 900 mg m$^{-2}$). The AUC increased proportionately and significantly with the higher dose by either short or 24 h inf (mean + s.d. [$\mu$mol . l$^{-1}$]; 1,006 ($\pm$160) for 600 mg m$^{-2}$ inf; 842 ($\pm$218) for 600 mg m$^{-2}$ 24 h inf; 1,419 ($\pm$262) for 900 mg m$^{-2}$ short inf; 1,038 ($\pm$424) for 900 mg m$^{-2}$ 24 h inf).

The AUC was greater for the 45 min compared to the 24 h inf, for each dose but this was not significant. The higher weak concentration and greater AUC produced by bolus administration requires further investigation in more sensitive tumours.

### Disease relapse in patients with stage I non-seminomatous germ cell tumour of the testis on active surveillance

P.I. Thompson & V.J. Harvey

*Department of Clinical Oncology, Auckland Hospital, Auckland, New Zealand.*

Thirty-six patients with apparent stage I non-seminomatous germ cell tumour (NSGCT) of the testis were treated by inguinal orchidectomy and intensive follow-up only. Assessment included measurement of serum alphafetoprotein and beta HCG (tumour markers) and chest X-ray monthly for 1 year, then 2 monthly for 1 year, with CT scans of abdomen and chest repeated 3 monthly for the first year and 6 monthly for the second year. Median follow-up was 33.5 months (range 5–87 months). Relapse occurred in 12 patients (33.3%) at a median of 7 months (range 2–28 months). A further patient developed a pure seminoma in the other testis at 15 months and another had a para-aortic mass detected on CT scan at 15 months. Laparotomy was performed and histology revealed a differentiated teratoma. Elevated markers were of limited importance in relapse detection confirming the need for close clinical and radiological follow-up. Of 9 histological factors examined in the primary tumour only the presence of lymphatic invasion was associated with a significantly higher relapse rate. All patients were treated at relapse with *cis*-platinum based chemotherapy. Four underwent surgery in addition, 2 before and 2 after chemotherapy. Eleven were rendered disease free but 4 had a second relapse. One patient has died, one is alive with disease in fourth relapse, and 10 are disease free. Chemotherapy failed to cure 6 patients who had relapsed, but bulk of disease was not a factor. Despite the good overall result reported here, optimal post-orchidectomy management of stage I disease remains to be defined.

### Expression of $HMFG_2$ antigen in small cell carcinoma of the lung: Relationship to prognosis

F.G. Hay, R. Milroy[2], L.W. Adams[1], S.G. Allan[1] & R.C.F. Leonard[1]

[1]*Imperial Cancer Research Fund Medical Oncology Unit, Department of Clinical Oncology, Western General Hospital, Edinburgh; and* [2]*Cancer Research Campaign Department of Medical Oncology, Glasgow, UK.*

Despite the use of modern chemotherapy, long-term disease-free survival can only be expected in approximately 10% of patients with small cell lung carcinoma (SCLC), even including those with limited disease. It is therefore important to identify the group of patients with better prognosis so that appropriate therapy can be chosen at the outset.

Recently we reported an inverse association between $HMFG_2$ antigen expression and survival in SCLC patients (Allan *et al*., *Br. J. Cancer*, **56**, 485, 1987). We have now extended the preliminary study of $HMFG_2$ expression in pre-treatment bronchial biopsies to include a further 21 patients, making a total of 52 tumour specimens. As before, formalin fixed, paraffin embedded material was studied using a standard immunoperoxidase (PAP) staining procedure following enzyme digestion of the tissue sections.

Overall, median survival was 8 months for those expressing $HMFG_2$ antigen compared with 11 months for those who were negative ($P<0.01$ Mann–Whitney U). In the group of patients with limited disease ($n=40$), the same statistical difference was apparent with median survival of 8 months in 22 patients who were positive for $HMFG_2$ and 12 months for the 18 patients who were negative ($P<0.01$). The value of $HMFG_2$ as a prognostic marker merits study in a wider assessment of pre-treatment prognostic variables.

### Survival of patients with poor prognosis myeloma

D. Dodwell & I. McGill

*Department of Medical Oncology, Torbay Hospital, Torquay, S. Devon, UK.*

Retrospective analysis of 133 patients with multiple myeloma treated at our district general hospital over the last 14 years was undertaken.

All patients presenting with stage 3A (13 patients) or stage 3B (24 patients (with renal impairment)) disease, according to the staging system of Durie & Salmon (*Cancer*, **36**, 842, 1975) were treated with a four drug chemotherapy regimen, given monthly and using: vincristine, 1.4 mg m$^{-2}$ (max. 2 mg), cytosine arabinoside, 150 mg m$^{-2}$, cyclophosphamide 600 mg m$^{-2}$, all given on days 1 and 8 by i.v. bolus injection and prednisolone 30 mg m$^{-2}$ orally on days 1 through 8, along with standard supportive measures.

Of these 37 patients 14 died early in treatment prior to receiving two courses of chemotherapy (in the first 2 months after diagnosis).

Comparison of patient groups suggests that if patients with stage 3A or 3B myeloma survive this 2 month period then their survival thereafter is the same as the prognostically more favourable groups.

This we feel favours the use of intensive supportive therapy in the management of patients who present with poor prognosis myeloma, and we would advocate the use of combination regimens which can be safely used in the context of renal impairment, and which achieve rapid tumour kill.

### CMPP chemotherapy for intermediate and high grade non-Hodgkin's lymphoma in the elderly

S.W. Watkin & J.A. Green

*University of Liverpool Department of Radiation Oncology, Clatterbridge Hospital, Bebington, Wirral, Merseyside L63 4JY, UK.*

The CMPP regime was developed for the treatment of intermediate and high grade non-Hodgkin's lymphoma stage II–IV in elderly and poor performance status patients in whom doxorubicin containing regimens would be too toxic. Twenty-one patients (9M:12F) aged 52–79 mean 70 years, have been entered onto the study. There were 12 high and 7 intermediate grade (NCI Working Formulation) and 2 mycosis fungoides. Eighteen of 21 were stage IV and 6/16 had marrow involvement but no patient was leukaemic. Twelve (51%) had received prior treatment at a mean of 3.3 years before CMPP: 5 radiotherapy, 2 chemotherapy and 5 with both. Patients received chlorambucil 10 mg d$^{-1}$ d1-10, mitozantrone 10 mg m$^{-2}$ i.v. d1, procarbazine 150 mg d$^{-1}$ d1-10 and prednisolone 40 mg d$^{-1}$ d1-10, q21–28 d. The mean number of courses given was 3.7 and the mean cycle time was 30 d. Of 16 evaluable patients there were 6CR, 6PR, 4SD (overall RR 75%). Responders received a mean of 4.7 cycles against 2.4 in non-responders but there was no significant difference in cycle time (31 *vs*. 21 d). Median follow-up is 4 m and there have been 7 deaths at 0–11 m (2 early deaths in whom toxicity may have been contributory). Median survival is 11 m. Three patients developed neutropenic fever and 2 required transfusion but grade III/IV alopecia and vomiting were not seen. Sixteen of 78 courses were deferred in 9 patients and procarbazine was omitted after the first or subsequent cycles in 6 patients due to rash in 4 and myelosuppression in 2. The CMPP regime produces an acceptable response rate in this poor prognosis group with a median survival of 11 m. The dose limiting toxicity is haematological.

### CAPE/PALE salvage chemotherapy in relapsed Hodgkin's disease

D.H. Jones, J.M.A. Whitehouse & G.M. Mead

*CRC Medical Oncology Unit, Southampton General Hospital, Southampton SO9 4XY, UK.*

Since 1/84 16 patients (11 male, 5 female, age range 18–73 years, median 28 years) with poor prognosis relapsed Hodgkin's disease (HD) (<1 year post chemotherapy) have been treated with CAPE (cyclophosphamide 600 mg m$^{-2}$ i.v. d1; adriamycin 50 mg cm$^{-2}$ i.v. d1; adriamycin 50 mg cm$^{-2}$ i.v. d1; etoposide, either 500 mg m$^{-2}$ p.o. given over d2–5 or 250 mg m$^{-2}$ i.v. d1) and PALE (CCNU 80 mg m$^{-2}$ p.o. d1, with adriamycin, etoposide and prednisolone as above) given as alternating cycles at 21 day intervals for 6 cycles. At initial diagnosis 13 patients had NSHD, 3 MCHD; 5 patients were stage II, 11 stage III and 1 stage IV; 10 patients had B symptoms. Twelve patients had received prior chemotherapy only, 4 radiotherapy and chemotherapy, 6 having achieved a CR lasting 2–12 mo, median 4/12, 7 a PR and 3 progressive disease on therapy immediately prior to CAPE/PALE. All patients had relapsed at previously involved sites and at new sites in 3. The mean number of cycles of CAPE/PALE administered was 5.5 (range 1 to 7). Eight patients achieved a CR of whom 4 remain free of disease at 16, 31, 33 and 42 mo, and 3 a PR an overall response rate of 69%. Toxicity has been moderate. There were 8 episodes of neutropaenic fever, two of dermatomal HZV and one each of axillary cellulitis, infected central venous catheter, acute myocardial infarction (AMI) and cryptosporidial infection. There were no treatment related deaths. Treatment was delayed in 9 patients on 16 occasions and dose reductions made in 10 patients in 31 out of 86 cycles given, in 1 patient initially due to thrombocytopaenia and later AMI and in 9 due to neutropaenia.

CAPE/PALE is effective as salvage chemotherapy in relapsed Hodgkin's disease. Results are at least as good as alternative regimens with acceptable toxicity and fewer injections.

### CHLVPP chemotherapy in advanced Hodgkin's disease

J.M.A. Whitehouse, C.J. Williams, J. Sweetenham, D.H. Jones & G.M. Mead

*CRC Medical Oncology Unit, Southampton General Hospital, Southampton SO9 4XY, UK.*

From 3/78 until 1/87, 54 patients with Hodgkin's disease

(HD) were treated with chlorambucil 6 mg m$^{-2}$ (max 10 mg) p.o. d1–14; vinblastine 6 mg m$^{-2}$ (max 10 mg) i.v. d1 and d8. Procarbazine 100 mg m$^{-2}$ (max 200 mg) p.o. d1–14; and prednisolone 40 mg p.o. d1–14, at monthly intervals and a mean of 5.7 courses were administered. No patient received chemotherapy (CT), 12 had relapsed after radiotherapy (RT). Five patients received combined CT and RT for bulky mediastinal disease. Forty patients were male and 14 females (age range 15–73 years, mean 29). Sixteen patients were stage II, 27 stage III and 11 stage IV. Twenty-nine patients had B symptoms. Forty-two patients had NSHD, 6 MCHD and 6 LPHD. Mean follow-up time from completion of CT is 42 months (range 10–90). Forty-two (77.8%) patients achieved CR with 33 (61.1%) in continuous CR at a mean follow-up of 44 months (range 10–90). Ten patients (18.5%) died during follow-up, 1 with no clinical evidence of HD 59 months from CT, 1 during CT and 8 from HD. The regime was generally easy to administer and well tolerated. A mean of >90% of the planned dose of each drug was administered. Five patients required cessation of one of the regime's drugs and substitution with another agent because of side effects. Three patients had mild neuropathy, 2 moderate nausea, and no patient had alopecia. There were 3 episodes of neutropaenic fever, one of pneumococcal pneumonia and one of disseminated HZV. One patient died during CT from legionella pneumonia. Treatment was delayed in 8 patients on 10 occasions, 1 due to poor compliance and in 9 due to neutropaenia.

### 'PACET' intensive chemotherapy (CT) for relapsed lymphoma

J.J. McKendrick, J.M.A. Whitehouse & G.M. Mead

*CRC Medical Oncology Unit, Southampton General Hospital, Southampton SO9 4XY, UK.*

The outlook for patients with relapsed high and intermediate grade NHL (WF) and multiply relapsed HD is dismal. Evidence suggests marrow ablative CT and marrow rescue may improve survival. 'PACET' is highly marrow suppressive CT which can be given as repeated courses with acceptable and manageable toxicity without marrow rescue. Early results suggest activity in these patients. Sixteen heavily pretreated patients with relapsed high or intermediate grade NHL or HD were treated with CCNU 60 mg m$^{-2}$ p.o. d1–2; cytosar 100 mg m$^{-2}$ i.v. BD, VP16–213 100 mg m$^{-2}$ i.v., 6-TG 80 mg m$^{-2}$ p.o. and prednisolone 40 mg m$^{-2}$ p.o. all d1–5 repeated at d28. A mean of 1.9 courses (range 1–3) were given to 14 evaluable patients with median age 46 years (range 28–65). Ten had DLC, 1 immunoblastic and 1 small non-cleaved lymphoma, 1 HD and 1 malignant histiocytosis. All patients had shown at least PR to prior CT. Two patients (14%) achieved CR and are disease free at 2 and 14 months. One patient had a PR lasting 6 months and is alive at 24 months. Overall response is 21%. Two further patients had stabilization of disease (s.d.) for 10 months prior to progression, 1 remaining alive for 17 months. Nine patients have died. CT was generally well tolerated with <50% patients having mild nausea and vomiting. Marrow toxicity was severe with 23/27 (85.2%) courses causing W.H.O. grade 4 neutropenia (NP) and 24/27 (88.8%) grade 4 thrombocytopenia. A mean of 10U plts and 5U blood were given per patient. Nineteen of 27 (70%) courses were associated with NP fever requiring antibiotics. One patient died of opportunistic infection. The response and acceptable toxicity seen in a conventionally refractory group of patients with this regime warrants further investigation.

### A phase I study of sequential recombinant interferon gamma (IFN-G) and recombinant interleukin-2 (rIL-2) in patients with solid tumours

J. Wagstaff, S.H. Goey, R.L. Scheper, J.B. Vermorken, J.J.M. van der Hoevan & H.M. Pinedo

*Free University Hospital, Amsterdam, The Netherlands.*

Both rIFN-G and rIL-2 have shown anti-tumour activity in single agent phase I studies. *In vitro* studies have demonstrated synergistic anti-tumour activity. T cells activated by rIFN-G developed maximal proliferative responses to rIL-2 after 4 days exposure. This study was designed to determine the maximally tolerated dose and optimum immunomodulatory doses (OID) of 5 daily i.v. bolus (over 15 min) injections of rIL-2 following 5 daily i.m. injections of rIFN-G given in two doses (10 and 100 $\mu$g m$^{-2}$ day) 16 patients have been entered and recruitment will be completed in January 1988. Tumour types were: melanoma (4), renal carcinoma (4), colon carcinoma (3), soft tissue sarcoma (2), mesothelioma (2) and non small-cell lung (1). Nine patients had no prior chemo- or radiotherapy. rIL-2 dose levels were 0.5, 1.0, 2.0 and 3.0 mega(m) U m$^{-2}$ d$^{-1}$. Ten $\mu$g m$^{-2}$ of rIFN-G gave minimal side effects and whilst 100 $\mu$g m$^{-2}$ gave more toxicity no unexpected problems arose. All 3 patients given 3.0 mU m$^{-2}$ d$^{-1}$ experienced WHO grade III/IV toxicity (hypotension, fever and liver toxicity). Two required intensive care management for hypotension. 2.0 mU m$^{-2}$ d$^{-1}$ plus 10 $\mu$g m$^{-2}$ rIFN-G could be used for phase II studies in an ordinary ward setting. So far no objective tumour responses have been seen. A recommendation for phase II will depend on toxicity in the 100 $\mu$g m$^{-2}$ d$^{-1}$ rIFN-G arm and on the OID. Immunological testing will be performed on stored frozen cells in order to ensure assay standardisation (e.g., NK and LAK cell assays).

### The effect of alpha-2 interferon on the pharmacokinetics of ifosfamide

M.J. Lind[1], J.M. Margison[2], N. Thatcher[1] & P.M. Wilkinson[2]

[1]*CRC Department of Medical Oncology and* [2]*Department of Clinical Pharmacology, Christie Hospital and Holt Radium Institute, Manchester M20 9BX, UK.*

It has been shown in several xenograft models that synergy exists between interferon and a wide variety of cytotoxic drugs. The mechanism of this is unknown but it has been speculated that interferon may alter drug biotransformation. Interferon can inhibit cytochrome P450 which is important in the biotransformation of ifosfamide to its active metabolites. We therefore, decided to investigate the effect of alpha-2 interferon on the pharmacokinetics of ifosfamide. Patients with non small-cell lung cancer were given ifosfamide 1.5 g m$^{-2}$ i.v. per day for 5 days during which serial blood samples were taken for assay of ifosfamide levels. Two weeks later they were started on alpha-2 interferon 3 million units SC x 3/week for 2 weeks. During the second week, they were given a second 5 day course of ifosfamide at the same dose as before and further blood samples were taken to study ifosfamide pharmacokinetics. The preliminary results are shown below and do not indicate any great inhibition of drug metabolism.

| | *Pre* | | |
|---|---|---|---|
| *Days* | *1* | *3* | *5* |
| Clearance (ml $min^{-1}$) | 102 | 147 | 175 |
| $t_{1/2}$ (h) | 3.73 | 2.33 | 2.34 |
| AUC ($\mu g$ h $ml^{-1}$) | 510 | 336 | 316 |
| | *Post IFN* | | |
| *Days* | *1* | *3* | *5* |
| Clearance (ml $min^{-1}$) | 88 | 109 | 183 |
| $t_{1/2}$ (h) | 4.15 | 3.80 | 2.40 |
| AUC ($\mu g$ h $ml^{-1}$) | 562 | 412 | 289 |

### The influence of treatment on survival from well differentiated thyroid carcinoma

D.J. Kerr[1], N. Reed[2], G.J. MacFarlane[1], P. Boyle[1] & A.D. Burt[3]

*[1]CRC Department of Medical Oncology and Departments of [2]Radiation Therapy and [3]Pathology, Western Infirmary, Glasgow, UK.*

Thyroid cancer is a relatively rare disease (age standardised incidence rate of approximately 2/100,000 in Scotland) yet there is data to suggest that it has been steadily increasing in incidence over the past 15 years and following the recent incident in Chernobyl it is widely predicted that there will be a large number of excess cases across Europe over the next 3 decades. We have retrospectively analysed 495 cases of thyroid cancer which were treated in Glasgow over the past 30 years and have confirmed, by multivariate analysis, that the prognostic score defined by the EORTC cooperative group is applicable to our patient group. We determined the influence of treatment on overall survival in the well differentiated thyroid cancer patients (199) using a Cox's proportional hazards model to compare treatment subgroups which were balanced according to prognostic score. In summary, the type of thyroid surgery did not influence survival; the addition of radio-iodine to surgery did not influence survival; the addition of external beam radiation to surgery and radio-iodine significantly worsened survival.

### The effect of chemotherapy on CA125 immunohistochemical expression in epithelial ovarian cancer

C.W.E. Redman, M. Bradgate, R. Rollason, C. Hilton, P.A. Canney, F.G. Lawton, K.K. Chan, D. Luesley & G.R.P. Blackledge

*West Midlands CRC Clinical Trials Unit, Queen Elizabeth Hospital, Birmingham, UK.*

CA125 is a glycoprotein found on the surface of most epithelial ovarian carcinomas (EOC), detected using a murine monoclonal antibody OC125. Within reactive tumours, positive and negative cells are found to be intimately mixed demonstrating tumour cell heterogenicity, which has important implications in tumour response. Changes in the histological appearance of tumours have been noted following chemotherapy, possibly reflecting the eradication of chemosensitive subpopulations of cells. Though serum CA125 levels have been shown to closely parallel tumour response, the effect of chemotherapy on the proportion of cells expressing CA125 within a tumour has not been studied.

Serum and histological material were obtained from 19 patients with histologically confirmed EOC, who had undergone primary and post-chemotherapy laparotomies. All patients had had macroscopic residual disease at the end of the first operation and had received either single agent or combination platinum therapy. All patients had macroscopic disease at the second operation though no patients had progressed on treatment. Immunostaining was performed on tissue sections from formalin-fixed paraffin embedded specimens, using OC125 murine monoclonal antibody purchased in kit form (CIS UK). CA125 positivity of the tissue sections were assessed on the basis of intensity and consistency of staining.

Pre-chemotherapy serum CA125 levels were markedly elevated and significantly decreased during treatment. The histological features of tissue obtained at the second operation were unchanged except in three cases of serous cystadenocarcinoma in whom the tissue obtained from the second operation appeared more differentiated. There was no difference in immunostaining between primary tumour and metastatic disease. Ninety-five percent (18/19) of the tumours were CA125 positive. Positivity was observed for all histololgical types, and in the serous sub-group, positivity did not appear to be related to the degree of cellular differentiation. CA125 expression in pre- and postchemotherapy specimens was not significantly altered in the majority of cases. Six cases showed an increase (3) or a decrease (3) in CA125 expression. Whilst a fall in serum CA125 levels reflected a reduction in tumour bulk, there was no correlation between falling serum CA125 levels and a reduction in the degree of tissue CA125 expression. No significant association was noted between survival and change in CA125 positivity. Reduction in serum CA125 levels in responding patients largely reflects a reduction in the tumour bulk as a whole not specifically those cells that release CA125.

### Phase I–II study of carboplatin vincristine methotrexate and bleomycin (COMB) in carcinoma of the cervix

G.J.S. Rustin & E.S. Newlands

*CRC Laboratories, Charing Cross Hospital, London W68RF and Mount Vernon Hospital, Northwood, Middlesex, UK.*

An objective response rate of 71% was obtained using a combination of *cis*-platin 100 mg $m^{-2}$, vincristine 1 mg $m^{-2}$, methotrexate 300 mg $m^{-2}$ and bleomycin 30 mg (POMB) in 24 patients with advanced squamous cell carcinoma of the cervix (*Br. J. Obstet. Gynaecol.*, **94**, 111, 1987). To reduce the toxicity of *cis*-platin, carboplatin was substituted at a dose of 200 mg $m^{-2}$ to be given every 14 days with the other drugs unchanged.

Twenty-four patients with squamous cell carcinoma of the cervix were studied. Their mean age, performance status, site of disease and percentage receiving prior radiotherapy were similar to patients in our POMB study. Two patients received one course of COMB, 8 had 2, and 14 had more than 2 courses. Dosage reductions were made because of renal impairment in 9 patients. Courses of chemotherapy were only delayed because of myelosuppression in 4 patients. Five patients had WHO grade 3 and 4 had grade 4 leukopenia, one had WHO grade 3 and 2 had grade 4 thrombocytopenia.

Two patients died from neutropaenic septicaemia, 1 died from uncontrollable pelvic haemorrhage and 2 did not have evaluable disease. Only 5 of the remaining 19 patients had a partial response (26%, 95 confidence limits 45.7–6.38%). Carboplatin as given in the COMB regimen appears less effective than *cis*-platin containing combinations for squamous cell carcinoma of the cervix.

### Intraperitoneal treatment of human ovarian tumour xenografts with liposome encapsulated muramyl-tripeptide phosphoethanolamine (MTP-PE)

S.T.A. Malik, I. Hart & F. Balkwill

*Imperial Cancer Research Fund, Lincoln's Inn Fields, P.O. Box 123, London WC2A 3PX, UK.*

Muramyl dipeptide (MDP) is a component of bacterial cell walls, which activates macrophages *in vitro* to kill tumour cells. *In vitro*, native MDP is rapidly excreted, and does not induce a significant macrophage antitumour activity even in high doses. To overcome this problem, a lipophilic derivative of MDE, MTP-PE, has been incorporated in liposomes. Systemically administered MTP-PE containing liposomes have been shown to cause tumour regression of syngeneic murine tumours (Fidler, *Cancer Res.*, **45**, 4714, 1985).

A study of liposome encapsulated MTP-PE in the treatment of three human ovarian tumour xenografts (OS, Hu and LA) in nude mice was conducted. Five days after intraperitoneal instillation of xenografts, twice weekly injections of PBS, liposomes only (LP) or liposome encapsulated MTP-PE were given for 4 weeks. The survival data in the three xenografts are shown below:

Mean survival (days)

| | *OS* | *Hu* | *LA* |
|---|---|---|---|
| 1. PBS | 26.5 | 34.5 | 26 |
| 2. LP | 37 | 61 | 28 |
| 3. MTP/PE | >130 | >90 | 39 |

Liposome encapsulated MTP-PE significantly prolonged survival in all three models ($P<0.01$). In the Hu xenografts, placebo liposomes were also associated with increased survival. Further studies to characterise peritoneal cell populations and the possible involvement of Tumour Necrosis Factor in these models are in progress.

### A phase II evaluation of trimelamol ($N^2,N^4,N^6$-trihydroxymethyl-$N^2,N^4,N^6$-trimethylmelamine) in stage III/IV ovarian cancer

I.R. Judson, M.E. Gore, L.A. Gumbrell, K. Balmanno, D.I. Jodrell, T.J. Perren, E. Wiltshaw, P. Blaked & A'H' Calvert

*Institute Cancer Research and Royal Marsden Hospital, London and Sutton, Surrey, UK.*

Trimelamol is an analogue of hexamethylmelamine which does not require metabolic activation. A phase II study was undertaken in patients with recurrent stage III/IV ovarian carcinoma after promising activity had been observed in the phase I studies. Forty-three patients were treated in this study, of whom one was found to have an undifferentiated sarcoma, and was therefore excluded from analysis. Thirty-one of the 40 evaluable patients were stage III and 32 had a WHO performance status of 0, 1 or 2. All patients had undergone prior surgery, and chemotherapy with either *cis*-platin (11/40), carboplatin (11/40) or both (18/40). Only 9 patients received prior radiotherapy. Patients were treated with $800\,mg\,m^{-2}$ for 3 consecutive days repeated every 3 weeks. However, 8 patients had dose reductions becaue of low WBC nadirs ($<2\times10^9\,l^{-1}$), and 5 poor risk patients (e.g. extensive abdominal/pelvic irradiation or extensive prior chemotherapy) also had dose reductions. All dose reductions were to between 600 and $700\,mg\,m^{-2}\times3$. Ninety-six courses of trimelamol were given, the median number of courses received being three. The WBC nadir was similar to that seen at the same dose in the phase I study, i.e. $3.4\times10^9\,l^{-1}$ (range $1.4–9.2\times10^9\,l^{-1}$) with a median nadir day of 14 (range 6–26). The responses seen were CR:1; PR:2; MR:4; NR/PD:31 and NE:2 giving an overall response rate (CR+PR) of 8% for assessable patients. This is disappointing when compared to the 23% (5 PR/22) response rate seen in the patients with refractory ovarian cancer treated at over $600\,mg\,m^{-2}\times3$ in the phase I study, although it does confirm that trimelamol has some activity in this usually refractory group of patients.

### Ifosfamide (I) and ifosfamide + *cis*-platin (P) chemotherapy for advanced cervical carcinoma

R.E. Coleman, J. Clarke, M.L. Slevin, E. Wiltshaw & P.G. Harper

*On behalf of The London Gynaecology Oncology Group, UK.*

The use of chemotherapy is increasing in cervix cancer for both palliation of advanced or relapsed carcinoma of the cervix and as part of primary treatment in poor risk patients. The optimum drug or combination of drugs is not defined. However, like others, we have found I to be an active drug and report our experience with I based first-line chemotherapy in two consecutive series of patients.

Seventy-one patients received I $1.5\,g\,m^{-2}$ daily $\times$ 5 with mesna every 3 weeks. Thirty-nine patients received in addition P $50\,mg\,m^{-2}$ on day 1. Sixty-two patients had been previously irradiated. Of 30 evaluable patients receiving I alone 12 (40%) responded (6 CR and 6 PR, median duration 21 months [range 3–48+ months]); and of 31 receiving I+P (39%) responded (2 CR+10 PR, median duration 7 months [range 5–12 months]). In both series similar response rates were seen in both irradiated and non-irradiated sites.

The major toxicity was bone marrow suppression with 4 (12%) receiving I alone and 10 (26%) I+P developing WHO grade IV leucopenia. All patients developed alopecia and nausea and vomiting was severe (WHO grade III or IV) in 10 (30%) receiving I and 16 (41%) I+P.

I is a useful agent in this disease and a few durable remissions were seen with I alone. The addition of P does not appear to add to the response frequency but increases toxicity. Furthermore durable remissions have not been seen following I+P.

### High dose (HD) carboplatin (JM8) for stage IV ovarian carcinoma: A preliminary analysis of response, toxicity and survival

T.J. Perren, M.E. Gore, I. Fryatt & E. Wiltshaw

*Gynaecology Unit, Royal Marsden Hospital, Fulham Road, London SW3, UK.*

Stage IV ovarian carcinoma (OC) has a poor prognosis. Our previous experience with *cis*-platinum $100\,mg\,m^{-2}$ in 19 such patients gave a 61% response rate, a median survival of 13 months, and only one patient alive beyond 3 years (Wiltshaw *et al., J. Clin. Oncol.*, **4**, 722, 1986). The dose limiting toxicity of JM8 is haematological which makes it attractive for use in high dosage with full support.

In an attempt to improve survival of stage IV OC we treated 38 patients with HD JM8 every 4–5 weeks (14 patients in a dose escalation study – ($mg\,m^{-2}$) 520 (5) 650 (6), 800 (3) patients – and 24 patients at $1\,g\,m^{-2}$). Median age 55 years (39–67). WHO PS 0 (12), 1–2 (20), 3–4 (6) patients. Residual bulk (cm) <2 (4), 2–5 (8), >5 (26) patients. Response, relapse and median time to relapse (MITR) as follows (3 of the $1\,g\,m^{-2}$ patients were not evaluable for response).

| | *Patients* | *CR* | *PR* |
|---|---|---|---|
| Dose esc. | 14 | 4 (2 path) | 3 |
| $1\,gm^{-2}$ | 21 | 4 (2 path) | 9 |

| *Overall* | *Relap.* | *MITR* | |
|---|---|---|---|
| Dose esc. | 7 (50%) | 6 | 11 mos |
| $1\,gm^{-2}$ | 13 (62%) | 5 | 15 mos |

At $1\,gm^{-2}$ the main toxicity was cumulative myelosuppression and 8 patients had treatment stopped as a result. Eleven patients had dose reductions due to a reversible fall in GFR. Main toxicities as shown.

| *WHO grade* | *0* | *1–2* | *3* | *4* |
|---|---|---|---|---|
| Leukopenia | 0 | 2 | 8 | 4 |
| Thrombocytopenia | 0 | 0 | 3 | 14 |
| Infection | 11 | 7 | 4 | 0 |
| Nausea and vomiting | 0 | 19 | 3 | 0 |

Median survival for dose esc. and $1\,gm^{-2}$ patients was 14 and 21 months respectively.

We feel that HD JM8 compares with our previous experience and is worthy of further study.

**Identification of patients at high risk of recurrence after surgical management of stage IB and IIA cervix cancer**

E.J. Burton[1], N. Saunders[2], M. Patterson[2] & G. Blackledge[1]

[1]*CRC Clinical Trials Unit, Queen Elizabeth Hospital, Birmingham; and* [2]*The Northern Hospital, Sheffield, UK.*

Stage specific 5-year survival for treated cervix cancer remain unchanged. In the West Midlands 65% of patients present with stage I disease with a 5-year survival in this group of around 80%. Thus around 20% of patients will present with recurrent disease. In this group of patients 1 year survival is less than 15%. We present a peliminary analysis of 120 surgically treated patients with stage IB and IIA cervix cancer. The aims of the study were: to compare data from patients treated in our practice with published data, to identify and characterize patients at high risk of recurrence who might benefit from adjuvant therapy and to develop a prognostic score which may be applied prospectively to identify these patients prior to conventional therapy. Case records of all patients treated with radical hysterectomy for stage IB/IIA cervix cancer were examined. Demographic histologic, treatment, relapse and survival data were extracted. In 23 of 120 cases recurrence occurred. In 3 cases recurrence was central pelvic, in 18 cases pelvic sidewall, and in 2 cases distant metastases. There was no correlation between recurrence and age at operation, stage, cell type or degree of differentiation. There was a significant association between recurrence and the presence of lymphatic or vascular invasion and pelvic or para-aortic node metastases. It was not possible on the basis of this retrospective analysis to comment on tumour volume. Lymphatic/vascular permeation and lymph node metastases are shown to be associated with a high risk of recurrence. Further research to develop a sensitive and specific scoring system to predict recurrence and identify patients who may benefit from adjuvant treatment is underway.

**Sequential *cis*-platin/cyclophosphamide chemotherapy and abdomino-pelvic radiotherapy in the management of advanced ovarian cancer**

J.A. Green, R.D. Errington, S. Myint, A.J. Slater & H.M. Warenius

*CRC Department of Radiation Oncology, Clatterbridge Hospital, Wirral, Merseyside L63 4JY, UK.*

Following the high response rates achievable with combination chemotherapy in advanced ovarian carcinoma, this pilot protocol employs the addition of abdominopelvic radiotherapy to control residual small volume disease. Forty-six patients with advanced ovarian cancer (6 stage I, 5 stage II, 25 stage III and 9 stage IV) were treated with 5 cycles of *cis*-platin $80\,mg\,m^{-2}$ and cyclophosphamide $1\,gm^{-2}$, given IV every 28 days. All patients were ECOG performance status 0–2 and of 41 evaluable patients for response to chemotherapy there were 31 CR (72%), 4 PR (9%), 4 PD and 2 SD, based on clinical criteria in 23, and second look procedures in 18. Twenty-four patients in clinical or pathological complete remission then received abdominal radiotherapy to a dose of 25 Gy in 25 fractions with posterior renal shielding at 15 Gy followed by a pelvic boost of 20 Gy. Twenty-four patients in CR have completed sequential chemotherapy and radiotherapy. Eighty-five percent of patients received more than 75% of their planned chemotherapy, and the pelvic boost was omitted in 2 patients and suspended in 5 on account of myelosuppression. The median survival of the stage IV patients was 12 months, but has not been reached in those patients achieving a clinical or pathological CR. Of those completing both treatments 75% remain alive and free of disease at a median follow-up of 30 months. In the 15 patients with stage III/IV disease with moderate or poor histological grade given chemotherapy and radiotherapy, the actuarial 2 year survival was 57%. This intensive approach is feasible at the doses of chemotherapy and radiotherapy stated, and achieves a high relapse free survival.

**An open pilot study of the 5-HT$_3$ antagonist, BRL 43694, an effective antiemetic in refractory highly emetogenic cytotoxic drug induced emesis**

L.A. Gumbrell[1], C. Qualmann[1], T.J. Perren[1], G. Forgeson[1], A.H. Calvert[1], S. Thompson[2] & W.G. Rapeport[2]

[1]*Institute of Cancer Research and Royal Marsden Hospital, Surrey and London; and* [2]*Beecham Pharmaceuticals Research Division, Harlow, Essex, UK.*

BRL 43694 is a selective 5-HT$_3$ receptor antagonist which exhibits potent antiemetic activity in ferrets (Boyle *et al.*, *Br. J. Pharmacol.*, **91**, Proc. Supp. 418 p, 1987). Fourteen patients were treated who had had severe nausea and vomiting (N/V) refractory to conventional antiemetics (usually metoclopramide/lorazepam) on previous courses of treatment. They received $20\,\mu g\,kg^{-1}$ (1 patient) and $40\,\mu g\,kg^{-1}$ (13 patients) of BRL 43694 given as a 30 min i.v. infusion, starting 30 min post cytotoxic drug treatment. N/V were assessed on 4-point global scales, nausea was also self-assessed on a visual analogue (VA) scale and emetic episodes were recorded for 24 h after chemotherapy. Results were: *cis*-platin (75–100 $mg\,m^{-2}$ 9 patients) 1 patient had no NV, 2 were free from NV for 12–18 h, 3 had N/V but less than on previous courses and 3 all with anticipatory N/V, derived no benefit. Carboplatin (200 $mg\,m^{-2}$, 3 patients) (+methotrexate and vinblastine): all 3 patients had no M/V. Trimelamol 800 $mg\,m^{-2}$ ×3, 2 patients): both patients had no benefit. Vomiting following BRL 43694 was not always associated with nausea. Using global scales 2 patients reported mild nausea at 4 h and 6 patients had moderate nausea at 24 h. Using VA scales moderate/severe nausea at the time of dosing was seen in 6 patients which persisted to 24 h in 4. The only side effect noted was a mild/moderate headache which occurred in 5 patients. No other adverse effects were noted. Haematology and biochemistry values stayed within normal limits and the ECG (monitored for 30 min) and blood pressure (monitored for 6 h) were unaffected. In summary 9/14 patients with severe N/V refractory to conventional antiemetics experienced benefit from low doses of BRL 46394 compared to their previous courses. Six of these had a substantial improvement. Patients with anticipatory vomiting did not benefit from the treatment schedule used.

### Antiemetic activity of BRL 43694, a selective 5-$HT_3$ antagonist in cancer chemotherapy

J. Cassidy[1], C. Lewis[1], W.G. Rapeport[2], V. Raina[3], M. Soukop[3], E.M. Rankin[1] & S.B. Kaye[1]

[1]*Department of Oncology, University of Glasgow;* [2]*Beecham Pharmaceuticals, Harlow, Essex; and* [3]*Glasgow Royal Infirmary, UK.*

BRL 43694 is a new selective 5-$HT_3$ antagonist, with promising preclinical antiemetic activity. Twenty patients receiving a variety of emetogenic cytotoxics (including *cis*-platin in 5) were given BRL 43694 40 $\mu g\,kg^{-1}$ in this open study. The antiemetic was administered as a single 30 min i.v. infusion between 0 and 6 h after the first course of chemotherapy in successive groups of patients. Standard antiemetics were given for all subsequent courses. Patients completed visual analogue scales (VAS) of nausea, and trained observers assessed nausea, vomiting and adverse effects 4-hourly for 24 h. Seven patients experienced neither nausea nor vomiting. Four patients recorded no nausea on VAS, but were noted by observers to have mild nausea without any vomiting. In 9 patients mild to moderate nausea and vomiting occurred: usually more than 9–12 h after antiemetic administration. Fourteen patients expressed a preference for BRL 43694 over standard antiemetics given with the second course of chemotherapy. In 4 of the patients of whom BRL 43694 was delayed until 4–6 h after chemotherapy, vomiting had already begun to occur, in each case immediate termination of vomiting occurred when BRL 43694 was infused. There was no clear relationship between interval from chemotherapy to BRL 43694 administration and antiemetic efficacy. Mild sedation was noted in 6 cases, though the relationship to drug administration was unclear because of concurrent use of hypnotics and analgesics. No other adverse effects were observed. This study shows BRL 43694 to be an effective and well-tolerated antiemetic. Studies continue to define optimum dosage schedules.

### Phase I–II trial of UFT in the treatment of advanced colorectal gastric and breast cancer

S.T.A. Malik, P. Clark, R. Osborne, D. Talbot, P. Wilson, P.F.M. Wrigley & M.L. Slevin

*ICRF Department of Medical Oncology, St Bartholomew's and Homerton Hospitals, London, UK.*

UFT is a combination of uracil and the 5-fluorouracil (5-FU) analogue Futraful (1-(2-tetrahydrofuryl)-5-fluorouracil). Studies *in vitro* and in experimental tumour models have shown that the cytotoxic effect of fluoropyrimidines can be potentiated by purines and pyrimidines (Fukii *et al.*, *Gann*, **69**, 763, 1978). The mechanism of this effect may be related to the increased intratumoral levels of 5-fluorouracil achieved with concomitant administration of uracil (Taguchi *et al.*, *Cancer Chemother.*, **5**, 1167, 1978). Futraful has greater bioavailability than 5-FU after oral administration. It is metabolised to 5-fluorouracil and at least four other compounds (Au, *Cancer Treat. Rep.*, **63**, 343, 1979). As it has a long half-life (6–16 h), it may act as a slow-release preparation of 5-FU. A phase I–II study of oral UFT (12 $mg\,kg^{-1}$ day, max. dose 600 $mg\,day^{-1}$) given as three divided doses is in progress, 33 patients with advanced colorectal cancer, 9 with advanced stomach cancer, 11 with advanced breast cancer have been treated. The results in the currently evaluable patients are tabulated below.

| | *N* | *Previous chemotherapy* | *PR* | *CR* |
|---|---|---|---|---|
| 1. Colorectal cancer | 26 | 3 | 3 | 0 |
| 2. Stomach cancer | 6 | 0 | 0 | 0 |
| 3. Breast cancer | 8 | 5 | 1 | 0 |

Partial responses in colon cancer patients lasted 9, 16 and 60 weeks. Myelosuppression was not seen and non-haematological toxicity was minimal. *Conclusion:* Oral UFT is well tolerated but the study suggests that its antitumour activity is not substantially different from that of 5-fluorouracil.

### Phase II study of methyl acetylenic putrescine in colorectal carcinoma biochemical effects

J. Carmichael[1], D. Khayat[1], C. Jacquillat[1], G. Tell[1], A. Skillen[1] & A.L. Harris[1]

*Departments of* [1]*Clinical Oncology and* [2]*Biochemistry, Newcastle upon Tyne, UK;* [3]*Medical Oncology, Hosp. Salpetriere, Paris; and* [4]*Merrell Dow Research Institute, Strasbourg, France.*

Sixteen patients with advanced colorectal carcinoma were treated with the ornithine decarboxylase inhibitor methyl acetylenic putrescine (MAP) 250 mg tds for 8 weeks. There were 13 male and 3 female patients with a mean age of 58.8 years. Ten patients had hepatic metastases and 5 pulmonary metastases. Investigations performed included weekly FBC, U+E, LFT's and creatinine clearance. Urine was assayed weekly: for measurement of urinary protein, alanine aminopeptidase and N-acetyl $\beta$-glucose aminidase (NAG). Urinary excretion of decarboxylated S-adenosyl-L-methionine (dC-SAM) was measured as an index of biochemical activity. No therapeutic responses were seen, but stable disease was observed in 6 patients. At this dosage the drug was relatively well tolerated with mild epigastric pain and general malaise most frequently reported. In a sub-group of patients, the mean level of dc-SAM prior to treatment was 1,393 $nmol\,24\,h^{-1}$; 3,395 at week 1; 5,565 at week 2; and 8,126 $nmol\,24\,h^{-1}$ at week 3, representing a 6.7-fold increase. Urinary NAG and creatinine clearance results indicated transient alterations of renal function in 2 patients on MAP. MAP had minimal haematological toxicity, although 1 patient had transient thrombocytopoenia, at 6 weeks, which was not clinically significant. In 6 patients following 4 weeks MAP therapy there was a decrease in total lymphocyte count.

MAP 250 mg tds was well tolerated and caused the expected biochemical effect. Despite the lack of activity in this resistant tumour type, this drug is worthy of further study in view of its interesting biochemical activity, although close attention should be paid to renal function in these patients.

**A phase II study of carboplatin in adenocarcinoma of the oesophagus**

A. Steel[1], M.H. Cullen[1], P. Robertson[1] & H.R. Matthews[1]

[1]*East Birmingham Hospital and* [2]*Queen Elizabeth Hospital, Birmingham, UK.*

Carboplatin has been evaluated as a single chemotherapeutic agent in 15, previously untreated, male patients with adenocarcinoma of the oesophagus. Ages ranged from 49 to 72 (median 62) and all tumours except one were in the lower thoracic oesophagus. The patients were all of good performance status (WHO 0 or I). Patients were staged with thoraco-abdominal CT scan before chemotherapy and again after two courses for response assessment. Pre-treatment radiological staging was as follows: One case $T_1$, 11 cases $T_2$, 2 cases $T_3$; 4 cases had $N_1M_1$ disease in addition. Carboplatin was used at a dose of 400 mg m$^{-2}$ i.v. infusion over 30 min repeated after 28 days. Twelve patients are currently evaluable for treatment response. Nine patients have shown no change in the size of their primary tumour and 3 have shown progression. One patient died from gastro-intestinal haemorrhage after the second course and 2 patients have not completed therapy. Eight patients have undergone resection of their tumours following chemotherapy, with one post-operative death. Toxicity was mild and manageable. Nausea and vomiting WHO grade I was recorded in 6 cases and WHO grade II in 5 cases. Four patients experienced no nausea or vomiting. Grade I haematological toxity on day 28 affected 7 patients with 1 or both courses and grade II or III toxicity affected 1 patient each. There were no cases of severe, permanent nephrotoxicity. Carboplatin in this dosage and schedule is ineffective as a single agent in adenocarcinoma of the oesophagus.

**Clinical and preclinical pharmacokinetic studies with 1-(4-carboxyphenyl)3,3-dimethyltriazene (CB10-277)**

B.J. Foster[1], D.R. Newell[1], J. Carmichael[2], A.L. Harris[2], L.A. Gumbrell[1], D.E.V. Wilman[1] & A.H. Calvert[1]

[1]*Institute of Cancer Research, Sutton, Surrey; and* [2]*Newcastle General Hospital, Newcastle upon Tyne, UK.*

CB10-277 is an analogue of dacarbazine (DTIC) which is currently undergoing phase I clinical evaluation. In experimental systems CB10-277 and DTIC have equivalent antitumour efficacy and CB10-277, like DTIC, requires metabolic activation in order to display activity. As part of the preclinical and early clinical evaluation of CB10-277 the pharmacokinetics of the drug in patients and mice have been compared. In mice, following a maximum tolerated dose (750 mg m$^{-2}$ i.v.), CB19-277 was cleared monophasically from plasma (25 ml min m$^{-2}$, $t_{1/2}$ 32 min). In addition to CB10-277 one major and two minor metabolites were found in plasma using HPLC analysis. The major metabolite was sensitive to hydrolysis by glucuronidase yielding CB10-277 whilst one of the minor metabolites was identified by HPLC as the monomethyl derivative of CB10-277. Monomethyl CB10-277 is the proposed active metabolite of the drug and the levels observed in plasma (>50 $\mu$M for 1 h) are sufficient to explain the antitumour activity seen with CB10-277 in mice. In an ongoing phase I study 12 patients have received 24 courses of CB10-277 with doses escalated from 80 to 1,350 mg m$^{-2}$ (i.v. bolus). Toxicities have been mild, i.e. nausea and vomiting WHO grade I 3/24, grade II–III 3/24 and rashes grade I 1/24, grade II–III 2/24. No haematological toxicity has been seen. Pharmokinetic analyses have been performed on 20 courses. CB10-277 plasma clearance (74±30 ml min$^{-1}$ m$^{-2}$, mean±s.d.) was biphasic (terminal $t_{1/2}$ 79±36 min). Two major CB10-277 metabolites have been detected in human plasma. Of these, one is the glycine conjugate of CB10-277 whilst the other has HPLC properties similar to the glucuronidase sensitive metabolite seen in mice and is the major plasma metabolite following doses of >600 mg m$^{-2}$. In addition, at 900 and 1,350 mg m$^{-2}$, monomethyl CB10-277 may be present (levels <10 $\mu$M). These studies have shown that, qualitatively, CB10-277 metabolism is similar in mice and in patients. Thus clinical antitumour activity may be anticipated and has in fact been observed with 1 minor and 2 partial responses out of 4 melanoma patients treated.

**Methotrexate (M), vinblastine (V), adriamycin (A) and *cis*-platin (C) for advanced bladder cancer**

C.J. Twelves[1], J.A. Clarke[1], R.E. Coleman[1], R. Stuart-Harris[1], T. Oliver[2] & P.G. Harper[1]

[1]*Clinical Oncology Unit, Guy's Hospital and* [2]*The London Hospital, Whitechapel, London, UK.*

Bladder cancer is generally not considered to be a chemosensitive tumour, but recently a high response rate and prolonged survival have been reported in patients with advanced bladder cancer treated with M-VAC chemotherapy (Sternberg *et al.*, *J. Urol.*, **133**, 403, 1985). We undertook a phase II study to confirm these results.

Treatment was M 30 mg m$^{-2}$ i.v. days 1, 15 and 22; V 3 mg m$^{-2}$ i.v. days 2, 15 and 22; A 30 mg m$^{-2}$ i.v. day 2; and C 70 mg m$^{-2}$ i.v. day 2. Maximum of 6 cycles was given at 4 weekly intervals. If the creatinine clearance was under 40 ml min$^{-1}$, C was withheld (3 patients) or carboplatin was given (4 patients) at a dose adjusted for renal function. Patients were assessed after 3 and 6 cycles of chemotherapy.

Twenty-six patients with transitional cell carcinoma (2 advanced primary cancer alone and 24 metastatic disease) were treated. Thirteen had progressed after DXT to the primary site and 2 had received prior chemotherapy. Median age was 64 years (range 34–75), 22 male and 4 female. Median ECOG status at entry was 2 (0=5 patients, 1=7 patients, 2=5 patients, 3=9 patients).

Of the 26 patients assessable to date, 3 had complete response and 10 partial response. The overall response rate was 13/26 (50%) and median response duration was 30 weeks. Responses was seen in the bladder (44%) including 3 patients who had progressed after DXT, lymph nodes (68%), lung (37%) and liver (25%). There were 2 early deaths (1 toxic and 1 sudden at home). All patients are evaluable for toxicity; 11 had WHO grade III or IV vomiting and 16 had WHO grade III or IV myelosuppression.

M-VAC chemotherapy is effective in this group of poor prognosis patients with advanced bladder cancer. Toxicity is significant, but M-VAC gives useful palliation for many patients.

**Haematological recovery after high dose cyclophosphamide (CTX) and autologous bone marrow transplantation (ABMT)**

C.J. Twelves[1], R.L. Souhami[2], P.G. Harper[1] & A. Goldstone[3]

[1]*Clinical Oncology Unit, Guy's Hospital and Departments of* [2]*Oncology and* [3]*Haematology, University College Hospital, London, UK.*

ABMT is widely used to reduce myelosuppression after high dose alkylating agent, but often with little firm evidence of its value. We have studied haematological recovery in 31 fit, previously untreated small cell lung cancer patients receiving high dose CTX with ABMT.

Chemotherapy was CTX, either 160 mg kg$^{-1}$ or

200 mg kg$^{-1}$ i.v. over 4 successive days. Patients received either a single cycle of high dose CTX with ABMT or 2 cycles of CTX with ABMT. Bone marrow was harvested before initial chemotherapy in all patients and reinfused after each cycle of high dose CTX. In most patients ABMT was 48 h after completing chemotherapy, designated day 2. In an attempt to evaluate ABMT, reinfusion was delayed to day 4, 6 or 8 after the first cycle of CTX 200 mg kg$^{-1}$ in some patients.

Treatment groups and duration of myelosuppression were as follows:

| *Chemotherapy* | *No. pts* | *ABMT (day)* | *Neutrophils* $<500 \times 10\,l^{-1}$ *(days)* | *Platelets* $<500 \times 10\,l^{-1}$ *(days)* |
|---|---|---|---|---|
| CTX 160 mg kg$^{-1}$ | | | | |
| (cycle 1) | 7 | 2 | 9.6 | 1.7 |
| CTX 200 mg kg$^{-1}$ | 11 | 2 | 10.6 | 6.7 |
| (cycle 1) | 3 | 4 | 12.0 | 4.8 |
| | 8 | 6 | 13.7 | 9.3 |
| | 2 | 8 | 14.5 | 9.5 |
| CTX 200 mg kg$^{-1}$ | | | | |
| (cycle 2) | 11 | 2 | 14.8 | 14.5 |

ABMT contributes to haematological recovery when CTX 200 mg kg$^{-1}$ is given over 4 days. However, myelosuppression is more prolonged after cycle 2, suggesting recovery is more dependent on ABMT after the second cycle of CTX, or that there is residual stromal damage after initial chemotherapy.

### Oral idarubicin in the treatment of acute myelogenous leukaemia (AML) and chronic myeloid leukaemia (CML) accelerated phase/blast crisis

S.T.A. Malik, J. Tucker, W. Brace, A.Z.S. Rohatiner & T.A. Lister

*ICRF Department of Medical Oncology, St Bartholomew's Hospital, West Smithfield, London EC1A 7BE, UK.*

Idarubicin is a daunorubicin analogue that has been demonstrated to have antileukaemic activity after intravenous administration (Dagnestini *et al.*, *Cancer Res.*, **45**, 1408, 1985). It is also active when given orally. A phase I–II study of oral idarubicin (25 mg m$^{-2}$ day$^{-1}$ for 3 days) was conducted in patients with AML and CML in the accelerated phase or blast crisis.

The details of the AML patients are as follows: Total no. = 14, previously treated = 12 (4 relapsed, 8 resistant), previous myelodysplasia = 5, mean age 58 (range 18–81), median white cell count = $72.5 \times 10^9\,l^{-1}$, median blast count = $65 \times 10^9\,l^{-1}$.

The CML group comprised: 2 myeloid blast crisis, 1 megakaryocytic/erythroid blast crisis, and 3 accelerated phase, mean age 49 (range 32–60), median white cell count = $184 \times 10^9\,l^{-1}$ (range $28–340 \times 10^9\,l^{-1}$).

No complete remissions were seen. The first course of idarubicin was associated with a decrease in both the peripheral wbc and blast count by day 4 in all patients. Nadir counts were reached at 4–7 days in AML patients, and 14–33 days in CML patients. All responses were transient, with an eventual rise in peripheral white cell and blast counts. One patient with AML received 3 courses of idarubicin before becoming resistant, 5 patients with CML received 2 to 6 courses of idarubicin before resistance was seen. Survival in the AML patients ranged from 11–150 days, and 11–97 weeks in CML patients. The non-haematological toxicity was principally mild to moderate nausea and vomiting and fatigue (in CML patients). Most patients were able to take treatment at home, although only 1 AML patient did not have to be admitted to hospital for supportive therapy. Five of the CML patients were able to spend the majority of their time at home, and two patients returned to work.

The results suggest that oral idarubicin can be a useful drug for palliative treatment in CML blast crisis and in some patients with resistant AML.

*The principal sponsors of the meetings were:*

Bristol–Myers Oncology UK, Boehringer Ingelheim Ltd, Ciba–Geigy Pharmaceuticals, Cyanamid (Lederle) Farmitalia Carlo Erba Ltd and ICI Pharmaceuticals (plc).

*Additional sponsorship was received from:*

Anachem Ltd, Applied Biosystems, Becton Dickinson UK, Beecham Pharmaceuticals, Bio-Rad Ltd, Boehringer London, Book Show, Cambridge Research Biochemicals, Cambridge University Press, Camlab, Cell.-Tech, Flow Laboratories, Gibco, ICI Pharmaceuticals (UK), IRL Press, Jencons, Kirby–Warrick, Laser Laboratory Systems Ltd, Lilly Industries, Lundbeck, McCarthy Medical, Meditec, Millipore Biosystems, New Brunswick, Norwich Eaton, Nycomed and Sera-Lab Ltd.