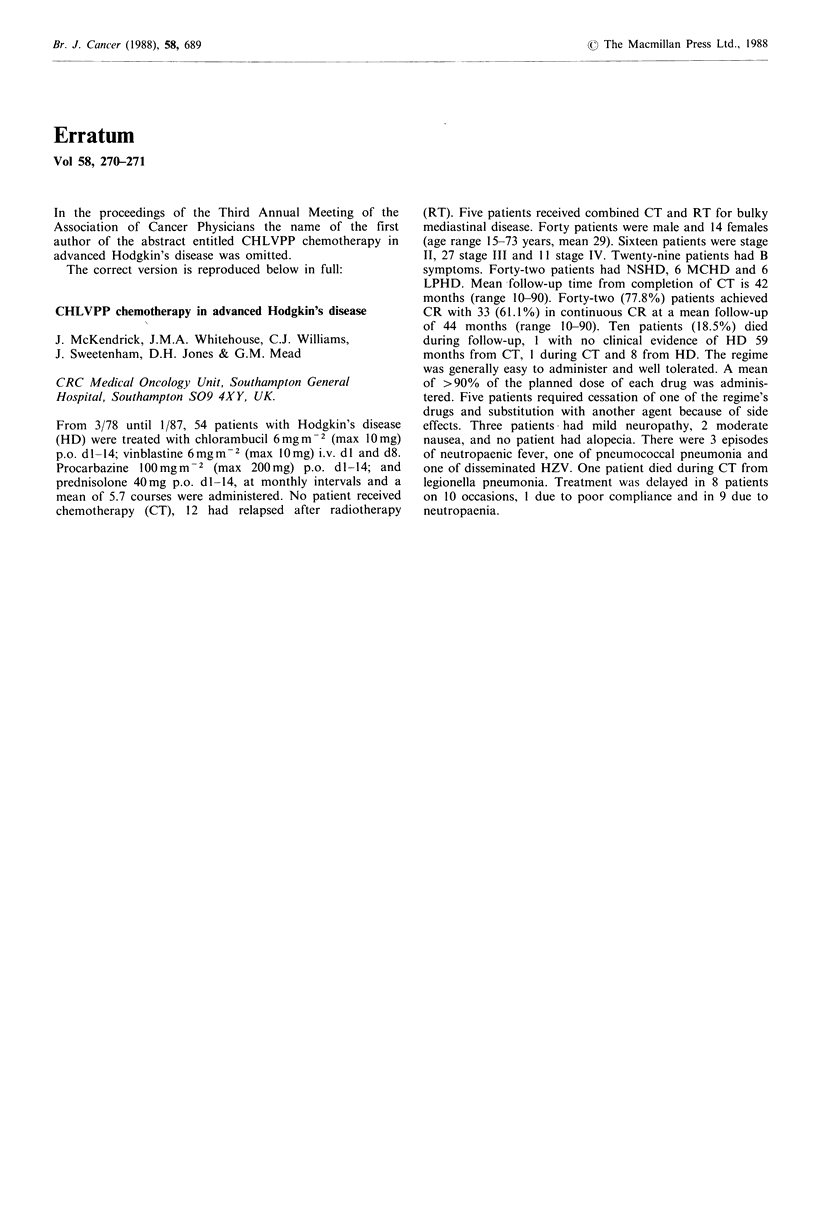# Erratum

**Published:** 1988-11

**Authors:** 


*Br. J. Cancer* (1988), **58**, 689 © The Macmillan Press Ltd., 1988

### Erratum

**Vol 58, 270–271**

In the proceedings of the Third Annual Meeting of the Association of Cancer Physicians the name of the first author of the abstract entitled CHLVPP chemotherapy in advanced Hodgkin's disease was omitted.

The correct version is reproduced below in full:

### CHLVPP chemotherapy in advanced Hodgkin's disease

J. McKendrick, J.M.A. Whitehouse, C.J. Williams, J. Sweetenham, D.H. Jones & G.M. Mead

*CRC Medical Oncology Unit, Southampton General Hospital, Southampton SO9 4XY, UK.*

From 3/78 until 1/87, 54 patients with Hodgkin's disease (HD) were treated with chlorambucil $6\,mg\,m^{-2}$ (max 10 mg) p.o. d1–14; vinblastine $6\,mg\,m^{-2}$ (max 10 mg) i.v. d1 and d8. Procarbazine $100\,mg\,m^{-2}$ (max 200 mg) p.o. d1–14; and prednisolone 40 mg p.o. d1–14, at monthly intervals and a mean of 5.7 courses were administered. No patient received chemotherapy (CT), 12 had relapsed after radiotherapy (RT). Five patients received combined CT and RT for bulky mediastinal disease. Forty patients were male and 14 females (age range 15–73 years, mean 29). Sixteen patients were stage II, 27 stage III and 11 stage IV. Twenty-nine patients had B symptoms. Forty-two patients had NSHD, 6 MCHD and 6 LPHD. Mean follow-up time from completion of CT is 42 months (range 10–90). Forty-two (77.8%) patients achieved CR with 33 (61.1%) in continuous CR at a mean follow-up of 44 months (range 10–90). Ten patients (18.5%) died during follow-up, 1 with no clinical evidence of HD 59 months from CT, 1 during CT and 8 from HD. The regime was generally easy to administer and well tolerated. A mean of >90% of the planned dose of each drug was administered. Five patients required cessation of one of the regime's drugs and substitution with another agent because of side effects. Three patients had mild neuropathy, 2 moderate nausea, and no patient had alopecia. There were 3 episodes of neutropaenic fever, one of pneumococcal pneumonia and one of disseminated HZV. One patient died during CT from legionella pneumonia. Treatment was delayed in 8 patients on 10 occasions, 1 due to poor compliance and in 9 due to neutropaenia.